# Metabolic Rearrangements Causing Elevated Proline and Polyhydroxybutyrate Accumulation During the Osmotic Adaptation Response of *Bacillus megaterium*

**DOI:** 10.3389/fbioe.2020.00047

**Published:** 2020-02-21

**Authors:** Thibault Godard, Daniela Zühlke, Georg Richter, Melanie Wall, Manfred Rohde, Katharina Riedel, Ignacio Poblete-Castro, Rainer Krull, Rebekka Biedendieck

**Affiliations:** ^1^Institute of Biochemical Engineering, Technische Universität Braunschweig, Braunschweig, Germany; ^2^Institute of Microbiology, Universität Greifswald, Greifswald, Germany; ^3^Central Facility for Microscopy, Helmholtz Centre for Infection Research, Braunschweig, Germany; ^4^Biosystems Engineering Laboratory, Center for Bioinformatics and Integrative Biology, Faculty of Life Sciences, Universidad Andres Bello, Santiago, Chile; ^5^Braunschweig Integrated Centre of Systems Biology (BRICS), Technische Universität Braunschweig, Braunschweig, Germany; ^6^Center of Pharmaceutical Engineering (PVZ), Technische Universität Braunschweig, Braunschweig, Germany; ^7^Institute of Microbiology, Technische Universität Braunschweig, Braunschweig, Germany

**Keywords:** *Bacillus megaterium*, osmotic stress adaptation, transcriptomics, proteomics, flux analysis, proline, polyhydroxybutyrate (PHB)

## Abstract

For many years now, *Bacillus megaterium* serves as a microbial workhorse for the high-level production of recombinant proteins in the g/L-scale. However, efficient and stable production processes require the knowledge of the molecular adaptation strategies of the host organism to establish optimal environmental conditions. Here, we interrogated the osmotic stress response of *B. megaterium* using transcriptome, proteome, metabolome, and fluxome analyses. An initial transient adaptation consisted of potassium import and glutamate counterion synthesis. The massive synthesis of the compatible solute proline constituted the second longterm adaptation process. Several stress response enzymes involved in iron scavenging and reactive oxygen species (ROS) fighting proteins showed higher levels under prolonged osmotic stress induced by 1.8 M NaCl. At the same time, the downregulation of the expression of genes of the upper part of glycolysis resulted in the activation of the pentose phosphate pathway (PPP), generating an oversupply of NADPH. The increased production of lactate accompanied by the reduction of acetate secretion partially compensate for the unbalanced (NADH/NAD^+^) ratio. Besides, the tricarboxylic acid cycle (TCA) mainly supplies the produced NADH, as indicated by the higher mRNA and protein levels of involved enzymes, and further confirmed by ^13^C flux analyses. As a consequence of the metabolic flux toward acetyl-CoA and the generation of an excess of NADPH, *B. megaterium* redirected the produced acetyl-CoA toward the polyhydroxybutyrate (PHB) biosynthetic pathway accumulating around 30% of the cell dry weight (CDW) as PHB. This direct relation between osmotic stress and intracellular PHB content has been evidenced for the first time, thus opening new avenues for synthesizing this valuable biopolymer using varying salt concentrations under non-limiting nutrient conditions.

## Introduction

*Bacillus megaterium* is a big rod-shaped Gram-positive soil bacterium that has been used industrially for decades and whose product portfolio is continuously growing. It includes enzymes such as α- and β-amylases, penicillin G acylase, xylanase, hydrolases, glycosyltransferases, and cytochrome monooxygenases (Vary et al., 2007; Korneli et al., 2013; Lakowitz et al., 2017; Mayer et al., 2019). The bacterium displays several important biotechnological features such as its non-pathogenic character, broad substrate spectrum, high plasmid stability, and high secretion capacity for proteins. The development of efficient expression vectors has driven the emergence of *B. megaterium* as a significant industrial workhorse for recombinant protein production (Rygus and Hillen, 1991; Biedendieck et al., 2007b; Stammen et al., 2010; Biedendieck, 2016). Nevertheless, the limited knowledge of its metabolic adaptation strategies still restricts the detection of corresponding bottlenecks during protein production. However, the recent sequencing of the complete genome of different *B. megaterium* strains and the fast development of related omics-techniques have laid the foundations for an in-depth analysis from gene expression to metabolic fluxes, and opened up new possibilities toward its rational genetic engineering (Biedendieck et al., 2011; Eppinger et al., 2011; Liu et al., 2011; Johnson et al., 2015; Freedman et al., 2018).

Fluctuation in water potential is one of the typical issues with whom bacterial cells have to cope in both their natural environment, during drought and rainy periods, and in industrial bioreactors in which fed substrate, expected product and side-product concentrations are particularly high (Schweder et al., 1999). In the latter case, increased medium osmolarity generates a substantial water efflux from cells, which eventually leads to a reduction of overall bacterial performance through cell dehydration and related impaired metabolism and growth (Korber et al., 1996; Kempf and Bremer, 1998). As water is essential for almost every cellular process from gene translation to protein folding processing, bacteria must be able to restore the cellular water homeostasis in order to survive swiftly. One meaningful way to reduce osmolarity gradients and maintain a suitable cell volume relies on active import, production, and secretion of diverse osmolytes (Kempf and Bremer, 1998; Hoffmann and Bremer, 2017).

Under hyperosmotic stress, bacteria of *Bacillus* species require a variety of molecular and metabolic adjustments for survival. Many studies have described that in *Bacillus subtilis*, *Bacillus cereus*, and *Bacillus licheniformis* adaptation to ionic hyperosmotic stress is a two-step process during which cells first transiently import potassium ions quickly as an initial emergency response to a fast osmotic up-shock before replacing these ions ultimately by organic compounds such as sugars, amino acids, polyols and their derivatives such as betaine and ectoine that do not interfere with cellular physiology and biochemistry. Intracellular accumulation of these compounds, known as compatible solutes or osmoprotectants, to molar concentrations enables water retention and turgor maintenance indispensable for proper cell function during salt stress. The cells either import these compounds or synthesize them *de novo*. Besides their role in compensating osmolarity gradients, they also undertake protecting, stabilizing and catalyzing functions, that make them attractive for industrial applications in fields such as cosmetics, health care and biotechnology (Gröger and Wilken, 2001; Graf et al., 2008; Takagi, 2008; Oren, 2010; Hoffmann et al., 2013; Hoffmann and Bremer, 2017). The global response to salt stress in *Bacillus* species additionally involves various other biological processes such as cell wall modification, iron metabolism and redox balancing (Steil et al., 2003; Höper et al., 2006; den Besten et al., 2009; Hahne et al., 2010; Schroeter et al., 2013).

Here, we studied the impact of osmotic stress on the metabolism of the wild-type *B. megaterium* DSM319 during unlimited growth. The metabolic response of this strain to sustained salt stress (up to 1.8 M NaCl) was characterized combining whole-genome expression, intracellular proteome analyses, intra- and extracellular metabolomics as well as *in vivo* fluxomics of the central carbon metabolism, which together explains the underlying mechanism toward elevated proline and polyhydroxybutyrate content in *B. megaterium* cell.

## Materials and Methods

### Bacterial Strain and Growth Conditions

The wild type strain *B. megaterium* DSM319 used for all experiments was obtained from the German collection of microorganisms and cell cultures (DSMZ, Braunschweig, Germany). Cells were stepwise adapted to each cultivation condition and glycerol stocks (20% v/v) were prepared and stored at −80°C.

For pre-cultures and main cultures, a modified M9 minimal medium derived from Harwood and Cutting (1990) was used containing 5 g L^–1^ of glucose, 1 g L^–1^ of NH_4_Cl, 3 g L^–1^ of KH_2_PO_4_, 500 mg L^–1^ NaCl, 6.7 g L^–1^ of Na_2_HPO_4_, 1 mg L^–1^ of MnCl_2_⋅4 H_2_O, 1.7 mg L^–1^ of ZnCl_2_, 430 μg L^–1^ of CuCl_2_⋅2 H_2_O, 328 μg L^–1^ of CoCl_2_, 600 μg L^–1^ of NaMoO_4_⋅2 H_2_O, 11.1 mg L^–1^ of CaCl_2_, 30 mg L^–1^ of 3,4-DHB, 13.5 mg L^–1^ of FeCl_3_ and 120 mg L^–1^ of MgSO_4_. In addition to the 8.6 mM NaCl present in the M9 medium, up to 1.8 M of NaCl were additionally supplemented to this medium where indicated. For labeling experiments, unlabeled glucose was replaced by 99% 1-^13^C-glucose or a mixture of 50% U-^12^C/50% U-^13^C glucose (Cambridge Isotope Laboratories Inc., Andover, MA, United States) for both the pre-cultures and main cultures, thus ensuring a biomass labeling grade superior to 99.5%. All cultivations were performed at 37°C and at least in triplicates (*n* = 3) as indicated in Supplementary Material.

### Transcriptome Analysis

RNA extraction and purification were carried out following the protocols proposed by Biedendieck et al. (2011). The RNA concentration was subsequently determined with a NanoDrop (Peqlab Biotechnologie GmbH, Erlangen, Germany) and RNA integrity was assayed using a Bioanalyzer (Agilent Technologies, Böblingen, Germany) according to the manufacturer’s instruction. Microarrays were prepared with RNA originating from 4 biological replicates (*n* = 4), whose RNA integrity number (RIN) were equal to or greater than 9, and designed for dual labeling. First, RNA from reference and evaluated condition were labeled with two different dyes using the “USL Fluorescent labeling kit” according the supplied instructions (Kreatech, Amsterdam, Netherlands). Dye incorporation rate was determined with the NanoDrop. Subsequently, 300 ng of labeled RNA from both conditions were mixed and RNA was further processed using the “Gene Expression Hybridization Kit” (Agilent technologies, Waldbronn, Germany). Then, samples were loaded on an Agilent microarray slide (8 × 15 K custom made) comprising 2–3 60 bp DNA probes for each gene of *B. megaterium* and hybridization took place for 17 h at 65°C and 10 min^–1^ in a hybridization oven (Agilent Technologies, Waldbronn, Germany). Finally, slides were washed with the gene expression wash buffer kit (Agilent Technologies, Waldbronn, Germany) and scanned using the Agilent C scanner associated to its proprietary software Agilent Scan Control 8.4.1 and Feature Extraction 10.7.3.1. Generated data were post-processed in R with Bioconductor for statistical analysis, including an estimation of measurement relevance using analysis of variance (ANOVA) and eliminating aberrant values from the analysis (adjusted *p*-values > 0.05). All complete experimental data sets were deposited in the GEO database with the accession number GSE110712.

### Proteome Analysis

For proteome analysis, cells were collected in the mid-exponential phase and washed with TE-Buffer (10 mM TRIS, 1 mM EDTA, pH 8). Intracellular proteins were then extracted by mechanical cell disruption (3 × 1 min, 6.5 m⋅s^–1^, 4°C, FastPrep^®^ -24, MP Biomedical, Santa Ana, CA, United States) using soda-lime glass beads (20% v/v, 0.038-0.045 mm, Worf Glaskugeln GmbH, Mainz, Germany) and protein concentration was determined (Roti^®^ Nanoquant, Carl Roth GmbH, Karlsruhe, Germany). Prior to measurement by LC-IMS^E^, protein extracts were digested with trypsin (Promega, Madison, WI, États-Unis) as described previously (Muntel et al., 2012) and peptide solution was then desalted by stage tip purification using a standard protocol (Rappsilber et al., 2007). For absolute quantification, the peptide mix was spiked with tryptic yeast alcohol dehydrogenase (Waters, Milford, MA, United States) at a final concentration of 50 fmol μL^–1^.

Peptide separation, identification and quantification were completed using a NanoACQUITY^TM^ UPLC^TM^-system (Waters, Milford, MA, United States) coupled to a Synapt-G2 mass spectrometer (Waters, Milford, MA, United States). Samples were loaded at a flow rate of 0.3 μL min^–1^ onto an analytical column (nanoACQUITY^TM^ UPLC^TM^ column, BEH130 C18, 1.7 μm, 75 μm/200 mm, Waters, Milford, MA, United States) and separation of peptides for IMS^E^ was achieved using a 90 min gradient from 5% to 26% buffer B (0.1% acetic acid in acetonitrile). All MS^E^ analyses were performed as previously described (Muntel et al., 2014), except that the collision energy was alternated between 4 eV in the precursor ion trace and a ramp of 25–45 eV for fragment ion trace. In addition, wave velocity was ramped from 1,000 to 400 m s^–1^ and wave height was set to 40 V.

Collected LC-IMS^E^ data were imported in the ProteinLynx Global Server 2.5.3-Software (PLGS, Waters, Milford, MA, United States) and further processed with the Apex3D-Algorithmus setting parameters as follows: chromatographic peak width and MS TOF resolution were set to automatic, lock mass charge 2 set to 785.8426 Da/e with a lock mass window of 0.25 Da, low and elevated energy threshold were set at 200.0 and 20.0 counts, respectively, and intensity limit at 750 counts.

Peptide sequence identification was performed with the “ion accounting” algorithm using a randomized Uniprot *B. megaterium* DSM319 database (Version August 2011) comprising all employed laboratory contaminants and the sequence of yeast alcohol dehydrogenase (ADH1 – 10,286 entries). Next criteria were set for positive protein identification: 1 fragment ion matched per peptide, 5 fragment ions matched per protein, 1 peptide matched per protein, 2 missed cleavage allowed, primary digest reagent: trypsin, fixed modification: carbamidomethylation C (+57.0215), variable modifications: deamidation N, Q (+0.9840), oxidation M (+15.9949), pyrrolidone N-TERM (-27.9949). The protein false discovery rate was set to 5%. Only 2 peptide identifications were considered for final analysis. Subsequently, MS data were corrected for detector saturation effects as described earlier (Zühlke et al., 2016).

To ensure statistical relevance, the data were strictly filtered in that only proteins found in at least 2 of 3 biological replicates per time point and 2 of 3 of the corresponding technical replicates were selected for quantification, thus reducing FDR on protein level to less than 0.3%. Finally, determined concentrations were averaged over technical replicates and submitted to a Student’s *t*-test (*p* < 0.01) to estimate the significance of detected modifications of protein concentrations. The mass spectrometry proteomics data have been deposited to the ProteomeXchange Consortium via the PRIDE (Perez-Riverol et al., 2019) partner repository with the dataset identifier PXD015605.

### Metabolome Analysis

Intracellular metabolites from the central carbon metabolism were separated by ion exclusion chromatography using a liquid chromatography system (LC, Agilent 1290, Agilent Technologies, Waldbronn, Germany) equipped with a reverse phase column (VisionHT C18 HL, 100 mm × 2 mm I.D., 1.5 μm, Grace, Columbia, MD, United States) and quantified with a triple quadrupole mass spectrometer (QTRAP 5500, AB Sciex, Darmstadt, Germany) equipped with a TurboIonSpray source. Ten μL of sample were injected to the column and metabolite separation was performed at 50°C using a mixture of 6 mM of aqueous tributylamine solution (Eluent A, adjusted to pH 6.2 with acetic acid) and aqueous acetonitrile solution (50% v/v) supplemented with 6 mM of tributylamine (Eluent B, adjusted to pH 6.2 with acetic acid) as mobile phase. Composition of this mobile phase was gradually varied along the measurement. Separated compounds were introduced at a flow rate of 350 μL min^–1^ into the mass spectrometer (MS) via the turbo ionspray source and detection was completed by multiple reaction monitoring with the MS operating in its negative ionization mode. Besides, the MS was run in unit resolution to achieve the best possible selectivity and sensitivity. Regarding the other key MS-parameters, the entrance potential was set at −10 V, the dwell time was fixed at 5 ms for all transitions, the auxiliary gas temperature was adjusted to 550°C and the source dependent parameters were set to: ionspray voltage −4500 V, nebulizer gas (GS1) auxiliary gas (GS2), curtain gas (CUR) and collision gas CAD 60, 60, 35 medium, respectively.

Intracellular amino acids were extracted from samples taken along the exponential phase and quantified by HPLC as described before (Kromer et al., 2005; Korneli et al., 2012). Using the same extraction protocol, intracellular potassium could be quantified with a Dionex-ICS 2000 HPLC system (Thermo Fischer Scientific, Waltham, MA, United States) equipped with a Dionex IonPac CS16 cation-exchange column (3 × 250 mm, Thermo Fischer Scientific, Waltham, MA, United States) and a Dionex CERS 500 suppressor (2 mm, Thermo Fischer Scientific, Waltham, MA, United States).

For the quantification of extracellular organic acids, a Hitachi Elite Lachrom HPLC (Krefeld, Germany) equipped with an Aminex HPX 87 H column (Biorad, Hercules, CA, United States) as stationary phase and 12 mM H_2_SO_4_ with a constant flow of 0.5 mL min^–1^ as mobile phase was used. The detection was achieved at 210 nm and 45°C with an UV detector (Hitachi, Tokyo, Japan).

### Metabolic Flux Analysis

Calculations of metabolic fluxes in *B. megaterium* were performed in Matlab 7.2 (The Mathworks, Natick, MA, United States) using the open source software OpenFLUX (Quek et al., 2009) and based on the determination of steady state ^13^C labeling of proteinogenic amino acids, substrate uptake, product formation rates and precursor demands for biomass formation. Metabolic reaction network used for simulation was constructed based on previous flux analysis studies and greatly refined using the KEGG and Metacyc databases and genomic data (Fürch et al., 2007; Eppinger et al., 2011). The final model comprises all major central pathways such as glycolysis, pentose phosphate pathway (PPP), tricarboxylic acid (TCA) and anaplerotic reactions but also pathways specific to PHB and proline biosynthesis. Moreover, incorporated precursor demands were corrected using the macromolecular compositions of cells specifically determined for this purpose under each condition (Fürch et al., 2007).

For the determination of mass isotopomer distributions, cells were hydrolyzed (6 M HCl, 105°C, 22 h), lyophilized and submitted for analysis by GC-MS of the tert-butyl-dimethylsilyl-derivatives of the amino acids (Wittmann, 2002; Wittmann et al., 2002). The GC-MS system (Agilent 7890A and MSD 5979C, Agilent Technologies, Waldbronn Germany) was run with 1 mL min^–1^ of helium as carrier gas and equipped with an HP5MS capillary column (5% phenyl-methyl-siloxane diphenypolysiloxane, 30 m × 250 μm) and a triple quadrupole detector. The temperature gradient for separation was set to 120°C for 2 min, 8°C min^–1^ up to 200°C and 10°C min^–1^ until 325°C were reached. Ionization energy was set to 70 eV and inlet, interface and quadrupole temperatures were 250, 280, and 230°C, respectively (Wittmann, 2002). Isotopic steady state was confirmed by measuring amino acid labeling patterns of cells at different cell concentrations (OD_600 nm_ = 2, 4 and 6) and measured ^13^C labeling patterns were automatically corrected for natural isotopes by applying correction matrices (van Winden et al., 2002). Fluxes were finally estimated through minimization of the sum of the weighted least square residuals between measured and simulated mass isotopomer distributions and confidence intervals of 95% for these fluxes were subsequently determined using a Monte-Carlo computational algorithm (Yang et al., 2005; Antoniewicz et al., 2006).

### Characterization of Poly Hydroxy Acids (PHA)

Methanolysis of lyophilized cell dry mass (5 mg) was placed in sealed tubes containing 2 mL methanol, 2 mL chloroform, 15% (v/v) H_2_SO_4_ and 0.5 mg mL^–1^ 3-methylbenzoic acid. The tubes were incubated at 100°C for 4 h. After cooling down to room temperature, 1 mL of ultrapure water was mixed with the reaction solution and vigorously stirred for 1 min with a vortex. The mixture was then transferred to a 15 mL reaction tube and centrifuged for 10 min at 6000 *g*. The lower part of the biphasic solution, containing the methyl esters of the biopolymer, was separated and analyzed via gas chromatography coupled to mass spectrometry (YL6900, Young Instruments, South Korea) using the methodology previously described by Pacheco et al. (2019). Once the retention time of the peaks were contrasted with the standards poly(3-hydrobutyrate) obtained from Sigma-Aldrich, their chemical structures were characterized based on the resulting mass compatibility (NIST 17 Mass Spectral library).

### Intracellular Concentration of Polyhydroxybutyrate (PHB)

Determination of intracellular polyhydroxybutyrate (PHB) content was carried out as described previously (Huang and Reusch, 1996). Briefly, intracellular PHB was first turned into crotonic acid by complete cells hydrolysis with 1 mL of 2 M NaOH (30 min, 99°C) and cell debris were discarded by centrifugation (13200 min^–1^, 5 min, Microcentrifuge 5415R, Eppendorf AG, Hamburg, Germany). Subsequently, supernatants were neutralized with 1 mL of 2 M HCl and crotonic acid concentrations were finally quantified by HPLC measurement using the same system as for organic acids and calibration levels from 50 to 500 mg L^–1^ PHB obtained by hydrolysis of pure PHB granules.

### Field Emission Scanning Electron Microscopy

Field emission scanning electron microscopy of *B. megaterium* cells grown in the absence and presence of 1 M of NaCl were performed as described before (Biedendieck et al., 2007a).

### Statistical Analysis

To spot elements significantly involved in response and adaptation to osmotic stress, transcriptome and proteome data were statistically analyzed with R using packages Venneuler (Wilkinson and Urbanek, 2011) for construction of Venn diagrams and FactoMineR (Lê et al., 2008) for principal component analysis (PCA) analysis and hierarchical clustering on principal components (HCPC). Furthermore, packages gplots (Warnes et al., 2014) and mixOmics (Dejean et al., 2011) were used for heatmap construction and hierarchical clustering on gene from the extended central carbon metabolism using Euclidean distance and complete linkage as measure of distance and dissimilarity, respectively. For flux analysis, the data were visualized using the tool VANTED (Junker et al., 2006) equipped with the FluxMap add-on (Rohn et al., 2012) and obtained flux maps were subsequently refined in Inkskape. Functional analysis of genes and corresponding regulons were inferred from MegaBac v9^1^ and BacillusRegNet (Misirli et al., 2014) databases by comparison with information obtained from literature and the SubtiWiki database for *B. subtilis* (Mäder et al., 2012).

## Results

### Physiological Impact of Ionic Osmotic Stress in *Bacillus megaterium*

To assess the impact of osmotic stress on the physiology of *B. megaterium* strain DSM319, the growth parameters and product yields for organic acids were determined in shaking-flask experiments in minimal medium with various NaCl concentrations ranging from zero to 1.8 M (Table 1). Next, to get a more in-depth insight into the molecular response at the level of the transcriptome, intracellular proteome and metabolome during the exponential growth phase (OD_600 nm_ = 5), microarrays, LC-IMS^E^ and HPLC-measurements were conducted. Obtained data were integrated to deduce general adaptation strategies.

**TABLE 1:** Physiological data for *B. megaterium* DSM319 growing in M9 minimal medium supplemented with different concentrations of NaCl (0, 0.3, 0.6, 0.9, 1.2 and 1.8 M).

**Parameter**	**Unit**	**0 M**	**0.3 M**	**0.6 M**	**0.9 M**	**1.2 M**	**1.8 M**
μ	h^–1^	1.19 ± 0.02	0.88 ± 0.01	0.69 ± 0.01	0.57 ± 0.01	0.39 ± 0.00	0.24 ± 0.00
Y_X/S_	g_CDW_⋅mol^–1^	83.1 ± 1.1	77.2 ± 0.9	74.6 ± 1.1	70.7 ± 1.3	67.3 ± 0.9	58.7 ± 0.7
q_s_	mmol⋅g_CDW_^ –1^⋅h^–1^	14.4 ± 0.3	11.5 ± 0.2	9.3 ± 0.2	8.0 ± 0.2	5.8 ± 0.1	4.0 ± 0.1
Y_Acetate/S_	mmol⋅mol^–1^	**669 ± 21**	526 ± 24	490 ± 19	422 ± 41	346 ± 26	253 ± 22
Y_Pyruvate/S_	mmol⋅mol^–1^	6.4 ± 0.3	7.6 ± 0.4	2.9 ± 0.1	1.7 ± 0.1	2.2 ± 0.2	**20.1 ± 1.6**
Y_Lactate/S_	mmol⋅mol^–1^	4.4 ± 0.3	3.6 ± 0.2	8.3 ± 0.4	15.0 ± 0.6	20.3 ± 0.6	**37.7 ± 2.0**
Y_Succinate/S_	mmol⋅mol^–1^	**62.6 ± 3.2**	22.7 ± 1.0	14.7 ± 0.7	13.4 ± 0.6	10.0 ± 0.4	5.8 ± 0.6
Y_Oxoglutarate/S_	mmol⋅mol^–1^	**9.7 ± 0.4**	2.3 ± 0.1	1.3 ± 0.1	0.7 ± 0.1	1.2 ± 0.1	1.6 ± 0.1
Adenylate energy charge (AEC)		0.8336	n.d.	0.7659	n.d.	0.8612	n.d.
NADH/NAD^+^		0.0079	n.d.	0.0260	n.d.	0.0191	n.d.
NADPH/NADP^+^		0.5162	n.d.	0.4330	n.d.	1.0551	n.d.

*Bold numbers indicate maximal yield observed for each measured organic acids. n.d., not determined.*

A direct correlation between the increasing amount of NaCl and a reduction in the specific growth rate (μ_*max*_) and the resulting biomass yield (Y_X/S_) was observed (Table 1). The cells showed a reduced μ_max_ and Y_X/S_ of around 5-fold and 40%, respectively, at 1.8 M NaCl. This condition also significantly affected the redox state of the cells, provoking an enhanced production of pyruvate (3.1-fold) and lactate (8.6-fold). On the contrary, up to 10.8-fold and 13.4-fold decreased levels of the organic acids, succinate and oxoglutarate, were found (Table 1).

**FIGURE 1:**
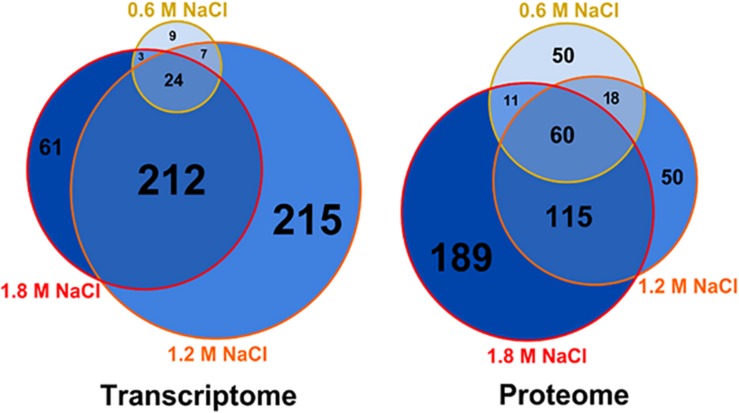
Weighed Venn diagrams of the number of transcripts and proteins whose concentration was significantly altered in *B. megaterium* growing in M9 minimal medium with 0.6, 1.2 and 1.8 M NaCl, respectively. A gene or a transcript was considered significantly regulated when its concentration was either 1.75-fold higher or lower compared to 0 M NaCl. Gene expression was determined by microarray analysis and intracellular proteins were identified and quantified by proteome analysis using LC-IMS^E^.

The combined multi-omic approach performed here demonstrates that the osmotic stress impacts the metabolism of *B. megaterium* far beyond the central carbon metabolism and that gene expression and protein abundances require specific adaptations to deal with these life-threatening conditions (see below). Based on the extent of the alteration of gene expression, a distinction can be made between response to mild (≤0.6 M NaCl) and severe (>0.6 M NaCl) salt stress (Figure 1). Only 43 genes had their expression significantly modified at 0.6 M NaCl, while more than 300 genes were differently expressed at higher NaCl concentration. Among them, 212 genes common to both 1.2 and 1.8 M NaCl were identified. Despite a restricted modification of gene expression, 139 proteins had already significantly altered concentrations under mild salt stress (Figure 1). This number then increased proportionally to the supplemented salt concentration, reaching 375 proteins at 1.8 M NaCl and revealing a large core set of 60 proteins systematically more produced in the presence of salt.

### The Initial Adaptation to High Salt Conditions Is Mediated by Glutamate and Potassium Accumulation

**FIGURE 2:**
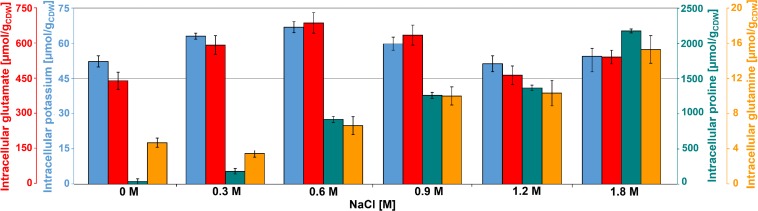
Intracellular concentration of potassium (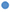), glutamate (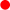), glutamine (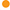), and proline (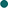) for cultivations of *B. megaterium* DSM319 in M9 minimal medium supplemented with different NaCl concentrations.

**FIGURE 3:**
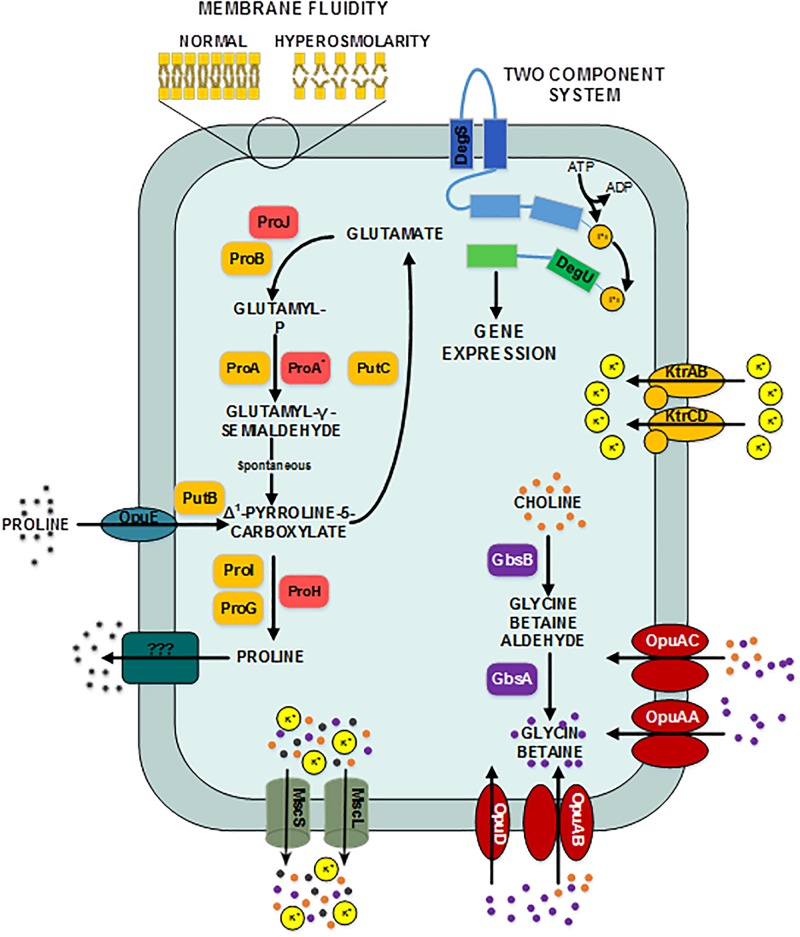
Provisional synthetic overview of the osmotic stress response in *B. megaterium* inferred from genetic context and comparison with *B. subtilis* and *B. licheniformis –* Depending on their function, proteins have been attributed different font colors: light red for synthesis of proline as an osmoprotectant, orange for proline synthesis and utilization for biosynthetic purposes, purple for synthesis of glycin betaine from choline, red for choline and glycin betain transporters, dark turquoise for proline transporters, gray for mechanosensitive channels MscS and mscL, olive green for potassium transporters, dark pine green for unknown proline exporter, dark blue for the input domain of the sensing histidine kinase, light blue for the transmitter domain of the histidine kinase, dark green for the receiver domain of the sensing histidine kinase and light green for the output domain of the response regulator. **DegS:** two-component sensor histidine kinase, **DegU:** two-component response regulator, **GbsA:** glycine betaine-aldehyde dehydrogenase, **GbsB:** choline dehydrogenase, **KtrAB:** high affinity potassium transporter KtrA-KtrB, **KtrCD:** low affinity potassium transporter KtrC-KtrD, **MscL:** large conductance mechanosensitive channel protein, **MscS:** small conductance mechanosensitive channel protein, **ProA:** glutamate-5-semialdehyde dehydrogenase, ProA*: glutamate-5-semialdehyde dehydrogenase **ProB:** glutamate 5-kinase, **ProG:** 1-pyrroline-5-carboxylate dehydrogenase, **ProH:** pyrroline-5-carboxylate reductase, **ProI:** pyrroline-5-carboxylate reductase, **ProJ:** glutamate-5-kinase, **PutB:** proline dehydrogenase, **Opu:** glycine betaine ABC transporter, **OpuAB:** glycine betaine ABC transporter, **OpuD:** glycine betaine transporter.

Glutamate was the most abundant metabolite in *B. megaterium* under standard conditions with a yield of 450 μmol g_CDW_^–1^ (Figure 2). Together with glutamine, it is a central metabolite linking carbon and nitrogen metabolism and a major precursor for the *de novo* synthesis of proline (Figure 3) (Brill et al., 2011a; Gunka and Commichau, 2012). Consequently, the cell must control their intracellular pools very tightly under osmotic stress. While the glutamine pool increased concomitantly with proline titers, the intracellular glutamate pool reached its maximum at 0.6 M NaCl and gradually returned to its initial value at 1.2 M NaCl, matching the profile of intracellular potassium perfectly and revealing the two-sided nature of adaptation to salt stress (Figure 2).

At moderate NaCl-concentrations (≤0.6 M NaCl), cells seem to use glutamate as counterion to imported potassium for an initial adjusting turgor pressure. Its intracellular concentration was accordingly increased (McLaggan et al., 1994; Epstein, 2003). In *B. megaterium*, potassium import could be performed by the conserved uptake systems KtrAB and KtrCD as described for *B. subtilis* (Figure 3). However, the expression of the corresponding genes was not significantly modified (Holtmann et al., 2003). At NaCl concentrations higher than 0.6 M, the increased potassium import was replaced by proline synthesis, probably because higher potassium concentrations are cytotoxic. Accordingly, the glutamate and potassium pools were progressively reduced reaching the initial value (0 M NaCl) at NaCl concentrations of 1.2 M. Responding to the now significantly increased demand for proline, intracellular concentration of glutamate increased again slightly at 1.8 M NaCl (Figure 2). These results suggested that accumulation of potassium plays a central role in short-term response and is also crucial for long-term adaptation of moderate halotolerant bacteria under mild salt stress (Whatmore et al., 1990).

### The Second Adaptation Process to High Salt Conditions Is Based on the Synthesis of the Compatible Solute Proline

Intracellular concentrations of several amino acids, in particular of the compatible solute proline, gradually augmented with medium osmolarity. The cells strongly enhanced their proline production rates with increasing NaCl concentration and it accumulated intracellularly to yields of over 2.2 mmol g_CDW_^–1^ at 1.8 M NaCl (Figure 2 and Supplementary Figure 1). Furthermore, genes involved in the production of glycine betaine showed a 3-fold stronger expression (*gbsA* and *gbsB*). Except for *ousA* 3-fold overexpression, transcription of genes encoding osmoprotectant transporters (*opuAA* to *AC*, *opuD*) was not modified under hypertonic conditions (Supplementary Table 1 and Figure 3) (Steil et al., 2003).

As further highlighted by the hierarchical clustering performed on the expression of genes belonging to the extended central carbon metabolism, rerouting of carbon fluxes toward proline synthesis under osmotic stress was fostered by altered expression of key gene modules. The transcriptome data enabled discrimination between two sets of genes involved in the proline synthesis from glutamate. Although both sets of genes encode the identical three enzymes, namely glutamate-5-kinase (*proB*, *proJ*), glutamate-5-semialdehyde dehydrogenase (*proA*, *proA*^∗^) and pyrroline-5-carboxylate reductase (*proI*, *proH*), their regulation differed significantly. Whereas expression of *proH*-*proJ*-*proA*^∗^ was found 4- and 10-fold enhanced at 0.6 and 1.2 M NaCl, respectively, the expression of *proB*, *proA* and *proI* was up to 3-fold reduced (Supplementary Figure 2). As protein concentrations of ProA and ProA^∗^ behaved in the same way (Supplementary Tables 1, 2), these results suggest that *proHJA*^∗^ encodes the biosynthetic route for the unbridled synthesis of proline as an osmoprotectant, while *proBA* and *proI* seem to be responsible for the anabolic proline production, obviously negatively regulated by the product proline. Under severe osmotic stress, proline biosynthesis was furthermore promoted by the 2- to 5-fold reduced expression of genes from the arginine, amino sugar, purine and pyrimidine metabolisms, which resulted in low levels of the corresponding enzymes and moderated consumption of glutamate and glutamine for these metabolic purposes (Supplementary Figure 2).

Moreover, 2- to 5-fold increased expression of genes encoding the proline transporter OpuE (Bmd_1401) and two enzymes converting proline back to glutamate, the 1-pyrroline-5-carboxylate dehydrogenase (PutC) and proline oxidase (PutB), suggests that cells tightly control their proline intracellular pool and actively recycle this compound to avoid carbon wastage (Figure 3 and Supplementary Table 1). In addition, the concentration of D-amino-acid transaminase (Dat) was increased by 5-fold under hypertonic conditions and certainly improved the supply of the precursor glutamate from alanine (Supplementary Table 2).

### Rearrangement of the Central Metabolism During Osmotic Stress Changes the Redox State of the Cell and Supplies Precursor for Protein Synthesis

To get a better insight into implications of osmotic stress on the central metabolism, we determined the carbon flux distribution by measuring the ^13^C-labeling of proteogenic amino acids from tracer experiments with ^13^C-glucose as substrate in the presence of up to 1.8 M NaCl (Supplementary Tables 3–5). We accounted for condition-specific precursor demands required for flux calculations derived from macromolecular compositions determined at 0, 0.6 and 1.2 M NaCl, respectively. They were subsequently extrapolated from these values for other NaCl concentrations (Supplementary Figure 3 and Supplementary Table 6).

**FIGURE 4:**
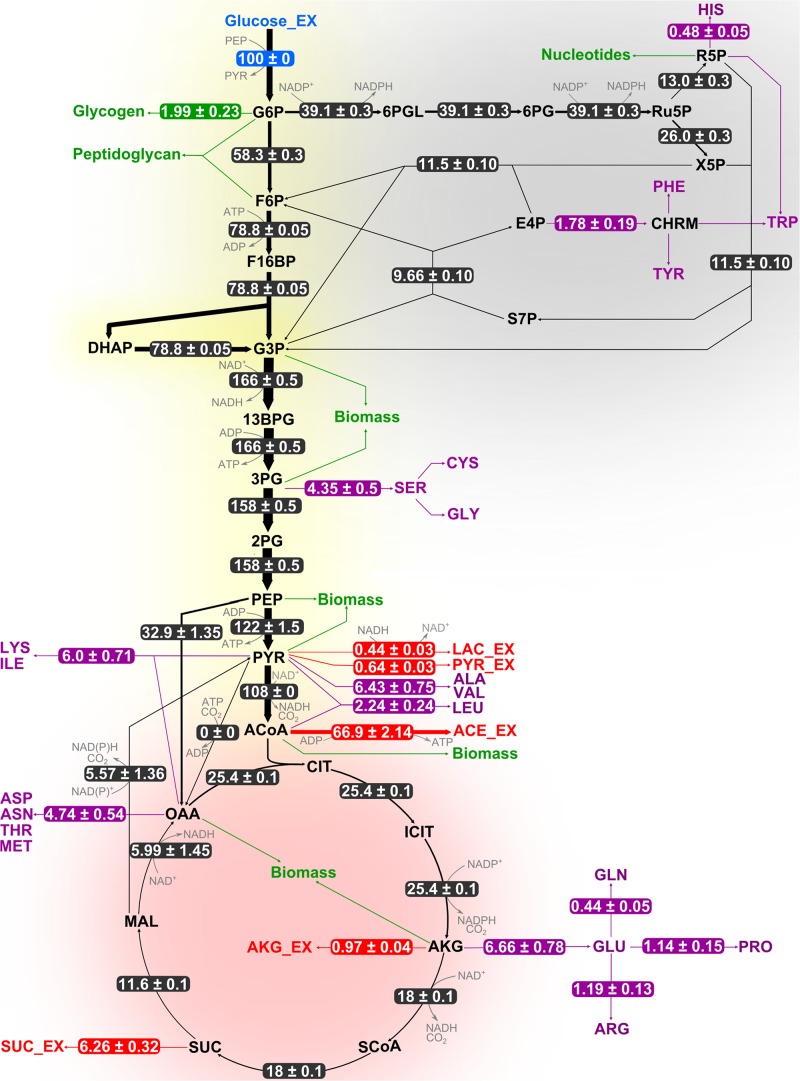
Flux distribution within the central carbon metabolism of *B. megaterium* DSM319 growing at 37°C in M9 minimal medium. Fluxes were determined combining labeling data sets from experiments with 100% 1-^13^C glucose and with a mixture of 50% U-^12^C/50% U-^13^C glucose, respectively. They are given as relative values (%) after normalization with the glucose uptake rate. Fluxes to amino acids (purple) and secretion of organic acids (red) are issued from measurements and were not simulated. Green arrows represent precursor withdrawal for the synthesis of biomass compounds.

**FIGURE 5:**
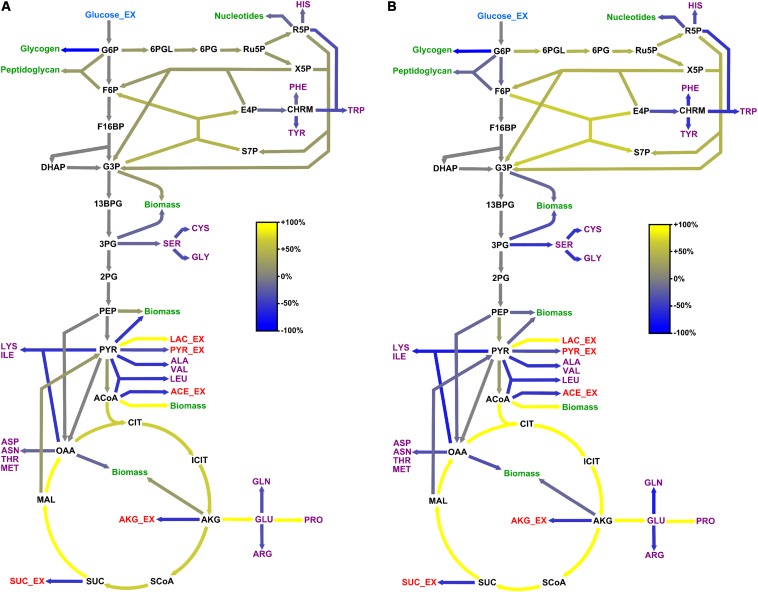
Adaptation of carbon core metabolism to changing osmolarity. Change (%) in relative flux distribution in cells growing with 0.6 M **(A)** and 1.2 M NaCl **(B)** compared to cells growing at 37°C in standard M9 minimal medium (compare Figure 4).

Results from flux analyses showed that *B. megaterium* metabolized glucose using both glycolysis and the pentose phosphate pathway (PPP) under all studied conditions (Figures 4, 5). Besides, phosphoenolpyruvate carboxylase (PepC), an enzyme absent in most other *Bacillus* species, was preferred to pyruvate carboxylase (PycA) for replenishing the tricarboxylic acid (TCA) cycle. However, the coexistence of both enzymes certainly provides *B. megaterium* with enhanced flexibility to cope with a wide range of substrates and environmental conditions as proposed for *Corynebacterium glutamicum* (Sauer and Eikmanns, 2005).

**FIGURE 6:**
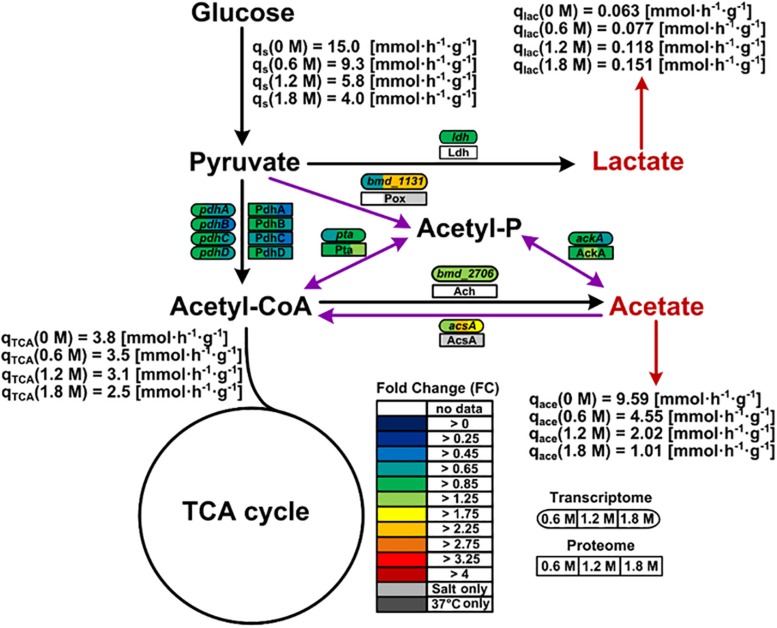
Pox route and overflow metabolism in *B. megaterium* DSM319 growing in M9 minimal medium supplemented with different NaCl concentrations. Purple arrows correspond to reactions of the Pox route while red arrows indicate organic acid secretions. Gene expression was determined by microarray analysis using purified RNA samples obtained from four biological replicates. Intracellular proteins were identified and quantified by proteome analysis using LC-IMS^*E*^ for cells originating from three biological replicates. Values are indicated as fold change compared to expression in cells grown at 37°C in M9 minimal medium without additional NaCl supplementation. Metabolites were quantified by mass spectroscopy. **Ach:** acetyl-CoA hydrolase; **AckA:** acetate kinase; **AcsA:** acetyl-CoA synthetase; **Ldh:** lactate dehydrogenase; **Pdh:** pyruvate dehydrogenase; **Pox:** pyruvate oxidase; **Pta:** phosphate acetyltransferase.

Genes encoding proteins involved in balancing the redox state such as the pyruvate oxidase Pox (*bmd_1131*) were found overexpressed (2.5-fold), correlating well with similar observations made for the proteome of cells exposed to at least 1.2 M NaCl (Figure 6). Hence, the Pox enzyme might participate in the reduction of the NADH-to-NAD^+^ ratio by circumventing the utilization of NAD^+^ dependent pyruvate dehydrogenase Pdh for the conversion of pyruvate to acetyl-CoA whose concentration indeed tended to diminish at the mRNA and protein levels under high salt condition (Figure 6).

Since the *pox* gene showed no difference in expression at 0.6 M NaCl despite the already increased NADH-to-NAD^+^ ratio, activation of the Pox route seems only indirectly related to the cellular redox state. Coming from the Pox route, the main pathway affected by severe osmotic stress in *B. megaterium* involves the TCA cycle. The expression of the gene encoding acetyl-CoA synthase (*acsA*) displayed a 2.6-fold increase, most likely to ensure efficient redirection of the produced acetate toward the TCA cycle (Figure 6 and Supplementary Table 1) (Li et al., 2006; Kohlstedt et al., 2014). It was shown that *B. megaterium* accumulates extracellularly acetate by secretion during cultivations without re-import (Hollmann and Deckwer, 2004). Hence, it seems again that cells tend to avoid carbon wastage through secretion and instead convert acetate into acetyl-CoA to meet the physiological constraints imposed by osmotic stress.

Comparison of the flux distributions in non-stressed cells with that in cells exposed to mild (≤ 0.6 M NaCl) and cells exposed to severe osmotic stress (>0.6 M NaCl), respectively, revealed a intensification of relative fluxes through the TCA cycle with increasing salt concentration (Figure 5). The absolute flux slightly decreased (Figure 6). Further, a rerouting of carbon from 2-oxoglutarate toward glutamate and proline synthesis was observed (Figure 5). Moreover, the precursor drain for the synthesis of biomass compounds, organic and amino acids upstream and downstream from the 2-oxoglutarate node significantly decreased with increasing salt concentration. In particular, carbon fueling the TCA cycle was preferentially recycled to oxaloacetate by malate dehydrogenase and reincorporated into the cycle, explaining the observed flux repartition at the anaplerotic node. Overall, the fluxes around this node showed no alteration despite the increased utilization of 2-oxoglutarate for proline biosynthesis.

Similarly, relative flux through the PPP intensified proportionally to the imposed osmotic burden and exceeded by far the anabolic demand, resulting in a strengthened carbon feeding back to glycolysis intermediates. Since three moles of NADPH are necessary to synthesize one mole of proline from 2-oxoglutarate, this marked increase of PPP fluxes certainly provided cells with the reducing power required for this conversion. Together with the reduced CO_2_ fixation by phosphoenolpyruvate carboxylase, these combined increases of fluxes through the TCA cycle and PPP also resulted in an up to 70% stronger CO_2_ release, which partly explains the observed reduction of biomass yield under osmotic stress (Table 1 and Figure 5). In contrast, relative fluxes through glycolysis remained approximately constant and admittedly participated in maintaining a high energy level even under stressful conditions, as indicated by the measured adenylate energy charge (Table 1 and Figure 5).

**FIGURE 7:**
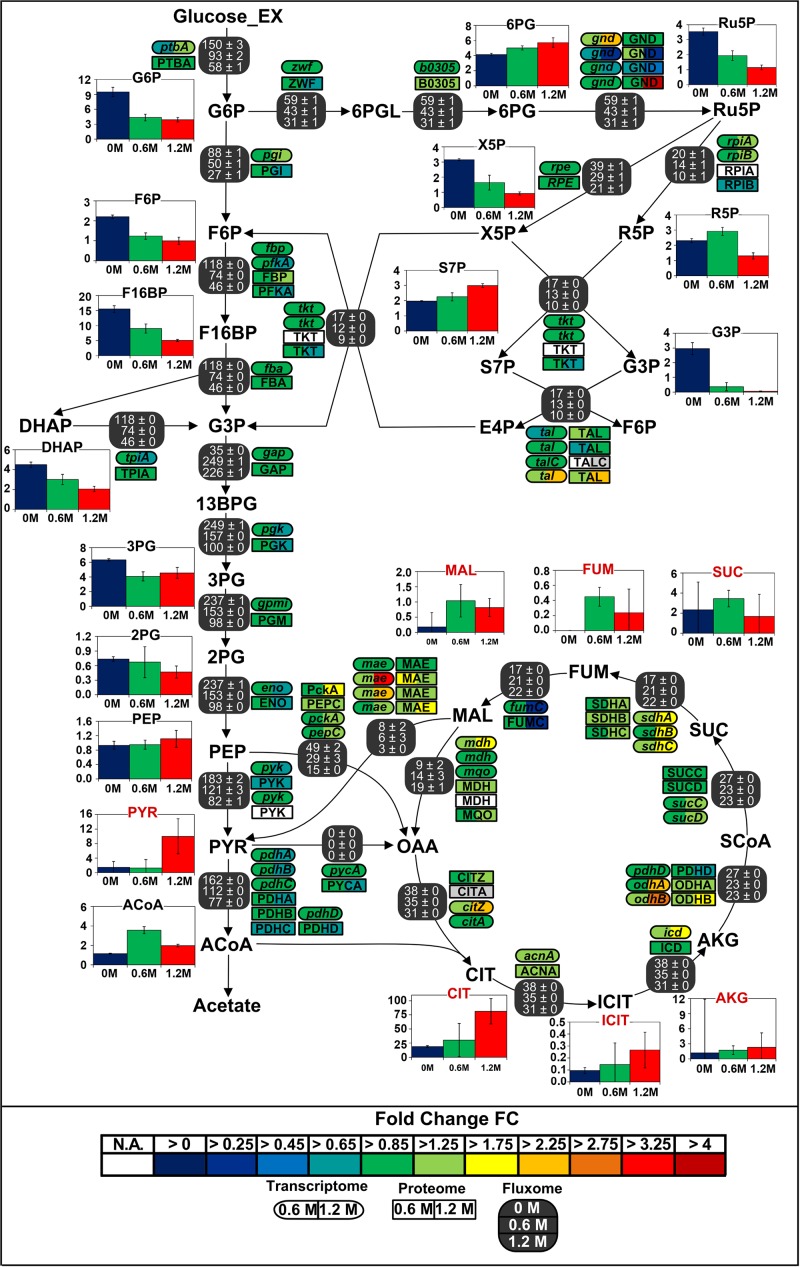
Integrated view of the response of the central carbon metabolism of *B. megaterium* DSM319 to ionic osmotic stress. Transcriptome and proteome data are indicated as the determined fold change compared to cultivation in minimal medium without NaCl supplementation. Gene expression was determined by microarray analysis using purified RNA samples obtained from four biological replicates. Intracellular proteins were identified and quantified by proteome analysis using LC-IMS^*E*^ for cells originating from three replicates. Bar plots represent intracellular metabolite concentrations in μmol g_CDW_^–1^. Intracellular metabolite concentrations were determined by LC-MS/MS using a differential method, i.e., subtracting extracellular metabolite concentration from the global metabolite concentration.

Regarding the large carbon flux from glycolysis to proline biosynthesis, expression of several genes belonging to the TCA cycle and anaplerotic node was up to 3-fold higher in stressed cells (Figure 6). Concentrations of corresponding proteins were only slightly higher and cannot account alone for the massive rerouting observed, mainly because salt could also induce loss of enzyme activity (Kohlstedt et al., 2014). On the contrary, despite a 2.6-fold reduction of absolute glycolytic flux, the pools from oxaloacetic acid to 2-oxoglutarate grew more prominent with increasing salt concentration and indeed drove this rerouting which enabled the conservation of a similar TCA absolute flux (Figure 7). Similarly, mRNA and protein levels from the PPP did not significantly change under osmotic stress, and enhanced flux diversion may be achieved at the level of metabolites as well. As a matter fact, concentration of 6-phosphoglycerate (6PG) also increased gradually while that of ribulose-5-phosphate (Ru5P) progressively diminished with increasing salt concentration, thus reducing the mass-action ratio of the reaction catalyzed by phosphogluconate dehydrogenase (GND) and favoring 6PG conversion (Figure 7) (Hess and Brand, 1965).

Gene expression, enzyme concentrations and metabolic fluxes downstream of 2-oxoglutarate were not significantly changed under osmotic stress in *B. megaterium* except for the *odhA* and *odhB* genes, whose expression was higher (Figure 7 and Supplementary Tables 1, 2) while *fumC* and the corresponding enzyme fumarate hydratase (FumC) were found to be up to 3-fold reduced at severe salt. Consistent with results in *B. subtilis* and *B. licheniformis*, expression of genes encoding glutamate synthase (*gltA*, *gltB*) and concentration of these proteins were also up to 2-fold reduced despite the increased glutamate demand for proline synthesis (Schroeter et al., 2013). These three enzymes seem stress-sensitive and, given their vital metabolic functions, they are probably replaced by isoenzymes under stressful conditions.

### Induction of the General *sigB*-Mediated Stress Response by High Salt Conditions

**FIGURE 8:**
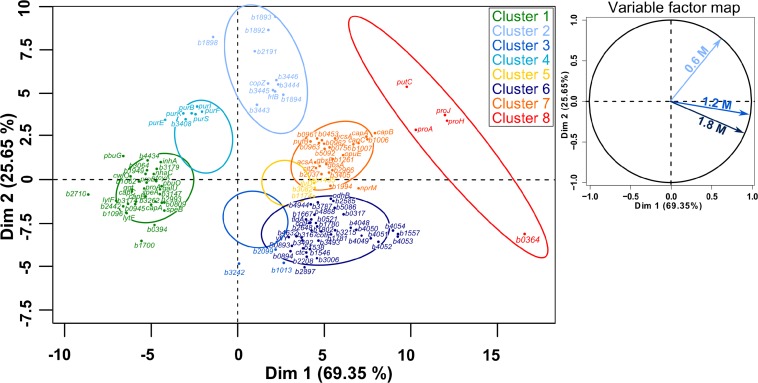
Principal component analysis (PCA) followed by hierarchical clustering (HCPC) on gene expression ratios in *B. megaterium* DSM319 grown in M9 medium supplemented with 0.6, 1.2, and 1.8 M NaCl. For more clarity, *bmd* was replaced by b in gene names and only the 125 genes most relevant for the PCA construction are presented. **Cluster 1**: Genes whose expression is down-regulated under both mild (0.6 M NaCl) and severe salt stress (1.2 and 1.8 M NaCl), **Cluster 2**: Gene whose expression is only stronger under mild stress, **Cluster 3**: Genes whose expression is slightly reduced at 0.6 M NaCl and slightly increased under severe salt stress, **Cluster 4**: Genes whose expression is slightly up-regulated at 0.6 M NaCl and strongly down-regulated under severe salt stress, **Cluster 5**: Genes whose expression is slightly up-regulated under both mild and severe salt stress, **Cluster 6**: Genes whose expression is specifically up-regulated under severe salt stress, **Cluster 7**: Genes whose expression is up-regulated under both mild and severe salt stress, **Cluster 8**: Genes involved in proline synthesis.

When cells were cultured at NaCl concentrations higher than 0.6 M, expression of the σ^*B*^-operon (*rsbV*, *rsbW*, *rsbX*, *sigB*) was only up to 1.8-fold higher, while the abundance of several of its products increased up to 3-fold, suggesting the sustained activation of the general stress response under acute salt stress. In accordance with this conclusion, predicted members of the SigB-regulon (*bmd_1994*, *bmd_1557*, *bmd_1546*, *bmd_1131*, *bmd_1041*, *dps*, *bmd_5086*, *bmd_3493*, *bmd_3215, gbsB, gbsA*) were between 2.2- and 12-fold more strongly expressed and concentration of the corresponding proteins up to 57-fold higher (Supplementary Figure 4 and Supplementary Tables 1, 2) (Hecker and Völker, 2001; Höper et al., 2006; Hahne et al., 2010; Misirli et al., 2014). Similarly, the concentration of the regulator of the peroxide regulon PerR was 2.6- and 4.6-fold increased at 1.2 M and 1.8 M NaCl, respectively, confirming the production of reactive oxygen species (ROS) under these conditions. The increased production of NADH dehydrogenase YutJ (BMD_4957), iron-binding protein Dps (BMD_4857), 2-cys peroxiredoxin (BMD_0990), redox regulator Rex (BMD_0255), several cytochromes P450 (BMD_1855, BMD_2035, BMD_3874) as well as the up to 4-fold induction of the gene encoding a manganese catalase (*bmd_3215*) provides further evidence that the cells produced these proteins to fight oxidative damages (Figure 8 – Cluster 6, Supplementary Figure 4 and Supplementary Table 2) (Lewis, 2002; Ishikawa et al., 2003; Rhee et al., 2005; Gyan et al., 2006; Ying, 2008).

Interestingly, concentrations of several flavodoxins (BMD_3384, BMD_3385, BMD_3911) were increased up to 6.6-fold at concentrations above 0.6 M NaCl and electron transfer flavoproteins EtfA and EtfB were even produced explicitly under these conditions. Flavodoxins have already been reported to replace ferredoxins and participate in repair activities during iron starvation and oxidative stress (Pomposiello et al., 2001; Giro et al., 2006; Tognetti et al., 2006; Zurbriggen et al., 2007). Indeed, ferredoxin functions seem to be compromised under stressful conditions because the Fe–S cluster they bear as a prosthetic group gets damaged by diverse reactive species (Singh et al., 2014). In this regard, expression of genes involved in synthesis and reparation of Fe-S clusters (*sufB*, *iscU*, *sufS*, *sufD*, *sufC*) showed upregulation by around 2-fold and concentrations of their products were accordingly higher under severe salt stress (Supplementary Tables 1, 2) (Höper et al., 2006). Surprisingly despite the catalytic role of iron in ROS generation, transcription of numerous genes encoding proteins involved in iron acquisition such as siderophores (*bmd_4048*, *bmd_4051*, *bmd_4052*) and ferrichromes (*fhuD*, *fhuC*, *yclQ*, *yclP*, *yclO*, *yclN*, *yusV*, *yfhA*, *yfiZ*, *yfiY*) were among the most overexpressed (up to 15-fold) and their products display an up to 12-fold abundance increase at concentrations above 0.6 M NaCl (Figure 8, Cluster 6 and Supplementary Figure 4) (Galaris and Pantopoulos, 2008). Together with the increased production of flavodoxins, this result tends to confirm that high-salinity is also causing iron scavenging in *B. megaterium*, as proposed in *B. subtilis* (Hoffmann et al., 2002; Höper et al., 2006; Zurbriggen et al., 2007).

### Additional Adaptation Process Caused by High Salt Conditions

Proteome data also revealed a progressive reduction of concentrations of several ribosomal proteins (RplW, RpsN, RpsO, RpsT, RpmA, RplT), translation factors (BipA, PrfC, PrfB) and proteins from the purine and pyrimidine metabolisms (PurEKBCSQLFMNHD, PyrK, PyrAA, PyrAB, PyrC, PyrB, PyrG) with increasing salt concentrations which denotes a significant downturn in RNA synthesis and processing activities consistent with the reduction of RNA content (Supplementary Figure 3 and Supplementary Table 1). In this regard, decrease in concentrations of enzymes involved in the synthesis of purines and pyrimidines was supported by the up to a 2.5-fold reduced expression of genes encoding them and proteins from the connected histidine biosynthesis pathway (Figure 8 – Cluster 4).

Besides proline, arginine and histidine metabolisms, synthesis and transport of several other amino acids were affected at NaCl concentrations above 0.6 M. Notably, genes encoding methionine and cystine ABC transporters (*met*, *metN*, *metQ*, *metP*, *tcyC*, *tcyB*, *tcyA*) or involved in methionine salvage (*mtnA*, *mtnK*, *mtnE*, *mtnW*, *mtnX*, *mtnB*, *mtnD*) had approximately 2-fold decreased expression levels while concentration of methionine synthase MetE and cystathionine beta-lyase PatB was up to 16.5- and 2.7-fold increased, respectively (Supplementary Tables 1, 2). Similarly, concentration of enzymes involved in tryptophan (BMD_2992, TrpA, TrpB, TrpC) and cysteine (YtkP) synthesis were up to 2.5- and 4-fold increased under severe salt stress, respectively. Given the reactivity of reactive oxygen and nitrogen species toward methionine, cysteine and tryptophan residues, all these modifications might be related to the emergence of oxidative damages under severe salt stress (Levine et al., 1999; Yamakura and Ikeda, 2006; Hochgräfe et al., 2007; Peyrot and Ducrocq, 2008). The up to 5-fold higher concentrations of enzymes from the pantothenate pathway (PanB, PanC, PanD) could help prevent oxidative damages (Supplementary Figure 4 and Supplementary Table 2) (Wojtczak and Slyshenkov, 2003).

**FIGURE 9:**
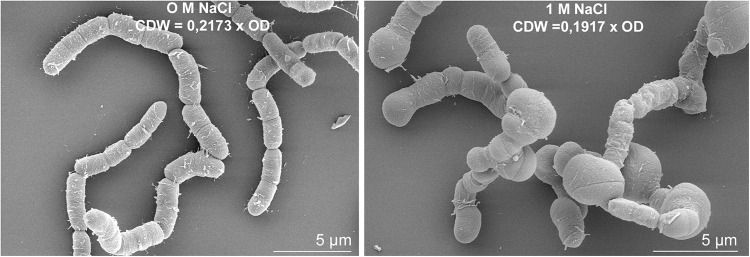
Salt-induced modifications of cell morphology in *B. megaterium* DSM319. Scanning electron microscope picture of *B. megaterium* grown in the absence **(left)** and in the presence of 1 M NaCl **(right)**.

The expression of several genes encoding proteins associated to the cell wall or involved in peptidoglycan, murein and polysaccharide synthesis (*bmd_0452*, *bmd_1096*, *ponA*, *cwlO*, *yocH*, *lytF*, *lytE*, *bmd_1114*, *bmd_1117* to *1120*, *bmd_3174*) was up to 5-fold reduced at concentration above 0.6 M NaCl. Most of the corresponding proteins also had reduced concentrations, with the exception of the binding protein YocH which was, despite the 3-fold reduction in abundance, present in 12- and 19-fold higher amounts at 1.2 and 1.8 M NaCl, respectively (Supplementary Figure 4 and Supplementary Tables 1, 2) (Steil et al., 2003; Kohlstedt et al., 2014). Another essential element determining cell morphology in bacteria is the cell division protein FtsZ (Chien et al., 2012), whose abundance was enhanced by more than 3-fold (Supplementary Table 2). The repression of this gene leads to longer cells displaying a filamentous shape (Margolin, 2005), and in contrast by overproducing FtsZ, a higher growth rate and wider bacterial cells (Zhao et al., 2019). Figure 9 shows the morphology of *B. megaterium* cells, which swelled in the presence of sodium chloride. The cell wall indeed underwent an electrostatic contraction affecting its structure and properties as described by others (Figure 9) (Marquis, 1968; Koch, 1984). Moreover, the percentage of odd-numbered iso-fatty acids (iso C13:0 and iso C15:0) incorporated into the cell wall was gradually increased in cells exposed to higher NaCl-concentrations and probably responded to a reduction of membrane fluidity at higher osmolarities (data not shown) (Kaneda, 1991).

The PCA analyses further confirmed the existence of a physiological threshold at around 0.6 M NaCl and enabled the detection of four genes encoding unique two-component systems (*bmd_1892/1893* and *bmd_3442/3443*) which responded specifically to mild salt stress and might orchestrate an appropriate feedback response (Figure 8 – Cluster 2). In particular, an elevated transcription of *copZ*, *copA*, and *bmd_1894*, whose product shares 40% homology with *B. subtilis*’ CsoR regulator, may indicate an intensified scavenging of intracellular copper under these conditions (Banci et al., 2003; Radford et al., 2003; Corbett et al., 2011). Other induced genes encode an ABC transporter (*bmd_3446*) and two putative membrane proteins (*bmd_3444* and *3445*). Nevertheless, 55% of the 43 genes differently expressed under mild salt stress were shared with severe salt stress, and their regulation seems therefore crucial for adaptation. This core group naturally comprises genes with functions in proline synthesis (*putC*, *proH*, *proJ*, *proA*^∗^, see above) whose expression was up to 10.4-fold increased (Figure 8 – Cluster 8) but also others involved in cell wall metabolism (*lytF*, *bmd_2442*, *bmd_1096*, *bmd_3174*) which were, on the contrary, up to 4.4-fold less expressed under osmotic stress (Figure 8 – Cluster 1) (Steil et al., 2003). Besides, several NAD-dependent epimerases/hydratases (Bmd_0685, GalE, Mro, Bmd_2433, Bmd_2930 Bmd_3943) were found in response upon addition of NaCl whose concentrations increased up to 29-fold (Supplementary Figure 4 and Supplementary Table 2). Finally, several oxidoreductases (BMD_0912, BMD_0989, BMD_1041, BMD_2681, BMD_3119, BMD_3139, BMD_3288, BMD_3473, BMD_3493), peptidases and proteases (BMD_0331, InhA, BMD_3039, PepQ, BMD_4817, CtpB, BMD_5202) were also part of this core group of proteins and their increased concentrations positively contributed to a reduction of damages resulting from the salt-induced perturbation of redox state and to the alteration of cell wall (Supplementary Figure 4 and Supplementary Table 2) (Wang et al., 2006).

### Osmotic Stress Triggers Polyhydroxybutyrate (PHB) Synthesis in *B. megaterium*

**FIGURE 10:**
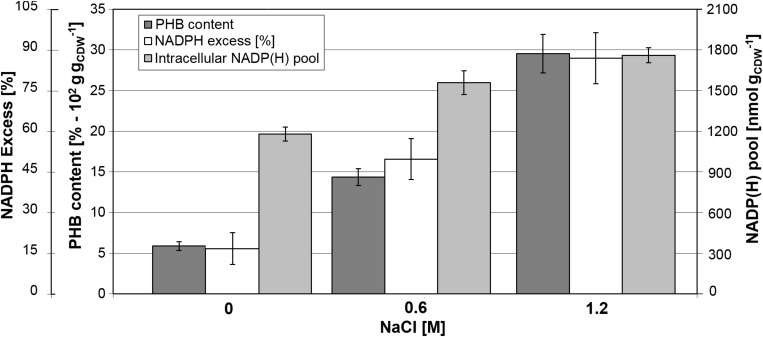
Relationship between PHB content and NADPH production in *B. megaterium* DSM319 growing at 37°C in M9 minimal medium supplemented with up to 1.2 M NaCl. NADPH excess was calculated using the ratio between NADPH supply derived from flux analysis and biosynthetic NADPH demand estimated from biomass composition at the respective NaCl concentrations. Global NADP(H) pool represents the sum of NADP and NADPH pools as.

Determination of biomass composition under different NaCl concentrations revealed elevated levels of intracellular inclusion bodies under hypertonic conditions, most likely as a response mechanism to osmotic stress. Using gas chromatography mass spectrometry, we found that *B. megaterium* accumulated poly(3-hydroxybutyrate) (PHB). Under standard growth conditions, the PHB content was recorded at 6% of the CDW but increased to 14.4% at 0.6 M NaCl, and finally reached an accumulation of 29.5% of the CDW when cells grew with 1.2 M NaCl (Figure 10). As evidenced by fluxome analysis, a side effect of proline synthesis under salt stress was a stronger NADPH supply which was reflected in the increase of the global NADP(H) pool from 1180 to 1760 nmol g_CDW_^–1^ in cells cultivated with 1.2 M NaCl (Figure 10). However, NADPH supply already exceeded biosynthetic demand by more than 20% under normal conditions, and the increased PPP fluxes under salt stress only accentuated this discrepancy, generating an 87% NADPH excess at 1.2 M NaCl. The PHB biosynthetic pathway requires NADPH as a cofactor; thus, an increment in the NADPH pool has been reported to enhance the intracellular accumulation of the polyoxoester, while PHB synthesis might act as a redox regulator of the NADPH-to-NADP^+^ ratio. Similarly, increased conversion of pyruvate to lactate is probably involved in the modulation of the NADH-to-NAD^+^ ratio (Anderson and Dawes, 1990; Wang et al., 2005). Despite the higher PHB content at elevated salt concentrations, expression of genes and abundance of proteins involved in PHB synthesis showed no alteration in cells grown with 0.6 M NaCl. *B. megaterium* cells exposed to 1.2 M NaCl prompted the synthesis of the PhaR subunit of the polyhydroxyalkanoates (PHA) synthase and the phasin PhaP enzyme. The latter, a known stimulator of PHB production, showed a 2.2- and 1.8-fold increase at 0.6 and 1.2 M NaCl, respectively. These slight modifications cannot account alone for the sharply increased polymer content (York et al., 2001; Pötter and Steinbüchel, 2005) and suggest that other unknown underlying mechanisms trigger PHB accumulation when osmotic pressure becomes higher in *B. megaterium.*

## Discussion

As a soil bacterium, *B. megaterium* faces constant fluctuations of nutrient and water availability as a result of seasonal and daily weather variations. In this study, we aimed at unveiling the metabolic responses and regulatory mechanisms at the transcriptomic and proteomic level coping with the osmotic stress conditions (Schweder et al., 1999). In addition, metabolomics and *in vivo* flux analysis were integrated with the other molecular levels, thus delivering a more comprehensive and holistic explanation of the resulting phenotypes observed in *B. megaterium* cells challenged with hyperosmotic stress.

Firstly, at similar NaCl-concentrations, detrimental consequences on physiology were less pronounced in comparison to other members of this genus (den Besten et al., 2009; Schroeter et al., 2013; Kohlstedt et al., 2014). This higher robustness of *B. megaterium* was further reflected in the up to 5-fold reduction of the number of genes whose expression was significantly altered at 1.2 M NaCl compared to *B. cereus, B. subtilis*, or *B. licheniformis* (den Besten et al., 2009; Hahne et al., 2010; Schroeter et al., 2013). With increasing NaCl amounts, the intracellular proline concentration drastically rose, reaching its maximum at 1.8 M NaCl. Since *B. megaterium* does not possess the genetic machinery for the *de novo* synthesis of other osmoprotectants such as ectoine, glycine betaine or trehalose, it seems that proline acts as the major osmoprotectant in this organism. Higher levels of intracellular proline were related to the upregulation of the expression of the genes *proJ-proA^∗^-proH* encoding glutamate-5-kinase, glutamate-5-semialdehyde dehydrogenase and pyrroline-5-carboxylate reductase, respectively. The genes *proB*, *proA*, and *proI*, encoding the same proteins showed low expression under high salinity, obviously negatively influenced by proline, as previously reported in *B. subtilis* and *B. licheniformis* (Grundy and Henkin, 1993; Brill et al., 2011a, b). The existence of two distinctive ProA proteins indicates a strict separation between proline synthesis for anabolic and protective purposes similar to that in *B. licheniformis* and contrasting with the organization in *B. subtilis*, in which both routes are curiously interlinked by the unique ProA (BSU13130) (Brill et al., 2011a; Schroeter et al., 2013). Additionally, the upregulation of genes and proteins responsible for the last steps of the glycine betaine biosynthesis under high salt conditions suggest that, as observed in *B. subtilis*, synthesis of this compatible solute is only possible if its precursor choline is also present (Boch et al., 1996; Kappes et al., 1999; Nau-Wagner et al., 2012).

Another essential metabolic regulation occurs at the level of pyruvate where conversion to acetyl-CoA is modulated to fine-tune fluxes through the TCA cycle. In that case, the extra NADH supply resulting from the stronger production of malate dehydrogenase and 2-oxoglutarate oxidoreductase enzymes under salt stress has to be dissipated, and *B. megaterium* thus secretes higher amounts of lactate depleting NADH as cofactor. Under osmotic stress conditions, it seems clear that the pool of acetyl-CoA goes to the TCA cycle for 2-oxoglutarate synthesis, one of the main precursors for proline synthesis. The high operation of enzymes of the TCA cycle to process the carbon flux from acetyl-CoA, indeed, lead to an extreme synthesis of NADH. Hence, *B. megaterium* might partially release this burden by redirecting two molecules of acetyl-CoA to acetoacetyl-CoA (by B-ketothiolase PhaA), and subsequently to PHB synthesis (Madison and Huisman, 1999). This pathway also utilizes NADPH as cofactor, allowing balancing the entire redox state of the cell.

This enhanced intracellular accumulation of polyhydroxybutyrate (PHB) when NaCl concentration increases, surprisingly under non-limiting conditions of nutrients, was one of the most significant findings of this study. In fact, PHB is generally produced in bacteria when an inorganic nutrient, other than the C-source, becomes limiting, enabling the intracellular storage of excess carbon and reducing power under these conditions (Rehm, 2010). Under famine, the cell can then degrade the accumulated PHB to acetyl-CoA and (S)-3-hydroxybutyl-CoA, replenishing the TCA and β-oxidation cycles for energy production, respectively (Wang et al., 2009). Beneficial effects of PHB and related phasins on bacterial resistance against stressors such as heavy metals, temperature, phenol, ethanol, and peroxide stress have already been underlined in several studies, but a potential role against salt stress has only been advanced in nitrogen-fixing rhizobia so far (Natarajan et al., 1995; Ayub et al., 2004; Arora et al., 2006; Zhao et al., 2007; Nair et al., 2009; Wang et al., 2009; Woo et al., 2012). System-wide integration of all omics data explains this PHB build-up in *B. megaterium*. Indeed, increased mRNA and protein levels were observed within the PPP and TCA cycle under ionic osmotic stress as compared to the control condition with no salt. Consequently, the carbon flux toward the former pathway increased and generated an NADPH surplus in cells grown in glucose and high NaCl concentrations. These findings were also confirmed by the *in vivo* flux analysis. This diversion of carbon flux toward the PPP pathway was furthermore fostered by a decreased concentration of the phosphofructokinase (PfkA) with increased NaCl concentrations that reduced efficiency of the upper part of the glycolysis. This cellular strategy has been proven to endow bacteria with the ability to cope with oxidative stress and guaranty production of cofactors for anabolic demands (Chavarria et al., 2013). However, in the case of *B. megaterium*, it also leads to an intracellular excess of NADPH and the observed accumulation of PHB.

In most PHB producing bacteria, nitrogen, or phosphorus are limited during the bioconversion of the supplied carbon source to trigger biopolymer synthesis in the cell (Mozejko-Ciesielska and Kiewisz, 2016). This nutrient-limited environment suppresses key enzymes of the TCA cycle such as the isocitrate dehydrogenase followed by the downregulation of genes encoding the succinate and malate dehydrogenase (Klonne et al., 1989; Poblete-Castro et al., 2012). Findings here were contrary to the usual mechanisms displayed by environmental bacteria for synthesizing polyhydroxyalkanoates (PHA), opening new avenues for the synthesis of this valuable biopolymer using varying salt concentrations under non-limiting nutrient conditions. The use of fatty acids as carbon substrates allows PHA synthesis in a growth-associated manner as demonstrated in *Bacillus* species (Sangkharak and Prasertsan, 2012), *Cupriavidus necator* (Verlinden et al., 2011), *and Pseudomonas putida* (Oliva-Arancibia et al., 2017) to name some, because of the generation of direct precursors, e.g., (*S*)-3-hydroxyacyl-CoA and 2-enoyl-CoA, from the B-oxidation route. One of only a few bacteria that does not require any nutrient limitation for PHA storage from glucose is *Azotobacter vinelandii*, a soil bacterium capable of nitrogen fixation (Hamilton et al., 2011). The biopolymer has been proposed to act as a protective agent to counteract oxidative stress during carbon deprivation as a result of the constant functioning of the nitrogenase enzyme (Page et al., 1992).

In the last decade, bacteria of the genus *Bacillus* have been exploited as efficient PHA producers using a wide variety of carbon substrates and wastes (Mohapatra et al., 2017) as well as a host for the creation of engineered and functionalized PHA beads (Grage et al., 2017). Halotolerant *Bacillus* strains are being utilized to develop unsterile bioprocesses for high-level PHB synthesis since they can rapidly grow with a NaCl concentration up to 2 M and accumulate approximately 50% of the CDW as PHB (Page et al., 1992). Fed-batch PHA processes using *Bacillus* species have already reached volumetric productivities similar to those obtained at industrial scale (Kulpreecha et al., 2009). Also, the biosynthesized polyoxoesters contain fewer endotoxins in comparison to Gram-negative bacteria, offering an advantage for medical applications. Based on the metabolic functioning of *B. megaterium* unveiled in this study, there is a lot of room for improvement for PHB production since the genes of the biopolymer biosynthetic pathway were almost not altered under hyperosmotic conditions. Genetic engineering of this route could enable a higher conversion of precursors of acetyl-CoA to acetoacetyl-CoA. Moreover, these genetic modifications toward the creation of novel biocatalysts can be coupled with unsterile bioprocessing development, making a cost-effective process for bacterial production of biopolymers from sugars.

## Conclusion

Adaptation to ionic osmotic stress in *B. megaterium* was found to be initiated by the accumulation of synthesized glutamate and imported potassium within the cell. This step was followed by the massive *de novo* synthesis of the compatible solute proline and recruited an osmo-dependent pathway to fulfill this requirement. Here, higher mRNA and protein levels within these pathways were found and confirmed the reorganization of flux distribution toward proline production. Moreover, relative fluxes through the pentose phosphate pathway and tricarboxylic acid cycle were significantly increased to supply the cofactors NADPH and NADH, respectively. As a consequence, NADPH was present in significant excess in cells under sustained osmotic stress, triggering an accumulation of the storage compound PHB, a highly promising industrial biopolymer.

## Data Availability Statement

The datasets generated for this study can be found in the GEO database with the accession number GSE110712; ProteomeXchange Consortium with the dataset identifier PXD015605.

## Author Contributions

TG, RK, and RB conceived the idea. TG, DZ, GR, MW, and MR performed the experiments. TG, DZ, KR, IP-C, GR, RK, and RB analyzed the data. TG, IP-C, RK, and RB wrote the manuscript getting the input of all authors. All authors edited the manuscript and approved its final version.

## Conflict of Interest

The authors declare that the research was conducted in the absence of any commercial or financial relationships that could be construed as a potential conflict of interest.

## Footnotes

1 http://www.megabac.tu-bs.de

## Abbreviations

6PG: 6-phosphoglycerate
CDW: cell dry weight
CoA: Coenzyme A
HCPC: hierarchical clustering on principal components
GC-MS: gas chromatography/mass spectrometry
HPLC: high performance liquid chromatography
LC-IMS: liquid chromatography ion mobility spectrometry
MS: mass spectrometry/spectrometer
PCA: principal component analysis
PHA: polyhydroxyalkanoate
PHB: polyhydroxybutyrate
PPP: pentose phosphate pathway
ROS: reactive oxygen species
Ru5P: ribulose-5-phosphate
UPLC: ultra-high performance liquid chromatography.
