# Supplementary material for: Metabolic Rearrangements Causing Elevated Proline and Polyhydroxybutyrate Accumulation During the Osmotic Adaptation Response of Bacillus megaterium
Source: Front Bioeng Biotechnol. 2020 Feb 21;8:47. doi: 10.3389/fbioe.2020.00047 (PMC7053513; doi:10.3389/fbioe.2020.00047)
Supplement: Supplementary file 1 [file Table_1.DOC]

***Supplementary Material***

1. **Supplementary Data**

**Bacterial strain and growth conditions**

A glycerol stock of *B. megaterium* cells was used as inoculum for pre-cultures and incubated in shake flasks with M9 minimal medium until their optical density (OD_600nm_) reached 1.5 (Multitron II, 50 mm shaking diameter, 230 min^-1^, Infors HT, Böttmingen, Switzerland). Cells were harvested, washed and used to inoculate main cultures to an OD_600nm_ of 0.1. Main cultures were incubated in 50 mL of M9 minimal medium in shake flasks (Multitron II, 50 mm shaking diameter, 230 min^-1^, Infors HT, Böttmingen, Switzerland).

For PHB production, batch reactor cultivations were performed in a parallelized 1 L-bioreactor system (DASGIP®, Jülich, Germany) with a working volume of 700 mL. To this end, cells from shake flask cultures were collected at an OD_600nm_ of 4 and further used as inoculum for shake flasks containing 100 mL of M9 minimal medium (OD_600nm_ = 0.1). Once OD_600nm_ of these cultures reached 4, cells were harvested, washed and used to inoculate bioreactors to an initial OD_600nm_ of 0.1. Reactor stirrer speed and aeration rate were set at 400 min^-1^ and 6 L h^-1^, respectively, and progressively increased during cultivation to maintain dissolved oxygen concentration at around 30 % saturation (Visiferm DO, Hamilton Messtechnik GmbH, Höchst, Germany). In addition, pH was adjusted to 7.01 (InPro3250, Mettler-Toledo GmbH, Gießen, Germany) during the whole cultivation using 2 M NaOH. To avoid foam formation, sterile-filtered Ucolub (FRAGOL, Mülheim, Germany) was occasionally added to the growth medium.

**Correlation between optical density (OD_600nm_) and cell dry weight (CDW)**

Cell concentration was determined as optical density at a wavelength of 600 nm (Libra S11, Biochrome, Cambridge, UK). To establish correlations between optical density (OD_600nm_) and cell dry weight (CDW), known volumes of cell suspension were filtered with nylon filter (0.45 µm, Whatman, Dassel, Germany). To remove medium, filters were successively washed with NaCl solutions matching the ionic strength of the medium and deionized water. Finally, filters were dried at 105 °C until they reached a constant weight. For cells growing with 500 mg L^-1^ of NaCl, a correlation factor of 0.2173 g_CDW_·L^-1^ = 1 OD_600nm_ was obtained, whereas cultivations with 35.57 and 70.63 g L^-1^ NaCl concentration resulted in an identical correlation factor of 0.1917 g_CDW_·L^-1^ = 1 OD_600nm_.

**Biomass composition**

***Proteins*** were extracted from cells as described for proteome analysis and ***protein content*** was determined combining both the Waddel’s method (Wolf, 1983) and measurements at 205 nm (Goldfarb et al., 1951; Tombs et al., 1959). For measurement at 205 nm, calibration was carried out with bovine serum albumin, lysozyme, catalase and pepsin. ***Amino acid composition of cell proteins*** was determined by hydrolysis (105 °C, 22 h) of cells with 150 µL mg_CDW_^-1^ of 6 M HCl / 0.1 % phenol (Fountoulakis and Lahm, 1998). Hydrolysates were evaporated under a nitrogen stream, resuspended in 200 µM α-aminobutyrate (internal standard) and concentration of free amino acids was determined as described for metabolome analysis. Finally, measured concentrations were corrected for loss during hydrolysis and protein composition was adjusted to consider the impact of intracellular amino acids.

***RNA content*** was evaluated by spectrometric measurement at 260 nm (Nanodrop 1000, Thermo Fisher Scientific, Waltham, MA, USA) as previously described (Benthin et al., 1991) except that washing was only performed once with 700 mM of HClO_4_.

***DNA content*** was also determined by spectrometric measurement at 260 nm with a NanoDrop 1000™ (Thermo Fisher Scientific, Waltham, MA, USA). First, cells were harvested and enzymatically digested with DNA lysis buffer (25 mM of Tris, 25 mM of EDTA, 30 mM of Saccharose, pH 8) for 30 min at 30 °C and 350 min^-1^ (Thermomixer comfort, Eppendorf AG, Hamburg, Germany). To complete extraction, cells were subsequently mechanically disrupted with soda-lime glass beads (20 % v/v, 0.038-0.045 mm, Worf Glaskugeln GmbH, Mainz, Germany) in a FastPrep®-24 (3 x 1 min, 6.5 m s^-1^, 4 °C, MP Biomedical, Santa Ana, CA, USA). Cell debris were discarded (13,000 min^-1^, 5 min, 4 °C, Microcentrifuge 5415R, Eppendorf AG, Hamburg, Germany) and supernatant treated for RNA digestion with 140 µL RES solution containing 60 µg L^-1^ of RNase ("Plasmid DNA purification", Macherey-Nagel, Düren, Germany). Subsequently, DNA underwent a two-step purification comprising a first separation with 700 µL Roti-phenol-chloroform-isoamylalcohol and a second with 700 µL of chloroform. Both separations were supported by a 10 min centrifugation at 13200 min^-1^ and 4 °C (Microcentrifuge 5415R, Eppendorf AG, Hamburg, Germany). The next step involved DNA precipitation with 65 µL of 3 M sodium acetate (pH 5.5) and 1.3 mL of ice-cold pure ethanol. After centrifugation, the supernatants were carefully discarded and precipitated DNA could finally be washed with 70 % ethanol, dried in a vacuum concentrator 5301 (Eppendorf AG, Hamburg, Germany) and solved in 100 µL of ultrapure water.

***Glycogen*** was enzymatically isolated from the cytosol with lysozyme (40,000 U mg^-1^, Sigma-Aldrich (Fluka), Steinheim, Germany) and converted to glucose with amyloglycosidase (59.9 U mg^-1^, Sigma-Aldrich (Fluka), Steinheim, Germany). To this end, sedimented cells were resuspended in 500 μL lysis buffer (3.15 g L^-1^ TRIS, 3·10^-2^ g L^-1^ lysozyme, 1 g L^-1^ amyloglycosidase, pH 7) and incubated at 37 °C and 400 min^-1^ for 3 h (Thermomixer comfort, Eppendorf AG, Hamburg, Germany). Finally, cell extracts were centrifuged (13200 min^-1^, 5 min, 4 °C, Microcentrifuge 5415R, Eppendorf AG, Hamburg, Germany) and glucose concentration in supernatants was determined enzymatically using a YSI 2700 SELECT™ Biochemistry Analyzer (YSI incorporated, Yellow Springs, Ohio, USA).

To estimate the ***lipid fraction and its composition***, 400 mg of biomass obtained from eight biological replicates were freeze-dried (Alpha 1-4 LD, Martin Christ Gefriertrocknungsanlagen GmbH, Osterode, Germany) und underwent a lipid extraction according to a modified Folch method, involving a chloroform/methanol/acidified salt solution (2:1:0.8) (Folch et al., 1957). All collected fractions were then converted to fatty acid methyl esters (FAME) and subsequently quantified with GLC according to the AOCS official method Ce 1b-89 (AOCS, 1999). To do so, the fractions were first transferred into a screw-cap tube containing 0.5 mg of C23:0 methyl ester internal standard and saponification was performed with 1.5 mL of 500 mN alcoholic sodium hydroxide (100 °C, 5 min). The solutions were then cooled, completed with 2 mL of 12 % (w/w) boron trifluoride (BF3) in methanol and incubated anew for 30 min at 100 °C. After incubation, the samples were cooled again and the methyl ester extraction was achieved by addition of 1 mL of isooctane and 5 mL of saturated NaCl (360 g mL^-1^). The tubes were gently vortexed and rested until phase separation. The upper isooctane layer was then carefully collected and the bottom phase extracted once again. Finally, corresponding isooctane layers were brought together and concentrated to a final volume of 1 mL under a nitrogen stream. The concentrates were finally subjected to GLC-analysis (**Table**).

**Table:** GC parameters used for the analysis of fatty acid methyl esters

**Parameter Setting**

**Detector temperature** 300 °C

**Detector mode** Constant make up flow

**Hydrogen flow** 40 mL∙min^-1^

**Air flow** 450 mL∙min^-1^

**Make up flow** 45 mL∙min^-1^

**Make up gas** Helium (He)

**Injector temperature** 230 °C

**Injector mode** Split

**Split ratio** 50:1

**Injection volume** 1 mL

**Temperature programme** 170 °C for 3 min

170-220 at 4 °C∙min^-1^

220 °C for 10 min

Content of the ***peptidoglycan*** polymer was derived from the sole concentrations of meso-diaminopimelate (m-DAP) in biomass hydrolysates. In fact, in *B. megaterium*, m-DAP can only be integrated in peptidoglycan or converted to lysine and its intracellular level is therefore negligible. Hence, knowledge of the m-DAP content in biomass hydrolysates and of the average molar composition of peptidoglycan monomer units is sufficient to deduce the global peptidoglycan content. In *B. megaterium*, this molar composition consist of 1.8 alanine, 1 glutamate, 1 m-DAP, 1 N-acetylglucosamine and 1 N-acetylmuramic acid (Van Heijenoort et al., 1969; Vollmer et al., 2008). In this study, concentration of m-DAP was determined by HPLC as described for intracellular amino acids using the same biomass hydrolysates as for the determination of amino acid composition of proteins and applying the same correction factor. ***Teichoic acid and lipoteichoic acid contents*** were estimated supposing that ratio with peptidoglycan subunit is similar to that observed by Kohlstedt et al. in *B. subtilis* (Kohlstedt et al., 2014).

**Supplementary Figures and Tables**

- 1. **Supplementary Figures**


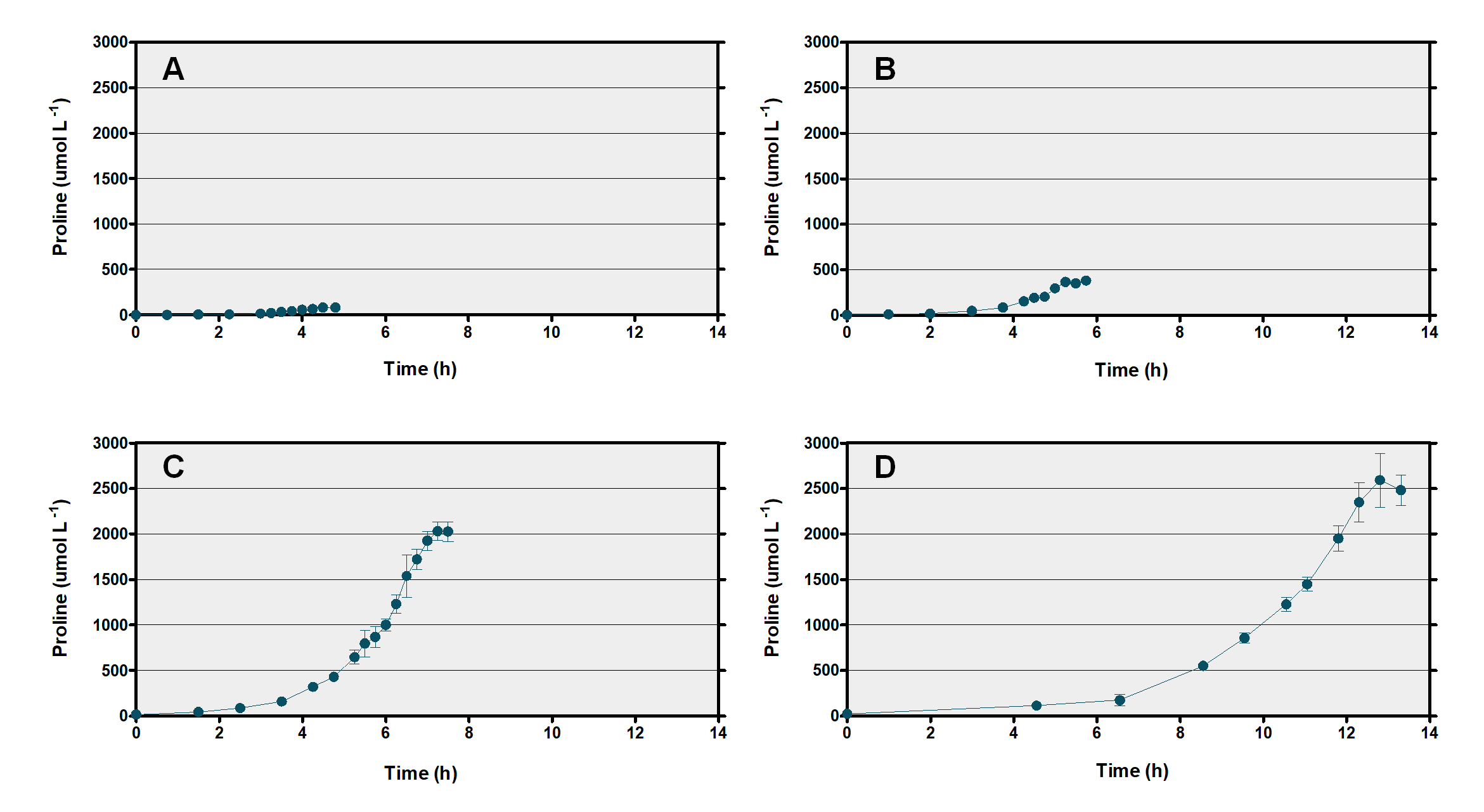
**Supplementary Figure 1: Intracellular proline accumulation at 0 M (A), 0.3 M (B), 0.6 M (C) and 1.2 M (D) over time.**


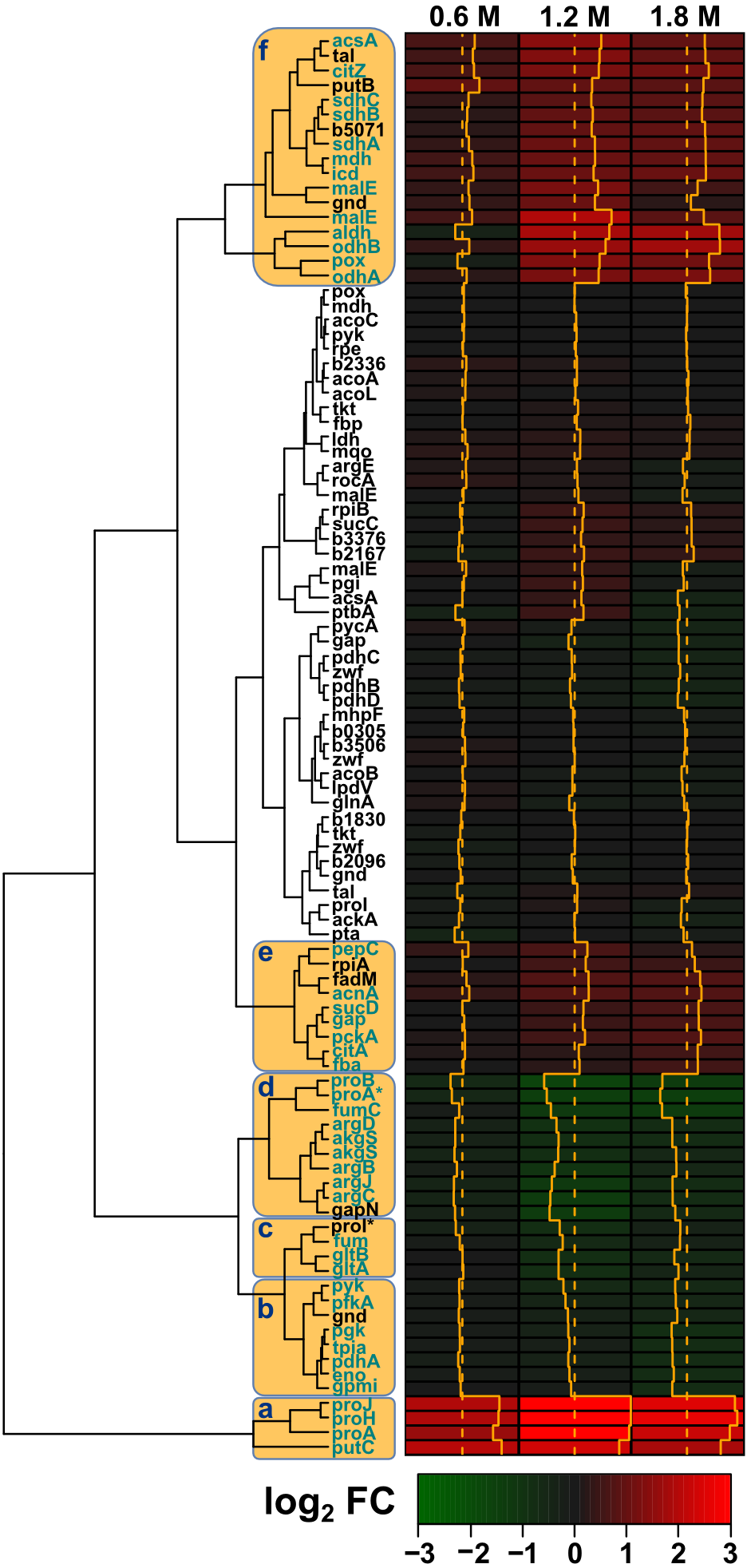


*****

**Supplementary Figure 2: Hierarchical clustering of gene expression of 97 selected genes of the central carbon, proline and arginine metabolism of *B. megaterium* DSM319.** Expression is indicated as log_2_ fold change (log_2_ FC) compared to expression at 37 °C. Six main regulation clusters can be identified: **(a)** Genes coding for enzymes involved in proline biosynthesis under osmotic stress, **(b)** Genes coding for glycolytic enzymes, **(c)** Genes with unexpectedly low transcription level under salt stress, **(d)** Genes encoding key enzymes of the arginine and anabolic proline metabolism, **(e)** Genes coding for enzymes of the glycolysis, TCA cycle and junction of these two pathways, **(f)** Genes encoding enzymes from the TCA cycle or involved in overflow metabolism.


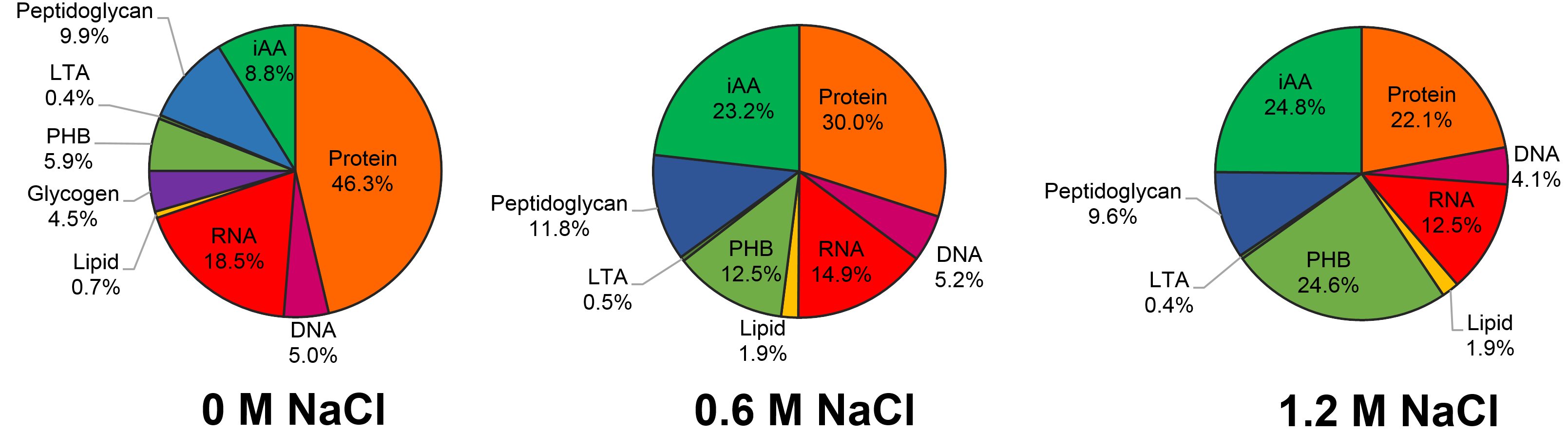


**Supplementary Figure 3: Macromolecular composition of *B. megaterium* DSM319 growing in M9 minimal medium supplemented with 0, 0.6 and 1.2 NaCl, respectively.** Protocols used for the determination of each cellular component are described above. **DNA:** deoxyribonucleic acid, **iAA:** intracellular amino acids, **LTA:** lipoteichoic acids, **PHB:** polyhydroxybutyrate, **RNA:** ribonucleic acid.

**Supplementary Figure 4: Principal component analysis (PCA) followed by hierarchical clustering (HCPC) on fold change between protein concentrations at 0.6, 1.2 and 1.8 M NaCl and their concentration in cells growing without additional NaCl supplementation.** For more clarity, BMD was replaced by B in protein names and only the 125 proteins most relevant for the PCA construction are presented. Eight key clusters can be detected. Given the important weight of the first principal component (84,93 %) and the strong dependence of the three conditions to this dimension (> 0.8), clusters described from left to right the transition from proteins whose concentration is strongly reduced (**Cluster 1**) to those whose concentration is strongly increased under salt stress (**Cluster 8**).

**2.1 Supplementary Tables**

**Supplementary Table 1: Gene expression levels in *B. megaterium* DSM319 grown with 0.6, 1.2 and 1.8 M NaCl, respectively.** Data are given as fold change (FC) of transcript concentrations compared to their values in cells grown without additional NaCl supplementation. They were obtained from microarray experiments carried out using four biological replicates for each cultivation condition. Only genes whose expression was at least 1.75-fold up- (**red**) or down-regulated (**blue**) with a p-value < 0.05 at 0.6, 1.2 and/or 1.8 M NaCl were considered as significantly regulated and listed.

| **Gene product** | **Gene_id** | **Gene symbol** | **0.6 M** | **1.2 M** | **1.8 M** |
| --- | --- | --- | --- | --- | --- |
| Sigma-F transcribed protein CsfB | *bmd_0044* | *csfB* | -1.43 | **-2.45** | **-1.87** |
| Transition state regulatory protein AbrB | *bmd_0054* | *abrB* | -1.39 | **-2.07** | -1.45 |
| Control of biofilm formation | *bmd_0061* | *veg* | 1.09 | 1.51 | **2.26** |
| 50S ribosomal protein L25/general stress protein Ctc | *bmd_0069* | *ctc* | -1.37 | **3.51** | **3.26** |
| Conserved hypothetical protein | *bmd_0108* |  | 1.01 | **1.84** | -1.02 |
| KinB sporulation signaling pathway activation protein | *bmd_0173* | *kbaA* | 1.28 | 1.68 | **1.85** |
| Hypothetical protein | *bmd_0186* |  | -1.32 | **1.88** | **2.45** |
| RNA polymerase sigma-W factor | *bmd_0187* | *sigW* | -1.07 | **1.93** | 1.05 |
| Anti-sigma-W factor | *bmd_0188* | *rsiW* | -1.01 | **1.83** | -1.04 |
| Putative membrane protein | *bmd_0208* |  | 1.37 | **1.80** | 1.51 |
| Anti-sigma B factor antagonist | *bmd_0227* | *rsbV* | -1.22 | **1.75** | **1.91** |
| Serine-protein kinase RsbW (anti-sigma B factor) | *bmd_0228* | *rsbW* | -1.19 | **1.84** | 1.66 |
| RNA polymerase sigma-B factor | *bmd_0229* | *sigB* | -1.17 | **1.88** | 1.71 |
| Phosphoserine phosphatase RsbX | *bmd_0230* | *rsbX* | -1.13 | **1.75** | 1.68 |
| Putative Redox-sensing transcriptional repressor rex | *bmd_0255* |  | 1.35 | **1.87** | 1.27 |
| Twin arginine-targeting protein translocase, TatA/E family protein | *bmd_0256* | *tatA* | 1.27 | **1.88** | 1.33 |
| Hypoxanthine/guanine permease | *bmd_0266* | *pbuG* | -1.31 | **-5.31** | **-3.53** |
| Phosphoribosylaminoimidazole carboxylase, catalytic subunit | *bmd_0271* | *purE* | 1.24 | **-3.25** | **-2.72** |
| Phosphoribosylaminoimidazole carboxylase, ATPase subunit | *bmd_0272* | *purK* | 1.39 | **-2.79** | **-2.54** |
| Adenylosuccinate lyase | *bmd_0273* | *purB* | 1.48 | **-2.30** | **-2.35** |
| Phosphoribosylaminoimidazole-succinocarboxamide synthase | *bmd_0274* | *purC* | 1.33 | **-2.46** | **-2.32** |
| Phosphoribosylformylglycinamidine synthase, purS protein | *bmd_0275* | *purS* | 1.44 | **-2.48** | **-2.44** |
| Phosphoribosylformylglycinamidine synthase I | *bmd_0276* | *purQ* | 1.45 | **-2.22** | **-1.97** |
| Phosphoribosylformylglycinamidine synthase II | *bmd_0277* | *purL* | 1.53 | **-2.16** | **-2.57** |
| Amidophosphoribosyltransferase | *bmd_0278* | *purF* | 1.49 | **-2.14** | **-2.23** |
| Phosphoribosylformylglycinamidine cyclo-ligase | *bmd_0279* | *purM* | 1.54 | **-2.11** | **-2.11** |
| Phosphoribosylglycinamide formyltransferase | *bmd_0280* | *purN* | 1.39 | **-2.23** | **-2.25** |
| Bifunctional purine biosynthesis protein PurH | *bmd_0281* | *purH* | 1.39 | **-2.16** | **-2.10** |
| Phosphoribosylamine--glycine ligase | *bmd_0282* | *purD* | 1.41 | **-2.13** | **-2.09** |
| TrpR like protein, YerC/YecD | *bmd_0285* |  | -1.28 | **-1.99** | -1.71 |
| Conserved hypothetical protein | *bmd_0309* |  | -1.01 | **1.83** | **-1.96** |
| Amino acid permease | *bmd_0315* |  | -1.37 | **-2.04** | **-1.97** |
| Conserved hypothetical protein | *bmd_0317* |  | 1.17 | **4.41** | **3.96** |
| Conserved hypothetical protein | *bmd_0318* |  | 1.26 | **2.51** | **2.23** |
| Intracellular protease, PfpI family | *bmd_0331* |  | 1.21 | **1.95** | 1.48 |
| Conserved hypothetical protein | *bmd_0333* |  | 1.06 | **2.48** | 1.26 |
| Conserved hypothetical protein | *bmd_0364* |  | **2.30** | **25.11** | **51.61** |
| Intracellular protease, PfpI family | *bmd_0368* |  | -1.40 | 1.69 | **2.35** |
| Conserved hypothetical protein | *bmd_0376* |  | -1.48 | **-1.99** | -1.66 |
| Conserved hypothetical protein | *bmd_0394* |  | **-1.96** | **-2.55** | -1.64 |
| Membrane-bound metal-dependent hydrolase (DUF457) | *bmd_0402* |  | 1.13 | **2.23** | 1.69 |
| Undecaprenol kinase | *bmd_0452* | *uppP* | -1.19 | **-2.01** | -1.74 |
| Proton/sodium-glutamate symport protein | *bmd_0453* |  | **2.04** | **2.48** | **1.88** |
| Biotin synthase | *bmd_0460* | *bioB* | 1.33 | **1.87** | **2.25** |
| Putative exported cell wall-binding protein | *bmd_0478* | *yocH* | 1.01 | **-3.34** | -1.75 |
| Hypothetical protein | *bmd_0485* |  | -1.13 | **1.92** | **1.80** |
| Conserved hypothetical protein | *bmd_0515* |  | 1.00 | 1.13 | **-1.79** |
| Conserved hypothetical protein | *bmd_0521* |  | -1.12 | **3.66** | **2.52** |
| Glycerol uptake facilitator protein | *bmd_0533* | *glpF* | 1.06 | **2.04** | 1.31 |
| Glycerol kinase | *bmd_0534* | *glpK* | 1.09 | **2.17** | 1.23 |
| L-cystine import ABC transporter, ATP-binding protein TcyC | *bmd_0545* | *tcyC* | -1.29 | **-1.85** | **-2.02** |
| L-cystine import ABC transporter, permease protein TcyB | *bmd_0546* | *tcyB* | -1.27 | **-2.04** | **-2.03** |
| L-cystine import ABC transporter, L-cystine-binding protein TcyA | *bmd_0547* | *tcyA* | -1.38 | **-2.30** | **-2.62** |
| Conserved hypothetical protein | *bmd_0577* |  | -1.29 | **-1.88** | -1.06 |
| O-acetyltransferase | *bmd_0591* |  | 1.02 | **2.46** | 1.67 |
| PAP2 family protein | *bmd_0597* |  | -1.11 | **-1.82** | -1.66 |
| Monooxygenase | *bmd_0599* |  | -1.04 | **1.75** | 1.27 |
| Extracellular solute-binding protein | *bmd_0606* |  | 1.21 | **2.08** | 1.46 |
| Conserved hypothetical protein | *bmd_0638* |  | 1.31 | **2.10** | **1.94** |
| Conserved hypothetical protein | *bmd_0676* |  | 1.15 | **2.39** | **2.04** |
| N-acetyl-gamma-glutamyl-phosphate reductase | *bmd_0678* | *argC* | -1.39 | **-2.50** | -1.70 |
| Arginine biosynthesis bifunctional protein ArgJ | *bmd_0679* | *argJ* | -1.38 | **-2.28** | -1.71 |
| Acetylglutamate kinase | *bmd_0680* | *argB* | -1.21 | **-2.07** | -1.48 |
| Acetylornithine aminotransferase | *bmd_0681* | *argD* | -1.28 | **-1.96** | -1.73 |
| NAD dependent epimerase/dehydratase | *bmd_0685* |  | -1.37 | **2.69** | **2.02** |
| Oligopeptide ABC transporter, ATP-binding protein AppF | *bmd_0700* | *appF* | 1.37 | **2.06** | **1.87** |
| Oligopeptide ABC transporter, oligopeptide-binding protein AppA | *bmd_0701* | *appA* | 1.53 | **3.12** | **2.03** |
|  |  |  |  |  |  |
| **Gene product** | **Gene_id** | **Gene**  **symbol** | **0.6 M** | **1.2 M** | **1.8 M** |
| Oligopeptide ABC transporter, permease protein AppB | *bmd_0702* | *appB* | 1.47 | **2.38** | **2.02** |
| Oligopeptide ABC transporter, permease protein AppC | *bmd_0703* | *appC* | 1.21 | **1.89** | 1.74 |
| Amino acid permease | *bmd_0712* |  | 1.03 | -1.39 | **-2.05** |
| Arginine/ornithine antiporter | *bmd_0713* | *arcD* | -1.15 | **-2.31** | **-1.82** |
| Competence-associated adapter protein | *bmd_0719* | *mecA* | -1.08 | **1.96** | 1.28 |
| 6-phosphogluconate dehydrogenase (decarboxylating) | *bmd_0753* | *gnd* | 1.29 | **2.14** | 1.16 |
| Gluconate kinase | *bmd_0754* | *gntK* | 1.37 | **2.28** | 1.51 |
| Transporter, gluconate:H+ symporter (GntP) family | *bmd_0756* |  | **2.00** | **3.86** | **1.86** |
| Conserved hypothetical protein | *bmd_0757* |  | 1.43 | **1.97** | 1.49 |
| Amino acid permease | *bmd_0809* |  | -1.54 | **-3.03** | -1.46 |
| Nucleoside transporter, NupC family | *bmd_0826* |  | -1.24 | **-2.43** | -1.73 |
| Integral membrane protein | *bmd_0832* |  | -1.13 | **-1.89** | -1.36 |
| Homocysteine S-methyltransferase | *bmd_0849* | *ybgG* | 1.22 | **2.03** | 1.24 |
| Hypothetical protein | *bmd_0893* |  | -1.38 | **3.23** | **2.49** |
| Conserved hypothetical protein | *bmd_0894* |  | -1.45 | **3.14** | **2.54** |
| Conserved hypothetical protein | *bmd_0895* |  | -1.09 | **2.16** | **1.93** |
| Ferrous iron transport protein B | *bmd_0896* | *feoB* | -1.14 | **1.84** | **1.84** |
| Transporter, solute:sodium symporter (SSS) family | *bmd_0908* |  | 1.15 | **1.79** | 1.56 |
| Transcriptional regulator, IclR family | *bmd_0911* |  | -1.19 | **2.30** | 1.39 |
| Oxidoreductase, aldo/keto reductase family | *bmd_0912* |  | -1.21 | **2.58** | 1.73 |
| 4-aminobutyrate aminotransferase | *bmd_0945* |  | -1.62 | **-3.43** | **-2.85** |
| Allophanate hydrolase subunit 1 | *bmd_0960* |  | 1.52 | **2.53** | 1.51 |
| Allophanate hydrolase subunit 2 | *bmd_0961* |  | **1.95** | **3.05** | 1.51 |
| LamB/YcsF family protein | *bmd_0962* |  | **1.93** | **3.03** | 1.71 |
| Uncharacterized membrane protein ycsG | *bmd_0963* |  | **1.87** | **3.10** | 1.60 |
| Amidohydrolase | *bmd_0986* |  | -1.19 | **-2.48** | **-2.03** |
| Cold shock protein | *bmd_0987* | *cspB* | -1.38 | **-2.83** | **-1.80** |
| Antioxidant, AhpC/TSA family | *bmd_0991* |  | 1.21 | 1.34 | **1.84** |
| Capsule biosynthesis protein CapB | *bmd_1003* | *capB* | **2.48** | **4.53** | **3.16** |
| Capsule biosynthesis protein CapC | *bmd_1004* | *capC* | **2.17** | **3.81** | **2.90** |
| Capsule biosynthesis protein CapA | *bmd_1005* | *capA* | **2.43** | **4.79** | **2.84** |
| Putative gamma glutamyl transferase | *bmd_1006* |  | **2.36** | **4.23** | **3.08** |
| Hypothetical protein | *bmd_1007* |  | **2.19** | **3.78** | **2.78** |
| CsbD-like protein | *bmd_1013* |  | -1.55 | **2.14** | **3.34** |
| Phosphoenolpyruvate-dependent sugar phosphotransferase system, EIIA 2 domain protein | *bmd_1027* |  | 1.60 | **1.75** | **-2.04** |
| PTS system, lactose/cellobiose specific IIB subunit family protein | *bmd_1028* |  | 1.66 | 1.68 | **-2.17** |
| Oxidoreductase, aldo/keto reductase family | *bmd_1041* |  | 1.31 | **2.99** | 1.73 |
| Hypothetical protein | *bmd_1059* |  | 1.39 | **2.17** | **1.76** |
| Proton/sodium-glutamate symport protein | *bmd_1062* |  | -1.57 | **-2.27** | **-3.69** |
| Endopeptidase LytE | *bmd_1065* | *lytE* | -1.16 | **-2.04** | **-1.78** |
| Hypothetical protein | *bmd_1070* |  | 1.10 | **2.41** | **2.39** |
| Capsule biosynthesis protein CapB | *bmd_1092* | *capB* | -1.74 | **-3.56** | **-2.45** |
| Capsule biosynthesis protein CapC | *bmd_1093* | *capC* | -1.66 | **-3.51** | **-2.81** |
| Capsule biosynthesis protein CapA | *bmd_1094* | *capA* | -1.74 | **-2.87** | **-2.00** |
| Gamma-glutamyltransferase | *bmd_1095* | *ggt* | **-1.78** | **-3.07** | **-2.50** |
| Putative peptidoglycan binding domain protein | *bmd_1096* |  | **-2.03** | **-3.76** | **-2.49** |
| Conserved hypothetical protein | *bmd_1102* |  | -1.21 | -1.58 | -1.48 |
| Conserved hypothetical protein | *bmd_1104* |  | -1.16 | **-1.87** | -1.33 |
| UTP-glucose-1-phosphate uridylyltransferase | *bmd_1114* | *galU* | -1.44 | **-2.85** | **-1.91** |
| Galactosyl transferase cpsE | *bmd_1115* |  | -1.39 | **-2.17** | -1.58 |
| Putative membrane protein | *bmd_1116* |  | -1.34 | **-2.01** | -1.52 |
| Glycosyl transferase, family 2 | *bmd_1117* |  | -1.34 | **-2.28** | -1.64 |
| Glycosyl transferase, family 2 | *bmd_1118* |  | -1.38 | **-2.27** | -1.67 |
| Glycosyl transferase, group 1 | *bmd_1119* |  | -1.43 | **-2.07** | -1.73 |
| Glycosyl transferase, group 1 | *bmd_1120* |  | -1.36 | **-2.43** | **-1.75** |
| Polysaccharide biosynthesis protein | *bmd_1121* |  | -1.41 | **-2.57** | **-2.01** |
| UDPglucose 6-dehydrogenase | *bmd_1122* |  | -1.39 | **-2.16** | **-2.18** |
| Chain length determinant family protein | *bmd_1123* |  | -1.33 | **-1.77** | -1.49 |
| Tyrosine-protein phosphatase capC | *bmd_1125* |  | 1.09 | **1.97** | 1.72 |
| UTP-glucose-1-phosphate uridylyltransferase | *bmd_1126* | *galU* | 1.09 | 1.74 | 1.47 |
| Membrane-bound protein lytR | *bmd_1127* |  | -1.21 | **-1.85** | -1.64 |
| Tyrosine-protein phosphatase | *bmd_1129* | *ywqE* | -1.06 | -1.73 | -1.50 |
| Pyruvate oxidase | *bmd_1131* |  | -1.20 | **2.41** | **2.26** |
| Flavoenzyme | *bmd_1132* | *yerD* | -1.06 | **1.77** | 1.56 |
| Hypothetical protein | *bmd_1154* |  | 1.30 | **2.16** | **1.81** |
| Lactoylglutathione lyase | *bmd_1173* |  | 1.20 | **3.05** | **2.02** |
| Conserved hypothetical protein | *bmd_1174* |  | 1.27 | **3.25** | **2.13** |
| Conserved hypothetical protein | *bmd_1178* |  | -1.54 | **2.45** | **1.94** |
| Hypothetical protein | *bmd_1185* |  | 1.02 | **-2.55** | -1.65 |
| Hypothetical protein | *bmd_1186* |  | -1.16 | **-2.06** | -1.45 |
| Hypoxanthine/guanine permease | *bmd_1193* | *pbuO* | -1.39 | **-3.12** | **-1.91** |
| Transcriptional regulator, MarR family | *bmd_1200* |  | -1.23 | **-1.84** | -1.42 |
| Polyhydroxyalkanoic acid inclusion protein PhaP | *bmd_1211* | *phaP* | 1.29 | **2.17** | **2.16** |
| Poly-beta-hydroxybutyrate-responsive repressor | *bmd_1212* | *phaQ* | 1.12 | **1.79** | 1.42 |
| NAD+ synthetase | *bmd_1223* | *nadE* | -1.18 | -1.48 | **-1.77** |
| 5-methylthioribose kinase | *bmd_1231* | *mtnK* | 1.07 | **-1.83** | -1.66 |
|  |  |  |  |  |  |
|  |  |  |  |  |  |
|  |  |  |  |  |  |
|  |  |  |  |  |  |
| **Gene product** | **Gene ID** | **Gene symbol** | **0.6 M** | **1.2 M** | **1.8 M** |
| 2,3-diketo-5-methylthiopentyl-1-phosphate enolase | *bmd_1234* | *mtnW* | 1.03 | **-1.99** | **-1.75** |
| 2-hydroxy-3-keto-5-methylthiopentenyl-1- phosphate phosphatase | *bmd_1235* | *mtnX* | -1.03 | **-2.00** | **-1.76** |
| Methylthioribulose-1-phosphate dehydratase | *bmd_1236* | *mtnB* | -1.04 | **-1.99** | **-1.79** |
| 1,2-dihydroxy-3-keto-5-methylthiopentene dioxygenase | *bmd_1237* | *mtnD* | -1.05 | **-2.17** | **-1.79** |
| Ribose ABC transporter, ribose-binding protein RbsB | *bmd_1238* | *rbsB* | -1.14 | **-2.73** | **-2.38** |
| Ribose ABC transporter, ATP-binding protein RbsA | *bmd_1239* | *rbsA* | -1.27 | **-3.01** | **-1.93** |
| Ribose ABC transporter, permease protein RbsC | *bmd_1240* | *rbsC* | -1.27 | **-2.33** | -1.56 |
| ATP-dependent Clp protease, ATP-binding subunit ClpE | *bmd_1249* | *clpE* | -1.27 | **1.97** | 1.35 |
| Putative Na+/H+ antiporter NhaC | *bmd_1250* | *nhaC* | -1.35 | **-2.62** | **-2.71** |
| Conserved hypothetical protein | *bmd_1259* |  | -1.52 | **-2.19** | -1.24 |
| Conserved hypothetical protein | *bmd_1261* |  | 1.73 | **3.53** | **2.17** |
| Phosphotransferase system (PTS) enzyme I | *bmd_1284* | *ptsI* | 1.15 | **1.92** | 1.28 |
| Conserved hypothetical protein | *bmd_1285* |  | -1.42 | **-2.75** | -1.46 |
| LrgA family protein | *bmd_1286* |  | -1.41 | **-2.46** | -1.55 |
| Conserved hypothetical protein | *bmd_1288* |  | -1.43 | **-2.62** | -1.59 |
| Mechanosensitive ion channel | *bmd_1313* |  | -1.03 | **1.84** | 1.37 |
| 6-phosphogluconate dehydrogenase, decarboxylating | *bmd_1316* | *gnd* | -1.25 | **-2.39** | **-2.14** |
| Arginine decarboxylase | *bmd_1333* | *speA* | -1.55 | **-3.18** | **-2.17** |
| Conserved hypothetical protein | *bmd_1358* |  | 1.21 | **1.91** | **1.87** |
| Penicillin-binding protein 1A/1B | *bmd_1383* | *ponA* | -1.36 | -1.61 | **-1.77** |
| Conserved hypothetical protein | *bmd_1389* |  | -1.35 | **-1.92** | -1.29 |
| Conserved hypothetical protein | *bmd_1399* |  | 1.21 | 1.73 | 1.58 |
| Sodium/proline symporter, frameshift | *bmd_1401* | *opuE* | **1.84** | **3.20** | **2.39** |
| Conserved hypothetical protein | *bmd_1412* |  | -1.35 | **-2.16** | **-1.79** |
| Modulator of lia operon expression | *bmd_1416* |  | 1.06 | **1.87** | 1.04 |
| Putative RNA methylase protein family (UPF0020) | *bmd_1421* |  | 1.04 | **1.77** | 1.55 |
| Carboxypeptidase Taq (M32) metallopeptidase | *bmd_1431* |  | 1.38 | **2.01** | **2.14** |
| Xanthine phosphoribosyltransferase | *bmd_1432* | *xpt* | 1.08 | **-1.91** | -1.55 |
| Xanthine permease | *bmd_1433* |  | 1.09 | **-1.97** | -1.44 |
| Succinate-semialdehyde dehydrogenase (NADP+) - general stress protein | *bmd_1435* |  | 1.35 | **2.79** | **2.28** |
| Succinate-semialdehyde dehydrogenase (NADP+) - GABA utilization | *bmd_1435* |  | 1.35 | **2.79** | **2.28** |
| Conserved hypothetical protein | *bmd_1442* |  | -1.17 | **-1.80** | -1.65 |
| Hypothetical protein | *bmd_1443* |  | -1.10 | **-1.80** | -1.60 |
| Cold shock protein | *bmd_1450* | *cspD* | 1.32 | 1.65 | **2.01** |
| Acetyltransferase, GNAT family | *bmd_1466* |  | -1.26 | **-1.77** | -1.57 |
| Major facilitator transporter family protein (putative permease) | *bmd_1484* |  | -1.27 | **-2.13** | -1.46 |
| Amidohydrolase | *bmd_1487* |  | 1.31 | **1.99** | 1.40 |
| L-seryl-tRNA(Sec) selenium transferase | *bmd_1488* | *selA* | 1.48 | **1.97** | 1.33 |
| Conserved hypothetical protein | *bmd_1489* |  | 1.40 | **2.23** | 1.55 |
| 2-dehydro-3-deoxygluconokinase | *bmd_1490* |  | 1.48 | **1.99** | 1.28 |
| Transcriptional regulator, GntR family | *bmd_1491* |  | 1.21 | **1.88** | 1.38 |
| Transporter, gluconate:H+ symporter (GntP) family | *bmd_1492* |  | 1.14 | **2.22** | 1.65 |
| Conserved hypothetical protein | *bmd_1493* |  | 1.23 | **2.25** | 1.65 |
| Conserved hypothetical protein | *bmd_1494* |  | 1.31 | **2.46** | **1.77** |
| Ferrichrome import ABC transporter, ferrichrome-binding protein FhuD | *bmd_1509* | *fhuD* | -1.33 | **1.97** | 1.17 |
| Ferrichrome import ABC transporter, ATP-binding protein FhuC | *bmd_1510* | *fhuC* | -1.31 | **2.03** | 1.18 |
| Putative Drug Resistance Transporter Family | *bmd_1513* |  | -1.51 | -1.23 | **2.27** |
| 2,5-diketo-D-gluconic acid reductase B | *bmd_1514* |  | 1.00 | **2.06** | **2.34** |
| HTH-type transcriptional activator hxlR | *bmd_1518* |  | 1.26 | **2.03** | 1.41 |
| 3-hexulose-6-phosphate synthase | *bmd_1519* | *hxlA* | 1.17 | **1.78** | 1.27 |
| Two-component sensor histidine kinase/response regulator | *bmd_1527* |  | -1.20 | **-1.95** | **-1.85** |
| Methyltransferase, CheR family | *bmd_1528* |  | -1.25 | **-1.80** | -1.53 |
| Ferritin-like domain protein | *bmd_1538* |  | -1.30 | **2.81** | **3.37** |
| Aldehyde dehydrogenase (NAD) Family Protein | *bmd_1546* |  | -1.31 | **3.76** | **3.32** |
| Glucose starvation-inducible protein B | *bmd_1557* |  | 1.37 | **5.46** | **12.05** |
| Putative methionine import ABC transporter, methionine-binding protein Met | *bmd_1578* | *met* | -1.20 | **-2.03** | **-1.86** |
| Methionine import ABC transporter, ATP-binding protein MetN | *bmd_1579* | *metN* | -1.25 | **-2.16** | -1.67 |
| Putative methionine import ABC transporter, permease protein Met | *bmd_1580* | *met* | -1.27 | **-2.16** | **-1.86** |
| 2,5-diketo-D-gluconic acid reductase A | *bmd_1595* |  | -1.23 | **-1.78** | -1.60 |
| Endopeptidase LytF (cell wall hydrolase) | *bmd_1625* | *lytF* | **-1.97** | **-4.29** | **-3.02** |
| Conserved hypothetical protein | *bmd_1626* |  | -1.72 | **-1.88** | -1.37 |
| Methylmalonate-semialdehyde dehydrogenase (acylating) | *bmd_1655* | *mmsA* | 1.19 | **1.78** | 1.33 |
| Conserved hypothetical protein | *bmd_1667* |  | -1.01 | **3.07** | **2.81** |
| Spore coat protein F | *bmd_1668* | *cotF* | 1.01 | **3.03** | **2.82** |
| Hypothetical protein | *bmd_1681* |  | 1.34 | **2.75** | **2.01** |
| Cold shock protein | *bmd_1682* | *cspA* | -1.30 | 1.62 | **2.50** |
| CBS domain pair family protein | *bmd_1697* |  | -1.02 | **2.25** | **2.42** |
| Glutamate dehydrogenase | *bmd_1700* |  | **-2.35** | **-3.76** | -1.37 |
| 4-hydroxy 2-oxovalerate aldolase | *bmd_1715* |  | -1.05 | **-2.22** | **-2.19** |
| Conserved hypothetical protein | *bmd_1761* |  | -1.34 | **2.81** | **2.10** |
| hut operon positive regulatory protein | *bmd_1769* |  | 1.23 | **2.20** | 1.47 |
| Spore coat protein F | *bmd_1779* | *cotF* | -1.03 | **3.89** | **3.39** |
| Hypothetical protein | *bmd_1780* |  | -1.04 | **3.94** | **3.02** |
| Glucose starvation-inducible protein B (General stress protein B) | *bmd_1781* |  | -1.05 | **4.11** | **3.97** |
| Stress response protein YsnF | *bmd_1782* | *ysnF* | -1.23 | **2.75** | 1.71 |
| CBS domain pair family protein | *bmd_1800* |  | -1.08 | **2.46** | **2.51** |
|  |  |  |  |  |  |
|  |  |  |  |  |  |
|  |  |  |  |  |  |
|  |  |  |  |  |  |
| **Gene product** | **Gene ID** | **Gene symbol** | **0.6 M** | **1.2 M** | **1.8 M** |
| Protein of unknown funtion (DUF77) | *bmd_1801* |  | -1.22 | **2.43** | **2.22** |
| Hypothetical protein | *bmd_1802* |  | -1.11 | **3.25** | **3.01** |
| Oligopeptide ABC transporter, oligopeptide-binding protein | *bmd_1832* |  | 1.38 | -1.66 | **-2.17** |
| Glyceraldehyde-3-phosphate dehydrogenase (NADP) | *bmd_1844* | *gapN* | -1.31 | **-2.87** | -1.54 |
| Conserved hypothetical protein | *bmd_1890* |  | -1.02 | **1.82** | **1.97** |
| ***Two-component sensor histidine kinase*** | *bmd_1892* |  | **3.86** | -1.37 | **-1.87** |
| ***Two-component response regulator*** | *bmd_1893* |  | **4.38** | -1.23 | **-1.94** |
| ***Conserved hypothetical protein*** | *bmd_1894* |  | **2.60** | -1.02 | -1.08 |
| ***Copper chaperone CopZ (copper-ion-binding protein)*** | *bmd_1895* | *copZ* | **2.60** | -1.03 | -1.33 |
| Putative lipoprotein | *bmd_1898* |  | **3.05** | **-2.01** | **-3.73** |
| Putative peptidoglycan binding domain protein | *bmd_1902* |  | 1.21 | **2.68** | 1.36 |
| Transcriptional regulator, GntR family | *bmd_1914* |  | -1.41 | **-1.80** | -1.28 |
| Tartrate dehydrogenase/decarboxylase | *bmd_1915* | *ycsA* | **2.06** | **3.94** | 1.73 |
| Hypothetical protein | *bmd_1921* |  | -1.64 | -1.23 | **-2.24** |
| Putative metal ABC transporter, metal-binding protein | *bmd_1949* |  | -1.38 | **-3.23** | **-2.73** |
| Putative phosphatase | *bmd_1982* |  | -1.06 | **2.17** | 1.22 |
| Tellurite resistance protein,putative | *bmd_1983* |  | -1.07 | **2.07** | 1.33 |
| Sodium:dicarboxylate symporter | *bmd_1992* |  | 1.19 | **-1.82** | **-1.93** |
| Phenylacetaldehyde dehydrogenase | *bmd_1994* |  | 1.39 | **4.00** | **2.39** |
| Foldase protein PrsA | *bmd_2008* | *prsA* | 1.26 | **1.79** | -1.67 |
| Amino acid/peptide transporter (Peptide:H+ symporter) | *bmd_2012* | *dtpT* | -1.34 | **-2.04** | **-2.21** |
| Cytochrome P450 | *bmd_2035* |  | 1.12 | **3.10** | **1.97** |
| Malate dehydrogenase | *bmd_2037* |  | 1.44 | **3.78** | **1.86** |
| Glutamate synthase, large subunit | *bmd_2055* | *gltA* | 1.00 | **-1.80** | -1.37 |
| Glutamate synthase, small subunit | *bmd_2056* | *gltB* | 1.01 | **-1.80** | -1.60 |
| Tetrahydrofolate dehydrogenase/cyclohydrolase domain protein | *bmd_2060* |  | 1.49 | **-2.11** | **-1.88** |
| Protein of unknown function (DUF161) | *bmd_2083* |  | -1.39 | **-2.62** | **-1.76** |
| Putative lipoprotein | *bmd_2090* |  | -1.15 | **-1.83** | -1.50 |
| Hypothetical protein | *bmd_2099* |  | -1.48 | **2.20** | **2.41** |
| DNA-3-methyladenine glycosylase family protein | *bmd_2107* |  | 1.11 | **1.77** | 1.41 |
| Conserved hypothetical protein | *bmd_2128* |  | 1.01 | 1.74 | **1.90** |
| Hypothetical protein | *bmd_2153* |  | 1.12 | **2.31** | 1.53 |
| S1 RNA binding domain protein | *bmd_2164* |  | -1.27 | **-2.19** | **-1.82** |
| 3-hydroxybutyrate dehydrogenase | *bmd_2166* |  | 1.11 | **2.77** | **1.76** |
| NADH dehydrogenase | *bmd_2191* |  | **2.71** | **2.23** | **-4.49** |
| General stress protein 17M | *bmd_2208* |  | -1.37 | **3.32** | **3.56** |
| 1,4-dihydroxy-2-naphthoate octaprenyltransferase | *bmd_2219* | *menA* | -1.19 | **-1.79** | -1.38 |
| Protein of unknown function (DUF1696) | *bmd_2222* |  | 1.19 | 1.57 | **2.12** |
| Major Facilitator Superfamily protein | *bmd_2223* |  | -1.04 | 1.23 | **1.83** |
| Conserved hypothetical protein | *bmd_2227* |  | -1.33 | **-2.03** | -1.08 |
| RNA polymerase sigma factor, sigma-70 family | *bmd_2228* |  | -1.38 | **-1.96** | -1.23 |
| Organic hydroperoxide resistance protein | *bmd_2231* | *ohrB* | **-1.91** | 1.05 | 1.37 |
| Peptidoglycan-binding protein | *bmd_2238* |  | 1.21 | **-2.48** | -1.67 |
| Pyrroline-5-carboxylate reductase | *bmd_2243* | *proH* | **3.94** | **10.63** | **6.44** |
| Glutamate 5-kinase | *bmd_2244* | *proJ* | **3.97** | **10.56** | **5.90** |
| Glutamate-5-semialdehyde dehydrogenase | *bmd_2245* | *proA** | **3.18** | **8.75** | **4.88** |
| Conserved hypothetical protein | *bmd_2250* |  | 1.07 | **1.83** | 1.09 |
| Immune inhibitor A metalloprotease | *bmd_2278* | *inhA* | -1.21 | **-2.33** | **-2.83** |
| Fumarate hydratase, class II | *bmd_2279* | *fumC* | -1.14 | **-2.43** | **-2.52** |
| Conserved hypothetical protein | *bmd_2282* |  | -1.60 | 1.32 | **1.89** |
| Bacillolysin precursor (neutral protease) | *bmd_2285* | *nprM* | 1.46 | **6.63** | **3.15** |
| Amino acid permease family protein | *bmd_2362* |  | 1.29 | **2.48** | 1.63 |
| ***Fructoselysine-6-P-deglycase*** | *bmd_2368* | *frlB* | **2.46** | 1.60 | -1.29 |
| Putative cation transporter regulator | *bmd_2410* |  | -1.19 | **1.85** | **1.81** |
| Nitroreductase family protein | *bmd_2414* |  | -1.02 | **1.92** | 1.34 |
| NAD dependent epimerase/dehydratase family | *bmd_2433* |  | 1.23 | **3.10** | **2.18** |
| Conserved hypothetical protein | *bmd_2439* |  | -1.38 | **-1.79** | -1.34 |
| Cell wall-associated protease | *bmd_2442* |  | **-2.01** | **-3.66** | **-2.76** |
| Secreted cell wall DL-endopeptidase | *bmd_2460* | *cwlO* | -1.38 | **-5.46** | **-2.56** |
| Dihydroxy-acid dehydratase | *bmd_2497* | *ilvD* | 1.39 | **2.16** | **2.75** |
| Lysine-specific permease | *bmd_2525* |  | -1.21 | **-2.66** | -1.74 |
| GNAT family acetyltransferase | *bmd_2527* |  | -1.40 | **-2.25** | -1.47 |
| Carbonic anhydrase | *bmd_2585* |  | 1.25 | **5.46** | **2.57** |
| Putative sulfate transport protein (permease activity) | *bmd_2586* |  | 1.15 | **2.93** | **1.98** |
| CbiET protein | *bmd_2601* | *cbiET* | 1.10 | 1.01 | **1.85** |
| Cobalamin biosynthesis protein CbiD | *bmd_2602* | *cbiD* | 1.04 | -1.03 | **2.17** |
| Precorrin-8X methylmutase CbiC | *bmd_2603* | *cbiC* | 1.06 | 1.07 | **2.19** |
| Precorrin-6x reductase | *bmd_2604* | *cbiJ* | 1.06 | 1.01 | **2.13** |
| Sirohydrochlorin cobaltochelatase | *bmd_2605* | *cbiX* | 1.05 | 1.05 | **2.20** |
| Precorrin 3 methylase | *bmd_2606* | *cbiH* | 1.01 | 1.06 | **2.25** |
| Cobalamin biosynthesis protein | *bmd_2607* | *cbiW* | -1.02 | 1.04 | **1.96** |
| IDEAL domain protein | *bmd_2614* |  | 1.33 | **1.92** | **2.05** |
| Ammonium transporter | *bmd_2615* | *amt* | -1.25 | **-2.45** | -1.56 |
| Hypothetical protein | *bmd_2616* |  | -1.23 | **-2.17** | -1.56 |
| Malate permease | *bmd_2629* |  | -1.37 | **-2.31** | -1.45 |
| Conserved hypothetical protein | *bmd_2648* |  | -1.04 | **4.11** | **2.40** |
|  |  |  |  |  |  |
|  |  |  |  |  |  |
|  |  |  |  |  |  |
|  |  |  |  |  |  |
| **Gene product** | **Gene ID** | **Gene symbol** | **0.6 M** | **1.2 M** | **1.8 M** |
| Acetyltransferase, GNAT family | *bmd_2650* |  | -1.45 | **-1.91** | -1.05 |
| Endonuclease V | *bmd_2656* |  | -1.21 | **-1.82** | -1.53 |
| MATE efflux family protein | *bmd_2674* |  | -1.27 | **-2.06** | **-1.76** |
| Tellurium resistance protein, TerD family | *bmd_2685* |  | 1.22 | **2.01** | 1.71 |
| Tellurium resistance protein terD,TerD family | *bmd_2686* |  | 1.26 | **2.01** | 1.67 |
| Probable tellurium resistance protein, TerD family | *bmd_2687* |  | 1.14 | **2.03** | **1.80** |
| 2',3'-cyclic-nucleotide 2'-phosphodiesterase | *bmd_2688* | *yfkN* | -1.19 | **-2.48** | **-2.39** |
| Cold shock protein | *bmd_2698* | *cspC* | -1.16 | -1.68 | 1.13 |
| Conserved hypothetical protein | *bmd_2710* |  | **-2.38** | **-6.73** | **-4.45** |
| Hypothetical protein | *bmd_2714* |  | -1.55 | 1.52 | **1.78** |
| Hypothetical protein | *bmd_2753* |  | -1.37 | 1.59 | **2.14** |
| Conserved hypothetical protein | *bmd_2757* |  | -1.32 | **-2.01** | -1.35 |
| Cold shock protein | *bmd_2791* | *cspC* | -1.23 | 1.39 | **2.16** |
| Hypothetical protein | *bmd_2822* |  | -1.33 | **-1.79** | -1.32 |
| Peptidase M48 | *bmd_2826* |  | -1.01 | **2.30** | 1.08 |
| Conserved hypothetical protein | *bmd_2897* |  | -1.48 | **2.60** | **4.50** |
| Conserved hypothetical protein | *bmd_2910* |  | 1.16 | **-1.88** | **-1.85** |
| Monoacylglycerol lipase | *bmd_2911* |  | 1.67 | -1.49 | **-2.11** |
| 2-oxoglutarate dehydrogenase, E2 component (dihydrolipoamide succinyltransferase) | *bmd_2925* | *odhB* | 1.29 | **3.20** | **3.40** |
| 2-oxoglutarate dehydrogenase, E1 component | *bmd_2926* | *odhA* | 1.19 | **2.43** | **2.36** |
| NAD dependent epimerase/dehydratase family | *bmd_2930* |  | 1.23 | **1.99** | **2.32** |
| Amino acid permease | *bmd_2966* |  | 1.57 | **3.66** | **2.36** |
| Hypothetical protein | *bmd_2977* |  | -1.12 | **-1.79** | -1.32 |
| Endopeptidase | *bmd_2993* |  | -1.66 | **-2.20** | **-1.96** |
| ThiJ/PfpI family protein | *bmd_3006* |  | -1.42 | **3.68** | **4.07** |
| Copper resistance protein | *bmd_3010* | *copC* | 1.00 | 1.02 | **-1.98** |
| ***Cell wall endopeptidase*** | *bmd_3039* | *lytF* | **1.75** | 1.39 | 1.13 |
| Conserved hypothetical protein | *bmd_3050* |  | 1.22 | **-1.99** | **-2.21** |
| Bacterial regulatory protein, arsR family | *bmd_3056* |  | 1.03 | 1.21 | **1.79** |
| Conserved hypothetical protein | *bmd_3061* |  | 1.37 | **2.03** | 1.69 |
| Ribosomal protein S14 | *bmd_3063* |  | 1.13 | **2.28** | 1.74 |
| Transition state regulator, domain protein | *bmd_3064* |  | -1.26 | **-4.53** | **-2.10** |
| DinB family protein | *bmd_3065* |  | -1.23 | **-1.84** | -1.48 |
| Nicotinate-nucleotide--dimethylbenzimidazole phosphoribosyltransferase | *bmd_3069* | *cobT* | -1.22 | **-2.13** | **-1.79** |
| Hypothetical protein | *bmd_3082* |  | 1.22 | **2.60** | **2.77** |
| B12 binding domain protein | *bmd_3083* |  | 1.27 | **2.62** | **2.63** |
| Conserved hypothetical protein | *bmd_3090* |  | -1.65 | 1.58 | **1.95** |
| Malate dehydrogenase | *bmd_3115* |  | 1.28 | **2.31** | 1.48 |
| Putative aminoglycoside N3'-acetyltransferase | *bmd_3116* |  | 1.33 | **3.07** | **1.90** |
| Drug resistance MFS transporter, drug:H+ antiporter-1 (14 Spanner) (DHA2) family | *bmd_3125* |  | -1.32 | **-1.88** | -1.47 |
| Transcriptional regulator, MarR family | *bmd_3126* |  | -1.29 | **-1.87** | -1.49 |
| UDP-glucuronosyltransferase, macrolide glycosyltransferase Family | *bmd_3136* |  | 1.27 | **2.10** | 1.39 |
| Hypothetical protein | *bmd_3145* |  | -1.52 | **2.41** | **2.05** |
| Hypothetical protein | *bmd_3147* |  | -1.55 | **-2.58** | **-1.99** |
| Aminoglycoside N(6')-acetyltransferase, GNAT family | *bmd_3149* |  | 1.26 | **1.92** | **2.10** |
| Conserved hypothetical protein | *bmd_3167* |  | -1.21 | **2.83** | **2.99** |
| LPXTG-motif cell wall anchor domain protein | *bmd_3174* |  | **-1.84** | **-4.06** | **-2.41** |
| Sortase family protein | *bmd_3175* |  | -1.49 | **-2.14** | -1.62 |
| Putative membrane protein | *bmd_3179* |  | -1.24 | **-3.03** | **-2.32** |
| Small multidrug resistance (SMR) family protein | *bmd_3202* |  | -1.36 | **-1.79** | -1.49 |
| Cold shock protein | *bmd_3204* | *cspB* | -1.21 | **-1.84** | -1.38 |
| Manganese catalase | *bmd_3215* |  | 1.01 | **4.14** | **4.20** |
| Putative ferrichrome ABC transporter, ferrichrome-binding protein | *bmd_3216* | *yclQ* | 1.04 | **3.16** | 1.64 |
| Putative ferrichrome ABC transporter, ATP-binding protein | *bmd_3217* | *yclP* | 1.04 | **2.99** | 1.65 |
| Putative ferrichrome ABC transporter, permease protein | *bmd_3218* | *yclO* | 1.00 | **2.45** | 1.26 |
| Putative ferrichrome ABC transporter, permease protein | *bmd_3219* | *yclN* | -1.04 | **3.05** | 1.61 |
| Hypothetical protein | *bmd_3222* |  | -1.12 | **2.95** | **2.75** |
| Hypothetical protein | *bmd_3242* |  | **-1.89** | 1.23 | **2.05** |
| Hypothetical protein | *bmd_3262* |  | -1.60 | **-2.85** | **-1.80** |
| Conserved hypothetical protein | *bmd_3307* |  | -1.01 | **1.88** | 1.57 |
| Conserved hypothetical protein | *bmd_3373* |  | 1.06 | **2.25** | 1.28 |
| Cold shock protein | *bmd_3404* | *cspC* | 1.22 | 1.35 | **1.76** |
| Osmoprotectant transporter gene ousA | *bmd_3405* |  | 1.53 | **3.29** | **2.39** |
| Threonine synthase | *bmd_3406* | *thrC* | 1.48 | -1.67 | **-2.44** |
| Proton/ glutamate symport protein | *bmd_3407* |  | 1.41 | **-2.43** | **-2.19** |
| Hypothetical protein | *bmd_3408* |  | 1.34 | **-2.39** | **-2.61** |
| Sulfate transporter family protein | *bmd_3416* |  | -1.04 | 1.72 | **1.86** |
| ***Two-component response regulator*** | *bmd_3443* |  | **2.00** | -1.08 | -1.30 |
| ***Putative membrane protein*** | *bmd_3444* |  | **2.62** | -1.03 | -1.21 |
| ***Putative membrane protein*** | *bmd_3445* |  | **2.41** | 1.02 | -1.18 |
| ***Putative ABC transporter, ATP-binding protein*** | *bmd_3446* |  | **2.69** | 1.06 | -1.16 |
| Positive regulator of sigma-B activity | *bmd_3481* | *rsbR* | -1.34 | **-2.28** | **-1.87** |
| Hypothetical protein | *bmd_3488* |  | -1.23 | **2.58** | **2.26** |
| Hypothetical protein | *bmd_3489* |  | -1.18 | **2.39** | **2.19** |
| Conserved hypothetical protein | *bmd_3492* |  | -1.29 | **3.73** | **2.52** |
|  |  |  |  |  |  |
|  |  |  |  |  |  |
|  |  |  |  |  |  |
|  |  |  |  |  |  |
|  |  |  |  |  |  |
| **Gene product** | **Gene ID** | **Gene symbol** | **0.6 M** | **1.2 M** | **1.8 M** |
| Oxidoreductase, short chain dehydrogenase/reductase family protein | *bmd_3493* |  | -1.15 | **4.50** | **3.22** |
| Conserved hypothetical protein | *bmd_3494* |  | -1.15 | **1.96** | **2.16** |
| 5-methyltetrahydropteroyltriglutamate--homocysteine S-methyltransferase | *bmd_3527* | *metE* | -1.01 | -1.05 | **2.48** |
| Putative protease, NlpC/P60 family | *bmd_3550* |  | 1.64 | 1.34 | **1.83** |
| 8-amino-7-oxononanoate synthase | *bmd_3693* | *bioF* | 1.59 | **1.88** | 1.31 |
| Conserved hypothetical protein | *bmd_3711* |  | -1.36 | **2.11** | 1.63 |
| CAAX amino terminal protease family protein | *bmd_3728* |  | 1.07 | **3.58** | 1.53 |
| Hypothetical protein | *bmd_3729* |  | -1.02 | **3.10** | 1.53 |
| Conserved hypothetical protein | *bmd_3744* |  | -1.23 | 1.69 | **1.81** |
| Malate dehydrogenase | *bmd_3745* |  | 1.00 | **1.84** | **2.18** |
| Oxidoreductase molybdopterin binding domain protein | *bmd_3784* |  | -1.13 | **-2.36** | -1.73 |
| Conserved hypothetical protein | *bmd_3787* |  | 1.20 | **3.32** | **2.67** |
| Putative 1-pyrroline-5-carboxylate dehydrogenase | *bmd_3813* | *putC* | **4.38** | **5.58** | **3.49** |
| Proline oxidase | *bmd_3814* | *putB* | **1.80** | **1.83** | **1.84** |
| Hypothetical protein | *bmd_3855* |  | -1.21 | 1.06 | **1.96** |
| Hypothetical protein | *bmd_3856* |  | -1.02 | **2.00** | 1.74 |
| Sporulation-specific extracellular nuclease | *bmd_3867* | *nucB* | -1.21 | **1.85** | 1.57 |
| Putative lipoprotein | *bmd_3898* |  | -1.46 | **-3.16** | -1.55 |
| Putative serine proteinase | *bmd_3899* |  | -1.33 | **-2.99** | -1.66 |
| Hypothetical protein | *bmd_3908* |  | 1.14 | 1.54 | **1.79** |
| NADH-dependent flavin oxidoreductase | *bmd_3933* |  | -1.39 | **-2.07** | -1.56 |
| GABA permease (4-amino butyrate transport carrier) | *bmd_3936* |  | -1.24 | **-2.11** | -1.40 |
| NAD dependent epimerase/dehydratase family | *bmd_3943* |  | -1.23 | **1.95** | **1.99** |
| Endopeptidase | *bmd_3978* | *lytF* | **-1.95** | **-3.73** | **-2.32** |
| Conserved hypothetical protein | *bmd_3982* |  | 1.21 | **1.85** | 1.74 |
| Hypothetical protein | *bmd_4014* |  | -1.10 | **1.77** | 1.30 |
| Sporulation-control protein Spo0M | *bmd_4015* | *spo0M* | -1.12 | **2.06** | 1.09 |
| Serine/threonine protein kinase | *bmd_4016* | *prkC* | -1.21 | **2.13** | 1.15 |
| Hypothetical protein | *bmd_4017* |  | -1.20 | **1.78** | 1.12 |
| D-amino acid aminotransferase | *bmd_4023* | *dat* | 1.29 | **2.64** | 1.72 |
| Conserved hypothetical protein | *bmd_4042* |  | -1.48 | **-2.20** | -1.53 |
| Siderophore biosynthesis protein | *bmd_4048* |  | 1.01 | **9.99** | **2.62** |
| Transporter (Major facilitator Superfamily) | *bmd_4049* |  | -1.01 | **13.74** | **3.14** |
| Putative L-lysine 6-monooxygenase (NADPH) | *bmd_4050* |  | 1.00 | **10.56** | **3.05** |
| Siderophore biosynthesis protein | *bmd_4051* |  | 1.04 | **13.09** | **3.86** |
| Siderophore biosynthesis protein | *bmd_4052* |  | 1.07 | **15.03** | **4.11** |
| L-2,4-diaminobutyrate decarboxylase | *bmd_4053* |  | 1.14 | **19.70** | **4.93** |
| 2,4-diaminobutyrate 4-transaminase | *bmd_4054* |  | 1.21 | **16.56** | **4.75** |
| Putative membrane protein | *bmd_4090* |  | -1.33 | **-2.41** | -1.59 |
| 2-oxoglutarate ferredoxin oxidoreductase subunit beta | *bmd_4100* |  | -1.35 | **-1.85** | -1.50 |
| 2-oxoglutarate ferredoxin oxidoreductase subunit alpha | *bmd_4101* |  | -1.28 | **-1.80** | -1.72 |
| Peptidase, M16 family protein | *bmd_4113* |  | 1.19 | **2.39** | 1.57 |
| Putative Zn-protease | *bmd_4114* |  | 1.05 | **1.77** | 1.31 |
| Polynucleotide phosphorylase | *bmd_4132* | *pnp* | -1.07 | -1.56 | **-1.93** |
| Flagellar motor switch protein FliN | *bmd_4168* | *fliN* | 1.14 | **1.89** | 1.53 |
| Flagellar motor switch protein FliM | *bmd_4169* | *fliM* | 1.15 | **1.91** | 1.62 |
| Flagellar basal body-associated protein FliL | *bmd_4170* | *fliL* | 1.10 | **2.03** | 1.54 |
| Flagellar protein FlbD | *bmd_4171* | *flbD* | 1.25 | **1.88** | 1.50 |
| Flagellar hook protein FlgE | *bmd_4172* | *flgE* | 1.16 | **1.83** | 1.55 |
| Flagellar hook-length control protein | *bmd_4174* | *flik* | 1.15 | **2.19** | 1.75 |
| Conserved hypothetical protein | *bmd_4175* |  | 1.15 | **1.97** | 1.69 |
| Flagellar export protein FliJ | *bmd_4176* | *fliJ* | 1.20 | **2.00** | **1.78** |
| Flagellum-specific ATP synthase | *bmd_4177* | *fliI* | 1.24 | **2.10** | 1.69 |
| Flagellar assembly protein FliH | *bmd_4178* | *fliH* | 1.13 | **1.79** | 1.60 |
| Dihydroorotate dehydrogenase, electron transfer subunit | *bmd_4239* | *pyrK* | 1.17 | -1.46 | **-1.85** |
| Carbamoyl-phosphate synthase, large subunit | *bmd_4240* | *pyrAB* | 1.14 | -1.52 | **-2.17** |
| Carbamoyl-phosphate synthase, small subunit | *bmd_4241* | *pyrAA* | 1.20 | -1.55 | **-2.27** |
| Dihydroorotase | *bmd_4242* | *pyrC* | 1.21 | -1.61 | **-1.89** |
| Uracil permease | *bmd_4244* | *pyrP* | -1.02 | **-1.95** | **-1.94** |
| Uracil phosphoribosyl transferase/pyrimidine operon regulatory protein | *bmd_4245* | *pyrR* | -1.17 | **-2.25** | -1.51 |
| Nucleoside diphosphate kinase | *bmd_4314* | *ndk* | 1.34 | **2.17** | **1.86** |
| 1-acyl-sn-glycerol-3-phosphate acyltransferase | *bmd_4331* | *plsC* | -1.26 | -1.73 | **-1.78** |
| FMN permease | *bmd_4350* | *fmnP* | -1.25 | **-2.14** | -1.43 |
| Protein of unknown function (DUF1002) | *bmd_4368* |  | -1.73 | -1.43 | **-1.81** |
| Diaminopimelate decarboxylase | *bmd_4369* | *lysA* | 1.18 | 1.60 | **1.83** |
| Oxidoreductase, aldo/keto reductase family | *bmd_4389* |  | 1.09 | **1.95** | 1.34 |
| Conserved hypothetical protein | *bmd_4390* |  | 1.09 | 1.65 | **1.87** |
| 3-oxoacid CoA-transferase subunit B | *bmd_4391* | *scoB* | 1.34 | **2.00** | 1.52 |
| 3-oxoacid CoA-transferase subunit A | *bmd_4392* | *scoA* | 1.34 | **1.78** | 1.44 |
| Conserved hypothetical protein | *bmd_4397* |  | -1.18 | **-2.17** | -1.45 |
| Methylmalonate-semialdehyde dehydrogenase | *bmd_4404* | *mmsA* | 1.16 | 1.39 | **1.87** |
| Hypothetical protein | *bmd_4407* |  | 1.23 | 1.58 | **1.77** |
| Arginine ABC transporter, ATP-binding protein ArtM | *bmd_4416* | *artM* | -1.25 | **-1.87** | **-1.90** |
| Arginine ABC transporter, permease protein ArtQ | *bmd_4417* | *artQ* | -1.23 | **-1.82** | **-1.82** |
| Arginine ABC transporter, arginine-binding protein ArtP | *bmd_4418* | *artP* | -1.21 | **-1.88** | **-1.82** |
| Amino acid/peptide transporter (Peptide:H+ symporter) | *bmd_4434* |  | -1.25 | **-3.43** | **-2.78** |
|  |  |  |  |  |  |
|  |  |  |  |  |  |
|  |  |  |  |  |  |
|  |  |  |  |  |  |
| **Gene product** | **Gene ID** | **Gene symbol** | **0.6 M** | **1.2 M** | **1.8 M** |
| Glycine cleavage system T protein | *bmd_4471* | *gcvT* | -1.15 | **-2.25** | -1.21 |
| Conserved hypothetical protein | *bmd_4483* |  | 1.35 | **2.25** | **1.95** |
| 50S ribosomal protein L33 | *bmd_4493* | *rpmG* | -1.22 | **-1.84** | -1.36 |
| Hypothetical protein | *bmd_4496* |  | -1.21 | **-1.83** | -1.24 |
| Conserved hypothetical protein | *bmd_4542* |  | 1.37 | **1.95** | 1.12 |
| Conserved hypothetical protein | *bmd_4543* |  | 1.39 | **2.08** | -1.08 |
| Putative membrane protein | *bmd_4544* |  | 1.31 | **1.96** | -1.06 |
| Conserved hypothetical protein | *bmd_4560* |  | -1.01 | 1.43 | **1.93** |
| Hypothetical protein | *bmd_4598* |  | -1.17 | 1.34 | **1.89** |
| Conserved hypothetical protein | *bmd_4632* |  | -1.22 | **2.60** | **2.83** |
| Phosphate transporter | *bmd_4695* | *pit* | -1.31 | **-1.78** | -1.45 |
| Protein of unknown function (DUF47) | *bmd_4696* |  | -1.35 | **-1.82** | -1.37 |
| Succinate dehydrogenase, iron-sulfur protein | *bmd_4709* | *sdhB* | 1.20 | **1.89** | 1.74 |
| Succinate dehydrogenase, flavoprotein subunit | *bmd_4710* | *sdhA* | 1.26 | **2.04** | **1.99** |
| Succinate dehydrogenase, cytochrome b558 subunit | *bmd_4711* | *sdhC* | 1.25 | **1.80** | **1.77** |
| Aspartate kinase | *bmd_4713* | *lysC* | 1.39 | 1.30 | **2.02** |
| Electron transfer flavoprotein, alpha subunit | *bmd_4716* | *etfA* | 1.48 | **2.69** | **1.75** |
| Electron transfer flavoprotein, beta subunit | *bmd_4717* | *etfB* | 1.34 | **2.25** | 1.37 |
| Malate dehydrogenase, NAD-dependent | *bmd_4754* | *mdh* | 1.47 | **2.10** | **2.01** |
| Isocitrate dehydrogenase, NADP-dependent | *bmd_4755* | *icd* | 1.49 | **2.08** | **2.02** |
| Citrate synthase II | *bmd_4756* | *citZ* | 1.52 | **2.39** | **2.24** |
| ATP-NAD kinase | *bmd_4786* | *ppnK* | 1.28 | **1.75** | **1.82** |
| Acetyl-coenzyme A synthetase | *bmd_4798* | *acsA* | 1.58 | **2.57** | **1.93** |
| Conserved hypothetical protein | *bmd_4807* |  | -1.25 | **2.28** | **2.06** |
| Protein of unknown function (DUF948) | *bmd_4808* |  | -1.33 | **2.22** | **2.15** |
| Aminopeptidase | *bmd_4809* |  | -1.06 | -1.51 | **-1.79** |
| M42 glutamyl aminopeptidase | *bmd_4817* |  | 1.26 | **1.83** | 1.58 |
| Conserved hypothetical protein | *bmd_4849* |  | 1.13 | 1.26 | **1.88** |
| DNA-protecting protein | *bmd_4857* | *dps* | -1.55 | 1.45 | **2.10** |
| 50S ribosomal protein L31 | *bmd_4863* | *rpmE* | -1.06 | **1.85** | 1.23 |
| Flavodoxin | *bmd_4866* | *fldA* | -1.07 | **3.92** | **2.09** |
| Conserved hypothetical protein | *bmd_4867* |  | -1.07 | **2.50** | 1.73 |
| Conserved hypothetical protein | *bmd_4868* |  | -1.03 | **3.68** | **2.52** |
| 2-succinyl-6-hydroxy-2,4-cyclohexadiene-1-carboxylate synthase | *bmd_4876* |  | -1.11 | -1.48 | **-1.95** |
| Metal-dependent phosphohydrolase | *bmd_4944* |  | 1.12 | **2.95** | **2.47** |
| NADH dehydrogenase YutJ | *bmd_4957* | *yutJ* | 1.13 | **2.10** | **2.32** |
| FeS assembly protein SufB | *bmd_4976* | *sufB* | 1.04 | 1.71 | **1.92** |
| SUF system FeS assembly protein | *bmd_4977* | *iscU* | 1.13 | **1.92** | **2.07** |
| Cysteine desulfurase SufS | *bmd_4978* | *sufS* | 1.13 | **1.77** | **2.06** |
| FeS assembly protein SufD | *bmd_4979* | *sufD* | 1.12 | 1.60 | **1.96** |
| FeS assembly ATPase SufC | *bmd_4980* | *sufC* | 1.06 | 1.49 | **1.77** |
| Methionine import ABC transporter, methionine-binding protein MetQ | *bmd_4982* | *metQ* | -1.28 | **-2.68** | **-2.13** |
| Methionine import ABC transporter, permease protein MetP | *bmd_4983* | *metP* | -1.26 | **-2.68** | **-1.99** |
| Methionine import ABC transporter, ATP-binding protein MetN | *bmd_4984* | *metN* | -1.25 | **-2.41** | **-2.19** |
| Conserved hypothetical protein | *bmd_4995* |  | 1.11 | **2.62** | **2.16** |
| Conserved hypothetical protein | *bmd_4996* |  | 1.16 | **2.71** | **2.01** |
| Putative ferrichrome import ABC transporter, ATP-binding protein | *bmd_4997* | *yusV* | 1.11 | **2.95** | **2.04** |
| Putative ferrichrome import ABC transporter, permease protein | *bmd_4998* | *yfhA* | 1.15 | **2.95** | **2.27** |
| Putative ferrichrome import ABC transporter, permease protein | *bmd_4999* | *yfiZ* | 1.01 | **2.53** | **2.00** |
| Putative ferrichrome import ABC transporter, ferrichrome-binding protein | *bmd_5000* | *yfiY* | -1.36 | **3.84** | **1.85** |
| Short chain dehydrogenase | *bmd_5005* |  | -1.31 | **2.00** | 1.63 |
| Major facilitator family transporter | *bmd_5006* | *yceI* | -1.28 | **-2.10** | **-1.79** |
| Phosphoglycerate kinase | *bmd_5037* | *pgk* | -1.07 | -1.27 | **-1.77** |
| Conserved hypothetical protein | *bmd_5040* |  | 1.17 | **1.85** | 1.28 |
| Histidine biosynthesis bifunctional protein HisI | *bmd_5052* | *hisI* | -1.09 | **-2.04** | -1.74 |
| Imidazole glycerol phosphate synthase, cyclase subunit | *bmd_5053* | *hisF* | 1.05 | **-1.79** | -1.63 |
| Phosphoribosylformimino-5-aminoimidazole carboxamide ribotide isomerase | *bmd_5054* | *hisA* | -1.01 | **-1.87** | **-1.97** |
| Imidazole glycerol phosphate synthase, glutamine amidotransferase subunit | *bmd_5055* | *hisH* | -1.04 | **-1.99** | -1.67 |
| Imidazoleglycerol-phosphate dehydratase | *bmd_5056* | *hisB* | -1.04 | **-1.95** | -1.75 |
| Histidinol dehydrogenase | *bmd_5057* | *hisD* | -1.04 | **-1.97** | **-1.93** |
| ATP phosphoribosyltransferase, catalytic subunit | *bmd_5058* | *hisG* | -1.06 | **-2.04** | **-1.77** |
| ATP phosphoribosyltransferase, regulatory subunit | *bmd_5059* | *hisZ* | -1.04 | **-2.03** | -1.67 |
| Integral membrane protein | *bmd_5064* |  | -1.21 | **2.51** | 1.39 |
| Regulator (stress mediated) | *bmd_5065* |  | -1.13 | **2.62** | 1.28 |
| Conserved hypothetical protein | *bmd_5066* |  | -1.27 | **2.41** | 1.35 |
| Sigma-54 dependent transcriptional regulator | *bmd_5070* |  | 1.36 | **2.03** | 1.49 |
| Proline dehydrogenase | *bmd_5071* |  | 1.21 | **1.93** | **1.95** |
| Sigma 54 modulation protein / S30EA ribosomal protein | *bmd_5086* |  | 1.19 | **3.61** | **3.62** |
| Sodium:solute symporter family protein | *bmd_5092* |  | **1.92** | **3.39** | 1.68 |
| Betaine aldehyde dehydrogenase | *bmd_5093* | *gbsA* | 1.74 | **3.39** | **2.25** |
| Alcohol dehydrogenase | *bmd_5094* | *gbsB* | 1.59 | **2.99** | **2.17** |
| Flotillin-like protein | *bmd_5114* |  | 1.08 | **2.22** | 1.27 |
| Endopeptidase LytF | *bmd_5120* | *lytF* | -1.38 | **-2.07** | **-2.02** |
| Serine hydroxymethyltransferase | *bmd_5146* | *glyA* | 1.04 | **-1.91** | -1.65 |
| Thymidine kinase | *bmd_5156* | *tdk* | -1.18 | **-2.04** | **-1.89** |
| 50S ribosomal protein L31 | *bmd_5157* | *rpmE* | -1.09 | **-1.83** | -1.45 |
|  |  |  |  |  |  |
|  |  |  |  |  |  |
|  |  |  |  |  |  |
|  |  |  |  |  |  |
| **Gene product** | **Gene ID** | **Gene symbol** | **0.6 M** | **1.2 M** | **1.8 M** |
| UDP-N-acetylglucosamine 1-carboxyvinyltransferase 2 | *bmd_5159* | *murAB* | 1.43 | **2.38** | 1.49 |
| Transaldolase | *bmd_5160* | *tal* | 1.48 | **2.51** | **1.97** |
| CTP synthase | *bmd_5164* | *pyrG* | -1.29 | **-1.75** | **-1.93** |
| Agmatinase | *bmd_5177* | *speB* | -1.67 | **-2.17** | -1.74 |
| Protein of unknown function (UPF0447) | *bmd_5189* |  | 1.19 | **1.75** | 1.47 |
| Transcriptional regulator, LacI family | *bmd_5220* |  | 1.05 | **1.80** | 1.63 |
| Esterase | *bmd_5221* |  | 1.12 | **2.23** | **1.86** |
| Arabinose-proton symporter | *bmd_5222* | *araE* | 1.47 | **2.13** | 1.69 |
| Glutamate-5-semialdehyde dehydrogenase | *bmd_5223* | *proA* | -1.55 | **-3.18** | **-2.69** |
| Glutamate 5-kinase | *bmd_5224* | *proB* | -1.54 | **-3.25** | **-2.49** |

**Supplementary Table 2: Modification of intracellular protein concentrations in *B. megaterium* DSM319 grown with 0.6, 1.2 and 1.8 M NaCl, respectively.** Data are given as fold change of protein concentrations compared to their values in cells grown without additional NaCl. They were obtained from LC-IMS^e^-measurements carried out using four biological replicates for each cultivation condition. Only proteins that were identified in at least 2 out of 3 technical replicates and 2 out of 4 biological replicates were considered for analysis. Only those whose concentration was at least 1.75-fold up- (**red**) or down-regulated (**blue**) at 0.6, 1.2 and/or 1.8 M NaCl were considered as significantly regulated.

| **Protein function** | **ID** | **Protein Symbol** | **0.6 M** | **1.2 M** | **1.8 M** |
| --- | --- | --- | --- | --- | --- |
| Chromosomal replication initiator protein DnaA | BMD_0001 | DnaA | -1.01 | -1.42 | **1.86** |
| Conserved hypothetical protein | BMD_0003 |  | -1.48 | -1.36 | **-1.92** |
| Recombination protein RecR | BMD_0026 | RecR | 1.12 | **1.87** | **2.65** |
| Dimethyladenosine transferase | BMD_0058 | KsgA | 1.39 | 1.11 | **1.79** |
| 50S ribosomal protein L25/general stress protein Ctc | BMD_0069 | Ctc | **3.21** | **6.46** | **10.73** |
| Transcription-repair coupling factor | BMD_0072 | Mfd | -1.05 | -1.05 | **-1.92** |
| Heat shock - Cstr acitivity | BMD_0104 | McsB | -1.01 | 1.48 | **3.19** |
| 23S rRNA methyltransferase | BMD_0115 | RlmB | **-2.17** | -1.32 | -1.39 |
| 50S ribosomal protein L1 | BMD_0122 | RplA | -1.26 | **-1.77** | -1.48 |
| 30S ribosomal protein S7 | BMD_0130 | RpsG | -1.30 | -1.29 | **-2.00** |
| 50S ribosomal protein L23 | BMD_0136 | RplW | **-2.17** | **-2.07** | **-2.05** |
| 30S ribosomal protein S19 | BMD_0138 | RpsS | -1.21 | -1.63 | **-2.12** |
| 50S ribosomal protein L22 | BMD_0139 | RplV | **-1.77** | -1.38 | **-2.07** |
| 50S ribosomal protein L16 | BMD_0141 | RplP | **-2.07** | -1.73 | -1.33 |
| 50S ribosomal protein L29 | BMD_0142 | RpmC | -1.49 | -1.46 | **-2.00** |
| 50S ribosomal protein L24 | BMD_0145 | RplX | -1.03 | -1.23 | **-2.28** |
| 30S ribosomal protein S14 | BMD_0147 | RpsN | -1.62 | **-1.87** | **-2.24** |
| 30S ribosomal protein S5 | BMD_0151 | RpsE | -1.39 | -1.29 | **-1.94** |
| 50S ribosomal protein L15 | BMD_0153 | RplO | -1.11 | -1.25 | **-1.93** |
| Translation initiation factor IF-1 | BMD_0157 | InfA | -1.35 | -1.34 | **-2.36** |
| 50S ribosomal protein L13 | BMD_0166 | RplM | -1.34 | -1.62 | **-1.92** |
| Glucosamine--fructose-6-phosphate aminotransferase, isomerizing | BMD_0192 | GlmS | -1.37 | **-2.02** | **-2.34** |
| D-alanine-D-alanine ligase | BMD_0213 | Ddl | -1.51 | -1.53 | **-1.97** |
| ATP-dependent RNA helicase | BMD_0215 |  | -1.14 | -1.28 | **-1.93** |
| Anti-sigma B factor antagonist | BMD_0227 | RsbV | -1.42 | 1.29 | **2.68** |
| RNA polymerase sigma-B factor | BMD_0229 | SigB | 1.10 | 1.11 | **2.90** |
| S1 RNA binding domain protein | BMD_0231 |  | 1.09 | 1.52 | **2.94** |
| Putative Redox-sensing transcriptional repressor rex | BMD_0255 |  | 1.51 | **2.10** | 1.68 |
| 10 kDa chaperonin | BMD_0260 | GroES | -1.00 | 1.10 | **1.76** |
| GMP synthase [glutamine-hydrolyzing] | BMD_0265 |  | 1.05 | -1.28 | **-2.48** |
| Phosphoribosylaminoimidazole carboxylase, catalytic subunit | BMD_0271 | PurE | 1.01 | **-2.49** | **-4.28** |
| Phosphoribosylaminoimidazole carboxylase, ATPase subunit | BMD_0272 | PurK | 1.21 | **-1.88** | **-4.34** |
| Adenylosuccinate lyase | BMD_0273 | PurB | -1.00 | **-1.94** | **-3.75** |
| Phosphoribosylaminoimidazole-succinocarboxamide synthase | BMD_0274 | PurC | 1.04 | **-1.85** | **-3.34** |
| Phosphoribosylformylglycinamidine synthase, purS protein | BMD_0275 | PurS | 1.34 | -1.52 | **-4.00** |
| Phosphoribosylformylglycinamidine synthase I | BMD_0276 | PurQ | 1.07 | **-2.12** | **-3.84** |
| Phosphoribosylformylglycinamidine synthase II | BMD_0277 | PurL | -1.06 | -1.63 | **-2.54** |
| Amidophosphoribosyltransferase | BMD_0278 | PurF | 1.04 | **-2.05** | **-5.24** |
| Phosphoribosylformylglycinamidine cyclo-ligase | BMD_0279 | PurM | -1.03 | **-2.29** | **-6.67** |
| Bifunctional purine biosynthesis protein PurH | BMD_0281 | PurH | 1.08 | **-1.87** | **-3.44** |
| Phosphoribosylamine--glycine ligase | BMD_0282 | PurD | 1.11 | **-3.05** | **-8.83** |
| Methionine aminopeptidase, type I | BMD_0304 | Map | 1.26 | 1.36 | **3.19** |
| Intracellular protease, PfpI family | BMD_0331 |  | **1.93** | **2.25** | **3.28** |
| Putative efflux ABC transporter, ATP-binding protein | BMD_0361 | YfmM | -1.28 | **-1.77** | **-3.35** |
| Intracellular protease, PfpI family | BMD_0368 |  | 1.17 | 1.60 | **7.51** |
| Conserved hypothetical protein | BMD_0371 |  | -1.23 | -1.48 | **-2.24** |
| Conserved hypothetical protein | BMD_0401 |  | **-2.88** | -1.51 | -1.12 |
| Glutamate-1-semialdehyde-2,1-aminomutase | BMD_0411 | GsaB | **1.87** | **2.25** | **2.12** |
| Peroxide operon regulator | BMD_0417 | PerR | 1.37 | **2.57** | **4.64** |
| Conserved hypothetical protein | BMD_0419 |  | 1.12 | -1.40 | **-1.81** |
| Conserved hypothetical protein | BMD_0440 |  | 1.36 | **2.80** | **2.59** |
| RNA methyltransferase, TrmH family, group 2 | BMD_0443 |  | -1.59 | **-1.76** | -1.08 |
| Proton/sodium-glutamate symport protein | BMD_0453 |  | 1.48 | 1.71 | **1.89** |
| Biotin synthase | BMD_0460 | BioB | 1.10 | **1.85** | **2.11** |
| Putative exported cell wall-binding protein | BMD_0478 | YocH | **2.50** | **11.03** | **19.44** |
| Cell division protein FtsZ | BMD_0511 | FtsZ | **1.89** | **2.67** | **3.09** |
| Glycerol kinase | BMD_0534 | GlpK | 1.33 | 1.19 | **1.83** |
| Putative quinone oxidoreductase, YhdH/YhfP family | BMD_0543 |  | 1.30 | 1.32 | **3.02** |
| L-cystine import ABC transporter, L-cystine-binding protein TcyA | BMD_0547 | TcyA | **-2.12** | **-4.49** | **-2.37** |
| Thiamine biosynthesis protein ThiS | BMD_0553 | ThiS | -1.49 | **-3.38** | **-2.89** |
| Phosphomethylpyrimidine kinase | BMD_0556 | ThiD | 1.70 | **1.75** | **1.81** |
| Conserved hypothetical protein | BMD_0570 |  | 1.58 | **1.78** | **1.81** |
| DNA-binding protein HU | BMD_0576 |  | -1.63 | **-1.90** | -1.23 |
| Protease production transcriptional regulator Hpr | BMD_0585 | Hpr | -1.03 | **-1.92** | **-2.28** |
|  |  |  |  |  |  |
| **Protein function** | **ID** | **Protein Symbol** | **0.6 M** | **1.2 M** | **1.8 M** |
| Monooxygenase | BMD_0599 |  | 1.45 | **2.16** | **2.39** |
| Ferrochelatase | BMD_0602 | HemH | -1.25 | -1.73 | **-4.53** |
| Nuclease SbcCD, C subunit | BMD_0645 | SbcC | **-2.25** | **-2.12** | **-5.11** |
| Conserved hypothetical protein | BMD_0668 |  | 1.29 | 1.54 | **2.10** |
| Conserved hypothetical protein | BMD_0669 |  | 1.68 | 1.32 | **2.81** |
| N-acetyl-gamma-glutamyl-phosphate reductase | BMD_0678 | ArgC | -1.42 | **-2.15** | **-2.27** |
| Arginine biosynthesis bifunctional protein ArgJ | BMD_0679 | ArgJ | -1.58 | **-2.33** | **-4.02** |
| Acetylglutamate kinase | BMD_0680 | ArgB | -1.10 | -1.73 | **-2.75** |
| Acetylornithine aminotransferase | BMD_0681 | ArgD | -1.05 | -1.48 | **-1.92** |
| Carbamoyl-phosphate synthase, small subunit | BMD_0682 | CarA | -1.41 | **-2.12** | **-1.93** |
| Carbamoyl-phosphate synthase, large subunit | BMD_0683 | CarB | -1.39 | **-2.01** | -1.67 |
| ATP-dependent chaperone ClpB | BMD_0687 | ClpB | -1.45 | **-2.15** | -1.24 |
| Oxidoreductase family protein | BMD_0691 |  | -1.14 | -1.61 | **-2.04** |
| 3-oxoacyl-(acyl-carrier-protein) synthase III | BMD_0696 | FabH | -1.21 | -1.74 | **-2.48** |
| Oligopeptide ABC transporter, ATP-binding protein AppF | BMD_0700 | AppF | **-2.46** | 1.23 | 1.40 |
| Oligopeptide ABC transporter, oligopeptide-binding protein AppA | BMD_0701 | AppA | 1.06 | **3.40** | **3.14** |
| Oligopeptide ABC transporter, permease protein OppB | BMD_0707 | OppB | **-2.59** | **-3.02** | **-4.31** |
| Oligopeptide ABC transporter, ATP-binding protein OppF | BMD_0710 | OppF | -1.64 | -1.40 | **-2.06** |
| Gluconate kinase | BMD_0754 | GntK | **2.24** | **3.14** | **2.35** |
| Conserved hypothetical protein | BMD_0757 |  | **2.44** | **2.83** | **2.35** |
| O-acetylhomoserine sulfhydrylase | BMD_0817 |  | 1.34 | 1.29 | **-2.55** |
| Glycine/betaine ABC transporter, ATP-binding protein OpuAA | BMD_0860 | OpuAA | 1.15 | **2.04** | **2.79** |
| Iron(III)-citrate import ABC transporter, iron(III)-citrate-binding protein | BMD_0872 | YfmC | **-2.31** | -1.55 | **-4.20** |
| 3-hexulose-6-phosphate synthase | BMD_0891 | HxlA | -1.11 | 1.53 | **2.80** |
| Hypothetical protein | BMD_0893 |  | 1.03 | **1.75** | **9.93** |
| Oxidoreductase, aldo/keto reductase family | BMD_0912 |  | **1.77** | **2.44** | **3.87** |
| Nitrilotriacetate monooxygenase component B | BMD_0928 |  | 1.13 | 1.24 | **3.53** |
| 4-aminobutyrate aminotransferase | BMD_0945 |  | **-1.88** | **-4.11** | **-8.67** |
| Shikimate kinase | BMD_0952 | AroK | -1.16 | -1.68 | **-2.60** |
| Cob(II)yrinic acid a,c-diamide reductase | BMD_0969 | BluB | **1.77** | **1.79** | 1.19 |
| Oxidoreductase, Gfo/Idh/MocA family (NAD-binding Rossmann fold) | BMD_0989 |  | **1.81** | **2.77** | **1.76** |
| 2-cys peroxiredoxin | BMD_0990 |  | 1.24 | **1.95** | **1.88** |
| Oxidoreductase, aldo/keto reductase family | BMD_1041 |  | **2.22** | **3.22** | **3.01** |
| UDP-glucose 4-epimerase, galE | BMD_1046 | GalE | **1.89** | **1.86** | **2.16** |
| UTP-glucose-1-phosphate uridylyltransferase | BMD_1114 | GalU | -1.52 | **-2.35** | **-3.05** |
| Glycosyl transferase, family 2 | BMD_1117 |  | **-2.22** | **-3.50** | **-8.20** |
| Glycosyl transferase, family 2 | BMD_1118 |  | -1.53 | -1.53 | **-4.03** |
| UDPglucose 6-dehydrogenase | BMD_1122 |  | -1.43 | **-2.42** | **-3.62** |
| Tyrosine-protein kinase capB | BMD_1124 |  | -1.41 | **-1.77** | **-6.42** |
| Tyrosine-protein phosphatase capC | BMD_1125 |  | 1.25 | **1.87** | **1.79** |
| UTP-glucose-1-phosphate uridylyltransferase | BMD_1126 | GalU | 1.17 | 1.70 | **1.83** |
| Fructokinase | BMD_1144 |  | -1.18 | -1.55 | **-2.37** |
| Hypothetical protein | BMD_1166 |  | -1.48 | **-2.06** | **-1.92** |
| Putative membrane protein | BMD_1170 |  | **-6.55** | **-7.80** | **-2.60** |
| Conserved hypothetical protein | BMD_1181 |  | -1.16 | 1.11 | **1.83** |
| Polyhydroxyalkanoic acid inclusion protein PhaP | BMD_1211 | PhaP | 1.32 | **1.81** | **2.15** |
| Polyhydroxyalkanoic acid synthase, PhaR subunit | BMD_1214 | PhaR | 1.66 | **2.24** | **2.22** |
| Polyhydroxyalkanoic acid synthase, PhaC subunit | BMD_1216 | PhaC | 1.35 | 1.32 | **1.81** |
| Methylthioribose-1-phosphate isomerase | BMD_1230 | MtnA | 1.34 | 1.14 | **-1.92** |
| 5-methylthioribose kinase | BMD_1231 | MtnK | 1.02 | **-1.89** | **-2.97** |
| Transaminase | BMD_1233 | MtnE | -1.21 | -1.45 | **-3.29** |
| 2,3-diketo-5-methylthiopentyl-1-phosphate enolase | BMD_1234 | MtnW | 1.13 | -1.26 | **-1.92** |
| YkvE - MarR-type repressor/ transcriptional regulator | BMD_1245 |  | 1.18 | 1.35 | **1.92** |
| ATP-dependent Clp protease, ATP-binding subunit ClpE | BMD_1249 | ClpE | **-2.12** | -1.34 | -1.34 |
| Homocysteine S-methyltransferase/5,10-methylenetetrahydrofolate reductase | BMD_1274 | YitJ | -1.06 | -1.55 | **-1.79** |
| PTS system, glucose-specific IIBC component | BMD_1282 | PtsG | 1.42 | **1.95** | 1.35 |
| Phosphocarrier protein HPr | BMD_1283 | PtsH | -1.15 | 1.15 | **-2.65** |
| Protein of unknown function (DUF1797) | BMD_1307 |  | -1.06 | -1.36 | **-2.56** |
| Conserved hypothetical protein | BMD_1321 |  | 1.30 | 1.23 | **1.95** |
| Conserved hypothetical protein | BMD_1334 |  | 1.59 | 1.21 | **2.05** |
| Inositol monophosphatase | BMD_1338 | SuhB | 1.66 | **2.55** | **5.94** |
| GTPase | BMD_1340 | BipA | **-1.79** | **-2.08** | **-3.93** |
| 3-methyl-2-oxobutanoate hydroxymethyltransferase | BMD_1371 | PanB | **3.49** | **5.93** | **5.47** |
| Pantoate--beta-alanine ligase | BMD_1372 | PanC | **2.53** | **2.65** | **1.91** |
| Aspartate aminotransferase | BMD_1378 | AspB | -1.03 | -1.27 | **-2.68** |
| Penicillin-binding protein 1A/1B | BMD_1383 | PonA | -1.31 | **-2.10** | **-1.84** |
| Cell division protein | BMD_1414 | GpsB | -1.58 | -1.02 | **2.02** |
| Carboxypeptidase Taq (M32) metallopeptidase | BMD_1431 |  | 1.63 | **2.63** | **3.09** |
| Xanthine phosphoribosyltransferase | BMD_1432 | Xpt | -1.15 | **-3.00** | **-6.05** |
| Putative GTPase | BMD_1440 |  | **-1.90** | **-2.30** | **-4.65** |
| Ferrichrome import ABC transporter, ATP-binding protein FhuC | BMD_1510 | FhuC | **2.20** | **5.70** | **12.52** |
| Ferritin-like domain protein | BMD_1538 |  | **1.86** | **2.13** | **7.42** |
| Aldehyde dehydrogenase (NAD) Family Protein | BMD_1546 |  | 1.68 | **4.91** | **32.91** |
| 2,5-diketo-D-gluconic acid reductase A | BMD_1595 |  | -1.12 | **-2.13** | **-3.04** |
| Conserved hypothetical protein | BMD_1629 |  | -1.01 | -1.41 | **-3.20** |
| Nickel import ABC transporter, nickel-binding protein NikA | BMD_1702 | NikA | **-2.49** | **-2.66** | **-2.46** |
|  |  |  |  |  |  |
|  |  |  |  |  |  |
|  |  |  |  |  |  |
|  |  |  |  |  |  |
| **Protein function** | **ID** | **Protein Symbol** | **0.6 M** | **1.2 M** | **1.8 M** |
| 4-hydroxy 2-oxovalerate aldolase | BMD_1715 |  | -1.03 | -1.73 | **-3.23** |
| RecA1 protein | BMD_1726 | RecA1 | 1.01 | -1.06 | **1.97** |
| Conserved hypothetical protein | BMD_1761 |  | -1.10 | -1.20 | **2.52** |
| hut operon positive regulatory protein | BMD_1769 |  | **2.11** | **3.05** | **3.26** |
| Stress response protein YsnF | BMD_1782 | YsnF | -1.29 | -1.18 | **3.48** |
| Conserved hypothetical protein | BMD_1799 |  | **-2.05** | **-1.76** | 1.43 |
| Oligopeptide ABC transporter, oligopeptide-binding protein | BMD_1832 |  | -1.16 | -1.19 | **-3.76** |
| Hypothetical protein | BMD_1845 |  | 1.57 | -1.73 | **-6.95** |
| Aldose 1-epimerase | BMD_1850 | Mro | **1.98** | 1.43 | **1.78** |
| Copper chaperone CopZ (copper-ion-binding protein) | BMD_1895 | CopZ | **2.51** | 1.41 | -1.32 |
| Putative lipoprotein | BMD_1898 |  | **3.82** | **2.19** | **-1.90** |
| Tartrate dehydrogenase/decarboxylase | BMD_1915 | YcsA | **4.51** | **6.73** | **7.09** |
| Transcriptional regulator | BMD_1920 |  | **1.76** | **1.87** | -1.15 |
| PBS lyase HEAT-like repeat family protein | BMD_1943 |  | 1.61 | **2.72** | **2.82** |
| Putative metal ABC transporter, metal-binding protein | BMD_1949 |  | **-2.07** | **-3.74** | **-20.26** |
| Cobalamin synthesis protein/P47K family protein | BMD_1961 |  | **-1.98** | -1.39 | -1.35 |
| Amino acid/peptide transporter (Peptide:H+ symporter) | BMD_2012 | DtpT | **-2.72** | **-4.42** | **-1.85** |
| Malate dehydrogenase | BMD_2037 |  | **2.17** | 1.74 | 1.58 |
| Glutamate synthase, large subunit | BMD_2055 | GltA | -1.10 | -1.55 | **-2.01** |
| Glutamate synthase, small subunit | BMD_2056 | GltB | -1.02 | -1.28 | **-2.12** |
| Aspartate kinase | BMD_2089 | YclM | 1.01 | -1.38 | **-1.90** |
| Conserved hypothetical protein | BMD_2103 |  | -1.21 | **-1.94** | 1.41 |
| NADH-dependent dehydrogenase | BMD_2104 |  | -1.01 | -1.73 | **-2.18** |
| Putative ABC transporter, ATP-binding protein | BMD_2131 | YlmA | **-1.77** | 1.02 | 1.11 |
| Putative nicotinate phosphoribosyltransferase | BMD_2161 |  | -1.01 | 1.01 | **-2.23** |
| NAD+ synthase | BMD_2163 | NadE | 1.37 | **2.01** | **2.50** |
| S1 RNA binding domain protein | BMD_2164 |  | **-1.80** | **-2.66** | **-2.24** |
| General stress protein 17M | BMD_2208 |  | 1.18 | **2.47** | **11.78** |
| Organic hydroperoxide resistance protein | BMD_2231 | OhrB | **-2.19** | **-2.68** | **1.85** |
| Glutamate-5-semialdehyde dehydrogenase | BMD_2245 | ProA* | **8.83** | **14.81** | **19.77** |
| Conserved hypothetical protein | BMD_2246 |  | **3.48** | **3.80** | **2.77** |
| Immune inhibitor A metalloprotease | BMD_2278 | InhA | **-2.20** | **3.72** | **1.91** |
| Fumarate hydratase, class II | BMD_2279 | FumC | -1.17 | **-3.10** | **-3.03** |
| Chaperone protein HtpG | BMD_2385 | HtpG | -1.19 | -1.08 | **-1.92** |
| Conserved hypothetical protein | BMD_2425 |  | **-1.78** | -1.71 | -1.18 |
| NAD dependent epimerase/dehydratase family | BMD_2433 |  | **2.10** | **4.76** | **6.48** |
| Acetyltransferase, GNAT family | BMD_2481 |  | 1.44 | 1.31 | **1.99** |
| Dihydroxy-acid dehydratase | BMD_2497 | IlvD | 1.19 | 1.43 | **2.20** |
| HAD superfamily hydrolase | BMD_2513 |  | 1.26 | -1.08 | **-3.09** |
| Conserved hypothetical protein | BMD_2538 |  | 1.12 | **1.94** | **1.90** |
| Precorrin-4 C11-methyltransferase | BMD_2599 | CbiF | **1.93** | 1.64 | **1.89** |
| CbiET protein | BMD_2601 | CbiET | 1.48 | 1.45 | **1.75** |
| Precorrin-8X methylmutase CbiC | BMD_2603 | CbiC | 1.51 | 1.59 | **1.91** |
| Sirohydrochlorin cobaltochelatase | BMD_2605 | CbiX | 1.21 | 1.66 | **2.52** |
| Precorrin 3 methylase | BMD_2606 | CbiH | 1.38 | 1.75 | **1.91** |
| Malate dehydrogenase | BMD_2620 |  | **3.91** | 1.20 | -1.02 |
| Scyllo-inositol dehydrogenase (NADP+) | BMD_2681 |  | **1.88** | **3.18** | **3.82** |
| Tellurite resistance protein, putative | BMD_2683 |  | 1.46 | 1.49 | **3.05** |
| Tellurium resistance protein terD,TerD family | BMD_2686 |  | 1.37 | **3.21** | **4.99** |
| Probable tellurium resistance protein, TerD family | BMD_2687 |  | -1.10 | **2.08** | **4.08** |
| Aminopeptidase pepS (M29 family) | BMD_2887 | PepS | 1.44 | **1.88** | **2.93** |
| Conserved hypothetical protein | BMD_2910 |  | -1.32 | -1.06 | **-1.77** |
| 2-oxoglutarate dehydrogenase, E2 component (dihydrolipoamide succinyltransferase) | BMD_2925 | OdhB | 1.30 | **1.95** | **2.32** |
| 2-oxoglutarate dehydrogenase, E1 component | BMD_2926 | OdhA | 1.38 | 1.75 | **2.95** |
| Acyl-CoA dehydrogenase | BMD_2954 |  | 1.15 | 1.52 | **2.08** |
| Urease accessory protein UreG | BMD_2986 | UreG | 1.16 | -1.14 | **-2.33** |
| Anthranilate phosphoribosyltransferase | BMD_2992 |  | **2.05** | 1.48 | **2.14** |
| ThiJ/PfpI family protein | BMD_3006 |  | -1.09 | **3.94** | **25.15** |
| Sporulation-control protein Spo0M | BMD_3021 | Spo0M | **-2.15** | -1.73 | -1.71 |
| Cell wall endopeptidase | BMD_3039 | LytF | **9.59** | **13.22** | **23.85** |
| Undecaprenyldiphospho-muramoylpentapeptide beta-N-acetylglucosaminyltransferase | BMD_3054 | MurG | -1.16 | -1.34 | **-2.42** |
| Aspartate ammonia-lyase | BMD_3099 | AnsB | -1.52 | -1.46 | **-3.62** |
| Sulfite reductase (NADPH) hemoprotein, beta-component | BMD_3121 | CysI | 1.01 | -1.32 | **-2.14** |
| Sulfite reductase (NADPH) flavoprotein alpha-component | BMD_3122 |  | -1.02 | 1.00 | **-2.09** |
| UDP-glucuronosyltransferase, macrolide glycosyltransferase Family | BMD_3136 |  | **2.46** | **2.77** | **6.87** |
| Cytochrome aa3 quinol oxidase, subunit III | BMD_3154 | QoxC | -1.22 | -1.07 | **-1.82** |
| Conserved hypothetical protein | BMD_3167 |  | -1.69 | 1.51 | **29.09** |
| Oxidoreductase, zinc-binding dehydrogenase family | BMD_3180 |  | -1.25 | -1.36 | **2.63** |
| Peptide chain release factor 3 | BMD_3203 | PrfC | -1.48 | **-3.59** | **-3.27** |
| Putative ferrichrome ABC transporter, ATP-binding protein | BMD_3217 | YclP | 1.59 | **1.84** | **2.93** |
| Oxidoreductase, aldo/keto reductase family | BMD_3288 |  | **1.76** | **2.06** | **2.76** |
| Aminotransferase family protein | BMD_3340 |  | 1.56 | 1.60 | **2.07** |
| Flavodoxin-like fold family protein | BMD_3384 |  | 1.59 | **2.39** | **3.06** |
| Threonine synthase | BMD_3406 | ThrC | 1.05 | **-1.76** | **-1.98** |
| Hypothetical protein | BMD_3479 |  | -1.07 | -1.68 | **-2.72** |
| Conserved hypothetical protein | BMD_3480 |  | -1.54 | **-1.80** | **-4.59** |
|  |  |  |  |  |  |
|  |  |  |  |  |  |
|  |  |  |  |  |  |
|  |  |  |  |  |  |
| **Protein function** | **ID** | **Protein Symbol** | **0.6 M** | **1.2 M** | **1.8 M** |
| 5-methyltetrahydropteroyltriglutamate--homocysteine S-methyltransferase | BMD_3527 | MetE | 1.74 | **2.25** | **16.50** |
| 8-amino-7-oxononanoate synthase | BMD_3693 | BioF | **1.96** | 1.71 | -1.28 |
| Biotin biosynthesis protein BioC | BMD_3695 | BioC | **1.77** | **1.88** | -1.14 |
| Malate dehydrogenase | BMD_3745 |  | -1.42 | **-2.01** | **2.54** |
| Putative metal-dependent hydrolase | BMD_3772 |  | **1.80** | **1.82** | 1.08 |
| Putative 1-pyrroline-5-carboxylate dehydrogenase | BMD_3813 | PutC | **23.06** | **23.81** | **36.79** |
| Xaa-Pro dipeptidase | BMD_3907 | PepQ | 1.75 | **1.80** | **2.51** |
| Flavodoxin-like fold family protein | BMD_3911 |  | 1.60 | **2.47** | **1.99** |
| Sulfatase | BMD_3930 |  | 1.18 | **3.01** | **2.56** |
| 6-phosphofructokinase | BMD_3977 | PfkA | 1.03 | -1.37 | **-2.01** |
| D-amino acid aminotransferase | BMD_4023 | Dat | **2.71** | **3.83** | **5.32** |
| Siderophore biosynthesis protein | BMD_4048 |  | 1.47 | **4.59** | **5.40** |
| Siderophore biosynthesis protein | BMD_4052 |  | **-2.29** | 1.25 | 1.67 |
| 2,4-diaminobutyrate 4-transaminase | BMD_4054 |  | 1.10 | **3.28** | **5.84** |
| Succinate-semialdehyde dehydrogenase (NADP+) - general stress protein | BMD_4061 |  | 1.37 | **2.47** | **4.27** |
| Succinate-semialdehyde dehydrogenase (NADP+) - GABA utilization | BMD_4061 |  | 1.37 | **2.47** | **4.27** |
| LexA repressor | BMD_4077 | LexA | **1.76** | 1.49 | 1.01 |
| Glutamine synthetase repressor | BMD_4087 | GlnR | 1.25 | **1.82** | **2.73** |
| DNA mismatch repair protein MutS | BMD_4095 | MutS | **-1.93** | -1.20 | -1.01 |
| Amino acid transporter | BMD_4096 |  | -1.40 | -1.23 | **-3.32** |
| 2-oxoglutarate ferredoxin oxidoreductase subunit alpha | BMD_4101 |  | **-2.35** | **-2.13** | **-2.64** |
| RecA2 protein | BMD_4106 | RecA2 | 1.09 | 1.64 | **2.56** |
| Competence/damage-inducible regulator | BMD_4107 | CinA | -1.32 | 1.18 | **2.16** |
| Hypothetical protein | BMD_4109 |  | 1.04 | 1.70 | **1.92** |
| Peptidase, M16 family protein | BMD_4113 |  | 1.38 | **1.82** | 1.60 |
| Putative Zn-protease | BMD_4114 |  | 1.45 | **2.95** | **4.06** |
| Putative ABC transporter, ATP-binding protein | BMD_4117 | YufO | 1.00 | -1.21 | **-2.14** |
| Transcriptional regulator, GntR family | BMD_4119 |  | -1.19 | -1.43 | **-2.79** |
| Polynucleotide phosphorylase | BMD_4132 | Pnp | -1.22 | -1.50 | **-2.10** |
| 30S ribosomal protein S15 | BMD_4133 | RpsO | **-2.10** | **-2.45** | **-2.40** |
| Translation initiation factor IF-2 | BMD_4138 | InfB | -1.08 | 1.46 | **1.86** |
| Conserved hypothetical protein | BMD_4142 |  | -1.07 | 1.19 | **1.97** |
| Prolyl-tRNA synthetase | BMD_4144 | ProS | -1.12 | -1.26 | **-1.87** |
| Uridylate kinase | BMD_4150 | PyrH | -1.33 | **-2.12** | **-2.16** |
| DNA topoisomerase I | BMD_4189 | TopA | 1.20 | 1.68 | **1.96** |
| Ribosome biogenesis GTPase A | BMD_4194 | RbgA | 1.17 | -1.22 | **-2.16** |
| 50S ribosomal protein L19 | BMD_4196 | RplS | -1.49 | -1.47 | **-1.78** |
| Signal recognition particle protein | BMD_4202 | Ffh | 1.17 | 1.74 | **2.24** |
| Putative phosphatase | BMD_4216 |  | 1.34 | **1.75** | **2.11** |
| Ribosome small subunit-dependent GTPase A | BMD_4221 | RsgA | 1.06 | 1.59 | **1.90** |
| Radical SAM enzyme, Cfr family | BMD_4224 |  | 1.54 | 1.45 | **1.88** |
| Methionyl-tRNA formyltransferase | BMD_4226 | Fmt | 1.31 | 1.43 | **3.05** |
| Guanylate kinase | BMD_4231 | Gmk | 1.06 | -1.47 | **-1.95** |
| Orotidine 5'-phosphate decarboxylase | BMD_4237 | PyrF | 1.35 | -1.05 | **-2.52** |
| Dihydroorotate dehydrogenase, catalytic subunit | BMD_4238 | PyrD | 1.02 | -1.04 | **-1.87** |
| Dihydroorotate dehydrogenase, electron transfer subunit | BMD_4239 | PyrK | 1.21 | 1.11 | **-2.13** |
| Carbamoyl-phosphate synthase, large subunit | BMD_4240 | PyrAB | -1.09 | -1.22 | **-2.06** |
| Aspartate carbamoyltransferase | BMD_4243 | PyrB | -1.03 | -1.11 | **-2.02** |
| Pseudouridine synthase | BMD_4246 | RluD | -1.36 | **-2.09** | **-2.57** |
| Cell division machinery factor | BMD_4253 | SepF | -1.23 | **2.05** | 1.62 |
| Cell division protein FtsZ | BMD_4260 | FtsZ | 1.41 | 1.38 | **1.90** |
| UDP-N-acetylenolpyruvoylglucosamine reductase | BMD_4263 | MurB | -1.17 | **-1.77** | **-2.38** |
| UDP-N-acetylmuramoylalanyl-D-glutamate--2,6- diaminopimelate ligase | BMD_4267 | MurE | -1.35 | -1.59 | **-2.59** |
| Acetyltransferase, GNAT family | BMD_4278 |  | -1.12 | -1.39 | **-2.01** |
| 50S ribosomal protein L32 | BMD_4281 | RpmF | 1.08 | -1.19 | **-2.12** |
| Conserved hypothetical protein | BMD_4282 |  | 1.69 | **2.39** | **2.30** |
| Tryptophan synthase, alpha subunit | BMD_4305 | TrpA | **2.27** | **2.60** | 1.68 |
| Tryptophan synthase, beta subunit | BMD_4306 | TrpB | 1.70 | **1.79** | **2.25** |
| Indole-3-glycerol-phosphate synthase | BMD_4308 | TrpC | **1.84** | **2.62** | **2.69** |
| Anthranilate phosphoribosyltransferase | BMD_4309 | TrpD | **-6.90** | **-5.79** | **-7.10** |
| Nucleoside diphosphate kinase | BMD_4314 | Ndk | 1.50 | **1.81** | **2.47** |
| Tryptophan RNA-binding attenuator protein | BMD_4318 | MtrB | **-2.02** | -1.47 | **-2.64** |
| NAD-dependent glycerol-3-phosphate dehydrogenase | BMD_4324 | GpsA | 1.06 | -1.04 | **-1.87** |
| GTP-binding protein EngA | BMD_4325 | EngA | -1.43 | **-1.92** | **-1.86** |
| NAD-specific glutamate dehydrogenase | BMD_4340 | GudB | **-2.57** | **-2.19** | -1.65 |
| D-3-phosphoglycerate dehydrogenase | BMD_4351 | SerA | -1.68 | **-2.05** | **-2.90** |
| Pseudouridine synthase | BMD_4358 | RluB | **-3.07** | **-1.87** | 1.35 |
| Diaminopimelate decarboxylase | BMD_4369 | LysA | 1.23 | **1.78** | **1.87** |
| Nudix hydrolase, YffH family | BMD_4387 |  | 1.63 | **2.84** | **3.33** |
| Oxidoreductase, aldo/keto reductase family | BMD_4389 |  | 1.48 | **2.26** | **3.59** |
| Pyrroline-5-carboxylate reductase | BMD_4400 | ProI | -1.72 | **-1.98** | **-2.07** |
| FAD/FMN-binding oxidoreductase | BMD_4401 |  | 1.22 | 1.10 | **3.03** |
| Arginine ABC transporter, ATP-binding protein ArtM | BMD_4416 | ArtM | 1.04 | **-1.90** | **-2.15** |
| Arginine ABC transporter, permease protein ArtQ | BMD_4417 | ArtQ | -1.39 | **-2.10** | **-2.69** |
| Arginine ABC transporter, arginine-binding protein ArtP | BMD_4418 | ArtP | -1.07 | -1.19 | **-1.86** |
| Conserved hypothetical protein | BMD_4433 |  | 1.01 | -1.54 | **-2.39** |
|  |  |  |  |  |  |
|  |  |  |  |  |  |
|  |  |  |  |  |  |
|  |  |  |  |  |  |
| **Protein function** | **ID** | **Protein Symbol** | **0.6 M** | **1.2 M** | **1.8 M** |
| Protein of unknown function (DUF322) | BMD_4446 |  | -1.23 | 1.09 | **1.84** |
| Translation elongation factor P | BMD_4458 | Efp | 1.42 | 1.12 | **2.11** |
| Proline dipeptidase | BMD_4459 |  | 1.55 | **1.98** | 1.01 |
| Lipoate protein ligase | BMD_4467 |  | **-2.17** | **-3.08** | -1.32 |
| Rhodanese-like domain protein | BMD_4468 |  | 1.08 | -1.50 | **-1.93** |
| Glycine cleavage system T protein | BMD_4471 | GcvT | 1.29 | **1.77** | **3.11** |
| Metallo-beta-lactamase family protein | BMD_4484 |  | 1.40 | **1.88** | 1.19 |
| Conserved hypothetical protein | BMD_4524 |  | 1.50 | **2.39** | **3.93** |
| GTP-binding protein Era | BMD_4534 | Era | **-1.83** | 1.74 | -1.37 |
| Conserved hypothetical protein | BMD_4537 |  | -1.20 | **1.89** | **2.16** |
| GatB/Yqey domain protein | BMD_4545 |  | 1.74 | 1.52 | **2.43** |
| 30S ribosomal protein S21 | BMD_4546 | RpsU | -1.04 | -1.23 | **-4.11** |
| 30S ribosomal protein S20 | BMD_4558 | RpsT | -1.42 | -1.68 | **-2.92** |
| GTP-binding protein | BMD_4571 |  | **-1.94** | **-2.45** | **-1.76** |
| 5'-methylthioadenosine/S-adenosylhomocysteine nucleosidase | BMD_4582 | MtnN | 1.16 | 1.04 | **-1.90** |
| Protein of unknown function (DUF1510) | BMD_4585 |  | **2.18** | **3.39** | **4.09** |
| Transcription elongation factor GreA | BMD_4588 | GreA | 1.42 | **1.77** | 1.70 |
| Cysteine desulfurase | BMD_4605 | IscS | -1.07 | 1.57 | **2.08** |
| Transcriptional regulator of cysteine biosynthesis | BMD_4606 | CymR | 1.28 | 1.23 | **2.69** |
| GTP pyrophosphokinase | BMD_4617 | RelA | 1.63 | **1.93** | **2.41** |
| Queuine tRNA-ribosyltransferase | BMD_4626 | Tgt | -1.10 | -1.38 | **-3.08** |
| Holliday junction DNA helicase RuvA | BMD_4630 | RuvA | -1.16 | **-1.77** | 1.10 |
| 50S ribosomal protein L27 | BMD_4645 | RpmA | -1.27 | **-2.07** | **-3.19** |
| Rod shape-determining protein MreC | BMD_4654 | MreC | -1.47 | -1.22 | **-1.82** |
| Delta-aminolevulinic acid dehydratase | BMD_4668 | HemB | 1.21 | 1.52 | **2.03** |
| 3-isopropylmalate dehydrogenase | BMD_4682 | LeuB | -1.10 | -1.12 | **-1.81** |
| Protein of unknown function (DUF47) | BMD_4696 | BMD_4696 | **-1.88** | **-2.66** | **-2.25** |
| Succinate dehydrogenase, flavoprotein subunit | BMD_4710 | SdhA | 1.23 | 1.59 | **2.32** |
| Succinate dehydrogenase, cytochrome b558 subunit | BMD_4711 | SdhC | 1.19 | 1.31 | **2.19** |
| DNA mismatch repair protein MutS | BMD_4723 | MutS | 1.74 | **3.06** | **2.26** |
| DNA-directed DNA polymerase X | BMD_4724 | PolX | **-1.96** | -1.58 | 1.42 |
| 50S ribosomal protein L20 | BMD_4736 | RplT | -1.65 | **-1.90** | **-1.85** |
| DNA polymerase I | BMD_4750 | PolA | 1.09 | 1.48 | **2.45** |
| Malate dehydrogenase, NAD-dependent | BMD_4754 | Mdh | 1.43 | 1.56 | **1.94** |
| Malate dehydrogenase | BMD_4764 |  | 1.53 | **1.83** | **2.77** |
| Thiamine biosynthesis/tRNA modification protein ThiI | BMD_4789 | ThiI | 1.21 | 1.29 | **2.41** |
| Cysteine desulfurase | BMD_4790 | IscS | -1.35 | -1.08 | **2.17** |
| GAF domain protein | BMD_4794 |  | 1.18 | 1.37 | **1.99** |
| Conserved hypothetical protein | BMD_4807 |  | 1.18 | **2.08** | 1.71 |
| Protein of unknown function (DUF948) | BMD_4808 |  | -1.09 | 1.05 | **2.58** |
| Aminopeptidase | BMD_4809 |  | -1.00 | -1.28 | **-2.10** |
| DNA translocase FtsK (DNA translocase SpoIIIE) | BMD_4812 | FtsK | 1.08 | 1.53 | **1.82** |
| Protein of unknown function (DUF1444) | BMD_4814 |  | 1.33 | **1.82** | **2.45** |
| Thioredoxin | BMD_4815 |  | -1.35 | -1.26 | **2.50** |
| M42 glutamyl aminopeptidase | BMD_4817 |  | **1.81** | **3.16** | **3.69** |
| Putative cysteine synthase A | BMD_4826 | YtkP | **2.40** | **3.84** | **4.15** |
| S-adenosylmethionine synthetase | BMD_4847 | MetK | -1.57 | **-2.06** | **-1.84** |
| Phosphoenolpyruvate carboxykinase (ATP) | BMD_4848 | PckA | 1.19 | **2.22** | **3.70** |
| DNA-protecting protein | BMD_4857 | Dps | -1.21 | 1.13 | **4.49** |
| S-ribosylhomocysteine lyase | BMD_4858 | LuxS | 1.49 | **1.84** | 1.24 |
| Ribonucleoside-diphosphate reductase, beta subunit | BMD_4871 | NrdF | 1.59 | 1.51 | **1.97** |
| Sirohydrochlorin ferrochelatase | BMD_4913 | SirB | **2.03** | 1.49 | 1.28 |
| NADH-dependent butanol dehydrogenase A | BMD_4931 |  | -1.54 | **-2.16** | **-2.05** |
| S1 RNA binding domain-containing protein - general stress protein 13 | BMD_4933 |  | 1.60 | **1.78** | 1.40 |
| Aminotransferase | BMD_4937 | PatB | 1.38 | **2.72** | **2.44** |
| Leucyl aminopeptidase | BMD_4947 | PepA | 1.34 | 1.68 | **2.41** |
| NADH dehydrogenase YutJ | BMD_4957 | YutJ | **2.78** | **4.28** | **9.24** |
| Homoserine kinase | BMD_4960 | ThrB | 1.39 | **1.82** | **1.85** |
| Conserved hypothetical protein | BMD_4968 |  | 1.57 | **1.75** | 1.68 |
| SUF system FeS assembly protein | BMD_4977 | IscU | 1.43 | **1.93** | **2.09** |
| Cysteine desulfurase SufS | BMD_4978 | SufS | 1.73 | **2.14** | **3.26** |
| FeS assembly protein SufD | BMD_4979 | SufD | 1.12 | 1.39 | **1.79** |
| Methionine import ABC transporter, methionine-binding protein MetQ | BMD_4982 | MetQ | -1.42 | **-2.03** | **-3.42** |
| Methionine import ABC transporter, ATP-binding protein MetN | BMD_4984 | MetN | -1.33 | -1.56 | **-2.64** |
| Conserved hypothetical protein | BMD_4989 |  | **-2.04** | 1.17 | -1.56 |
| Putative ferrichrome import ABC transporter, ferrichrome-binding protein | BMD_5000 | YfiY | **-1.91** | 1.12 | **1.88** |
| Phosphoglycerate kinase | BMD_5037 | Pgk | -1.14 | -1.14 | **-1.79** |
| Glyceraldehyde-3-phosphate dehydrogenase, type I | BMD_5038 | Gap | -1.11 | -1.39 | **-1.81** |
| Conserved hypothetical protein | BMD_5046 |  | 1.72 | **2.52** | **4.19** |
| Putative triphosphate pyrophosphate hydrolase | BMD_5049 | YvcI | **1.91** | -1.07 | -1.11 |
| Histidine biosynthesis bifunctional protein HisI | BMD_5052 | HisI | 1.16 | -1.11 | **-2.27** |
| Imidazole glycerol phosphate synthase, cyclase subunit | BMD_5053 | HisF | -1.10 | -1.53 | **-2.62** |
| Phosphoribosylformimino-5-aminoimidazole carboxamide ribotide isomerase | BMD_5054 | HisA | 1.14 | -1.48 | **-2.55** |
| HPr(Ser) kinase/phosphatase | BMD_5063 | HprK | -1.43 | -1.74 | **-1.85** |
| Excinuclease ABC, A subunit | BMD_5068 | UvrA | **2.54** | **2.21** | **2.11** |
| Peptide chain release factor 2 | BMD_5083 | PrfB | -1.28 | -1.70 | **-1.91** |
|  |  |  |  |  |  |
|  |  |  |  |  |  |
|  |  |  |  |  |  |
|  |  |  |  |  |  |
| **Protein function** | **ID** | **Protein Symbol** | **0.6 M** | **1.2 M** | **1.8 M** |
| Preprotein translocase, SecA subunit | BMD_5084 | SecA | -1.07 | 1.14 | **2.06** |
| Sigma 54 modulation protein / S30EA ribosomal protein | BMD_5086 |  | 1.27 | **2.61** | **9.06** |
| UDP-N-acetylglucosamine 1-carboxyvinyltransferase | BMD_5130 | MurA | **-1.85** | -1.61 | 1.24 |
| ATP synthase F1, gamma subunit | BMD_5135 | AtpG | -1.16 | -1.60 | **-2.57** |
| Ribose 5-phosphate isomerase B | BMD_5148 | RpiB | -1.28 | -1.37 | **-2.04** |
| Protein-tyrosine phosphatase | BMD_5149 |  | **9.48** | **3.75** | **26.42** |
| Transaldolase | BMD_5160 | Tal | 1.66 | **2.69** | **3.75** |
| Sporulation initiation phosphotransferase F (response regulator) | BMD_5162 | Spo0F | -1.26 | -1.40 | **-2.09** |
| CTP synthase | BMD_5164 | PyrG | -1.48 | **-2.44** | **-2.60** |
| Agmatinase | BMD_5177 | SpeB | **-2.40** | **-3.06** | **-2.96** |
| Spermidine synthase | BMD_5178 | SpeE | **-2.09** | **-3.22** | **-2.11** |
| 4-oxalocrotonate tautomerase | BMD_5182 |  | **1.98** | **2.17** | -1.00 |
| Protein of unknown function (UPF0447) | BMD_5189 |  | 1.16 | **1.85** | **2.20** |
| 6-phosphogluconate dehydrogenase, decarboxylating | BMD_5197 | Gnd | -1.43 | **-1.82** | **-2.46** |
| Conserved hypothetical protein | BMD_5198 |  | **2.66** | **2.06** | **2.14** |
| Alanine dehydrogenase | BMD_5199 | Ald | 1.59 | **5.62** | **9.70** |
| Phosphomethylpyrimidine kinase | BMD_5201 | ThiD | 1.18 | **1.90** | **3.30** |
| Cof-like hydrolase | BMD_5202 |  | **2.39** | **2.45** | **3.11** |
| Glycosyl transferase, family 2 | BMD_5207 |  | -1.17 | -1.58 | **-1.94** |
| Glutamate-5-semialdehyde dehydrogenase | BMD_5223 | ProA | **-2.33** | **-3.53** | **-9.96** |
| Catalase | BMD_5226 | KatA | -1.14 | **-1.85** | **-3.14** |
| 50S ribosomal protein L9 | BMD_5252 | RplI | 1.06 | 1.31 | **1.98** |
| Single-strand binding protein | BMD_5256 | SsbA | 1.22 | -1.20 | **-1.81** |
| GTP-binding protein EngD | BMD_5258 | EngD | -1.29 | -1.35 | **-2.05** |
| tRNA uridine 5-carboxymethylaminomethyl modification enzyme GidA | BMD_5267 | GidA | 1.32 | 1.61 | **3.50** |
| tRNA modification GTPase TrmE | BMD_5268 | TrmE | -1.17 | 1.09 | **1.76** |

**Supplementary Table 3: Biochemical reaction network used for flux calculation with OpenFlux.** All reactions are listed with their corresponding stoichiometry (rxnEQ), carbon atom transition (cTrans) and type. Reactions marked with an “X” indicate reactions set as free fluxes for the simulation.

| **rxnID** | | **rxnEQ** | | **cTrans** | **Type** |  | |  | |
| --- | --- | --- | --- | --- | --- | --- | --- | --- | --- |
| R01 | GLC_EX = GLC6P | | abcdef = abcdef | | F |  | **PTS** | |  |
| R02 | GLC6P = F6P | | abcdef = abcdef | | F |  | **EMP** | |  |
| R03 | F6P = GLC6P | | abcdef = abcdef | | F |  |  |
| R04 | F6P = F16BP | | abcdef = abcdef | | F |  |  |
| R05 | F16BP = DHAP + G3P | | abcdef = abc + def | | F |  |  |
| R06 | DHAP = G3P | | abc = cba | | F |  |  |
| R07 | GLC6P = P5P + CO2 | | abcdef = bcdef + a | | F | X | **PPP** | |  |
| R08 | P5P + P5P = S7P + G3P | | abcde + fghij = fgabcde + hij | | FR |  |  |
| R09 | S7P + G3P = P5P + P5P | | fgabcde + hij = abcde + fghij | | R | X |  |
| R10 | S7P + G3P = E4P + F6P | | abcdefg + hij = defg + abchij | | FR |  |  |
| R11 | E4P + F6P = S7P + G3P | | defg + abchij = abcdefg + hij | | R | X |  |
| R12 | E4P + P5P = F6P + G3P | | abcd + efghi = efabcd + ghi | | FR |  |  |
| R13 | F6P + G3P = E4P + P5P | | efabcd + ghi = abcd + efghi | | R | X |  |
| R14 | G3P = 3PG | | abc = abc | | FR |  | **EMP** | |  |
| R15 | 3PG = G3P | | abc = abc | | R | X |  |
| R16 | 3PG = PEP | | abc = abc | | F |  |  |
| R17 | PEP = PYR | | abc = abc | | F |  |  |
| R18 | PYR = ACCOA + CO2 | | abc = bc + a | | F |  | **TCA** | |  |
| R19 | ACCOA + OAA = AKG + CO2 | | ab + cdef = fedba + c | | F |  |  |
| R20 | AKG = 0.5 SUC + 0.5 SUC + CO2 | | abcde = 0.5 bcde + 0.5 edcb + a | | F |  |  |
| R21 | SUC = MAL | | abcd = abcd | | F |  |  |
| R22 | MAL = OAA | | abcd = abcd | | F |  |  |
| R23 | PYR + CO2 = OAA | | abc + d = abcd | | F | X | **PEP-PYR-OAA node** | |  |
| R24 | MAL = PYR + CO2 | | abcd = abc + d | | F | X |  |
| R25 | OAA = PEP + CO2 | | abcd = abc + d | | F | X |  |
| R26 | PEP + CO2 = OAA | | abc + d = abcd | | F | X |  |
| R27 | CO2 = CO2_EX | | a = a | | FR |  | **CO2** | |  |
| R28 | CO2_EX = CO2 | | a = a | | R | X |  |
| R29 | AKG = AKG_EX | |  | | B |  | **Organic acids** | |  |
| R30 | PYR = PYR_EX | |  | | B |  |  |
| R31 | SUC = SUC_EX | |  | | B |  |  |
| R32 | PYR = LAC_EX | |  | | B |  |  |
| R33 | ACCOA = ACETAT_EX | |  | | B |  |  |
| R34 | GLC6P = GLC6P_B | |  | | B |  | **Biomass** | |  |
| R35 | F6P = F6P_B | |  | | B |  |  |
| R36 | P5P = P5P_B | |  | | B |  |  |
| R37 | E4P = E4P_B | |  | | B |  |  |
| R38 | G3P = G3P_B | |  | | B |  |  |
| R39 | 3PG = 3PG_B | | abc = abc | | F |  |  |
| R40 | PEP = PEP_B | |  | | B |  |  |
| R41 | PYR = PYR_B | |  | | B |  |  |
| R42 | OAA = OAA_B | |  | | B |  |  |
| R43 | ACCOA = ACCOA_B | |  | | B |  |  |
| R44 | AKG = AKG_B | |  | | B |  |  |
| R45 | MTHF = MTHF_B | |  | | B |  |  |
| R46 | 3PG_B = 3PG_BT | |  | | B |  |  |
| R47 | 0.205 VALX + 0.065 TYRX + 0.098 PHEX + 0.137 SER + 0.434 ALAX + 0.268 GLYX + 0.242 LYSX + 0.158 THR + 0.151 ASPX + 0.343 GLUX = BIOMASS | |  | | B |  |  |
| R48 | 3PG_B = SER | | abc = abc | | F |  | **Amino acids** | |  |
| R49 | SER = GLYX + MTHF | | abc = ab + c | | FR |  |  |
| R50 | GLYX + MTHF = SER | | ab + c = abc | | R | X |  |
| R51 | E4P + PEP = SHKM | | abcd + efg = efgabcd | | S |  |  |
| R52 | SHKM + PEP = CHRM | | abcdefg + hij = abcdefghij | | S |  |  |
| R53 | CHRM = PHEX + CO2 | | abcdefghij = hijbcdefg + a | | S |  |  |
| R54 | CHRM = TYRX + CO2 | | abcdefghij = hijbcdefg + a | | S |  |  |
| R55 | PYR + PYR = VALX + CO2 | | abc + def = abefc + d | | S |  |  |
| R56 | PYR = ALAX | | abc = abc | | S |  |  |
| R57 | OAA + PYR = 0.5 LYSX + 0.5 LYSX + 0.5 CO2 + 0.5 CO2 | | abcd + efg = 0.5 abcdfg + 0.5 efgdcb + 0.5 e + 0.5 a | | S |  |  |
| R58 | OAA = THR | | abcd = abcd | | S |  |  |
| R59 | OAA = ASPX | | abcd = abcd | | S |  |  |
| R60 | AKG = GLUX | | abcde = abcde | | S |  |  |
| R61 | ACCOA + ACCOA = HB | | ab + cd = abcd | | S |  | **PHB** | |  |

**Supplementary Table 4: Comparison between measured and simulated labelling patterns of proteinogenic amino acids and intracellular polyhydroxybutyric acid when labelling data from tracer experiments with 1-^13^C glucose and a mixture of 50 % U-^12^C / 50 % U-^13^C as substrates are used for simulation of metabolic fluxes in cells growing in presence of 0, 0.6 and 1.2 M NaCl.** Model from Supplementary Table 3 was used for simulation with OpenFlux.

|  |  |  | **0 M NaCl** | | **0.6 M NaCl** | | **1.2 M NaCl** | |
| --- | --- | --- | --- | --- | --- | --- | --- | --- |
|  |  |  | **Sim** | **Exp** | **Sim** | **Exp** | **Sim** | **Exp** |
| **1-^13^C labelling** | **Ala 260** | **m+0** | 0.502 | 0.507 | 0.522 | 0.526 | 0.546 | 0.548 |
|  |  | **m+1** | 0.362 | 0.362 | 0.347 | 0.347 | 0.329 | 0.329 |
|  |  | **m+2** | 0.105 | 0.102 | 0.102 | 0.099 | 0.099 | 0.096 |
|  |  | **m+3** | 0.031 | 0.030 | 0.029 | 0.028 | 0.027 | 0.026 |
|  | **Ala 232** | **m+0** | 0.536 | 0.529 | 0.552 | 0.548 | 0.574 | 0.571 |
|  |  | **m+1** | 0.365 | 0.371 | 0.350 | 0.355 | 0.331 | 0.335 |
|  |  | **m+2** | 0.100 | 0.100 | 0.098 | 0.097 | 0.095 | 0.095 |
|  | **Val 288** | **m+0** | 0.334 | 0.333 | 0.359 | 0.357 | 0.391 | 0.390 |
|  |  | **m+1** | 0.404 | 0.406 | 0.397 | 0.398 | 0.385 | 0.386 |
|  |  | **m+2** | 0.190 | 0.191 | 0.178 | 0.179 | 0.163 | 0.164 |
|  |  | **m+3** | 0.056 | 0.055 | 0.052 | 0.052 | 0.048 | 0.047 |
|  |  | **m+4** | 0.014 | 0.013 | 0.012 | 0.012 | 0.011 | 0.011 |
|  |  | **m+5** | 0.002 | 0.002 | 0.002 | 0.002 | 0.002 | 0.002 |
|  | **Val 260** | **m+0** | 0.348 | 0.338 | 0.370 | 0.363 | 0.403 | 0.396 |
|  |  | **m+1** | 0.406 | 0.408 | 0.398 | 0.400 | 0.385 | 0.387 |
|  |  | **m+2** | 0.182 | 0.188 | 0.172 | 0.176 | 0.157 | 0.161 |
|  |  | **m+3** | 0.052 | 0.053 | 0.049 | 0.049 | 0.045 | 0.045 |
|  |  | **m+4** | 0.012 | 0.013 | 0.011 | 0.011 | 0.010 | 0.010 |
|  | **Thr 404** | **m+0** | 0.340 | 0.345 | 0.347 | 0.351 | 0.360 | 0.364 |
|  |  | **m+1** | 0.378 | 0.378 | 0.374 | 0.374 | 0.368 | 0.368 |
|  |  | **m+2** | 0.191 | 0.189 | 0.190 | 0.187 | 0.185 | 0.183 |
|  |  | **m+3** | 0.070 | 0.069 | 0.070 | 0.069 | 0.068 | 0.066 |
|  |  | **m+4** | 0.020 | 0.019 | 0.020 | 0.019 | 0.019 | 0.019 |
|  | **Thr 376** | **m+0** | 0.370 | 0.374 | 0.382 | 0.383 | 0.395 | 0.398 |
|  |  | **m+1** | 0.382 | 0.383 | 0.376 | 0.377 | 0.369 | 0.369 |
|  |  | **m+2** | 0.183 | 0.179 | 0.179 | 0.177 | 0.175 | 0.173 |
|  |  | **m+3** | 0.066 | 0.064 | 0.064 | 0.063 | 0.062 | 0.061 |
|  | **Asp 418** | **m+0** | 0.339 | 0.346 | 0.346 | 0.351 | 0.360 | 0.364 |
|  |  | **m+1** | 0.378 | 0.376 | 0.373 | 0.373 | 0.368 | 0.367 |
|  |  | **m+2** | 0.191 | 0.188 | 0.190 | 0.187 | 0.185 | 0.183 |
|  |  | **m+3** | 0.071 | 0.070 | 0.071 | 0.069 | 0.068 | 0.067 |
|  |  | **m+4** | 0.020 | 0.020 | 0.020 | 0.020 | 0.019 | 0.019 |
|  | **Asp 390** | **m+0** | 0.369 | 0.374 | 0.381 | 0.383 | 0.394 | 0.397 |
|  |  | **m+1** | 0.381 | 0.382 | 0.375 | 0.376 | 0.368 | 0.368 |
|  |  | **m+2** | 0.183 | 0.180 | 0.179 | 0.178 | 0.175 | 0.173 |
|  |  | **m+3** | 0.066 | 0.065 | 0.065 | 0.064 | 0.063 | 0.062 |
|  | **Asp 316** | **m+0** | 0.406 | 0.408 | 0.419 | 0.417 | 0.434 | 0.433 |
|  |  | **m+1** | 0.390 | 0.386 | 0.382 | 0.380 | 0.373 | 0.370 |
|  |  | **m+2** | 0.158 | 0.154 | 0.154 | 0.153 | 0.149 | 0.148 |
|  |  | **m+3** | 0.047 | 0.051 | 0.045 | 0.050 | 0.044 | 0.049 |
|  | **Glu 432** | **m+0** | 0.242 | 0.247 | 0.258 | 0.260 | 0.278 | 0.281 |
|  |  | **m+1** | 0.367 | 0.368 | 0.367 | 0.367 | 0.366 | 0.365 |
|  |  | **m+2** | 0.244 | 0.241 | 0.235 | 0.234 | 0.225 | 0.224 |
|  |  | **m+3** | 0.104 | 0.102 | 0.100 | 0.098 | 0.094 | 0.093 |
|  |  | **m+4** | 0.034 | 0.033 | 0.032 | 0.032 | 0.030 | 0.030 |
|  |  | **m+5** | 0.009 | 0.009 | 0.008 | 0.008 | 0.008 | 0.008 |
| **1-^13^C labelling** | **Glu 330** | **m+0** | 0.331 | 0.323 | 0.346 | 0.343 | 0.377 | 0.371 |
|  |  | **m+1** | 0.402 | 0.402 | 0.395 | 0.396 | 0.385 | 0.386 |
|  |  | **m+2** | 0.193 | 0.199 | 0.187 | 0.189 | 0.173 | 0.176 |
|  |  | **m+3** | 0.059 | 0.061 | 0.058 | 0.059 | 0.053 | 0.054 |
|  |  | **m+4** | 0.014 | 0.015 | 0.014 | 0.015 | 0.013 | 0.013 |
|  | **Ser 390** | **m+0** | 0.440 | 0.448 | 0.460 | 0.465 | 0.480 | 0.482 |
|  |  | **m+1** | 0.365 | 0.363 | 0.353 | 0.351 | 0.339 | 0.338 |
|  |  | **m+2** | 0.144 | 0.141 | 0.140 | 0.138 | 0.136 | 0.135 |
|  |  | **m+3** | 0.051 | 0.049 | 0.048 | 0.047 | 0.045 | 0.045 |
|  | **Ser 362** | **m+0** | 0.475 | 0.476 | 0.491 | 0.493 | 0.510 | 0.512 |
|  |  | **m+1** | 0.379 | 0.380 | 0.367 | 0.366 | 0.352 | 0.352 |
|  |  | **m+2** | 0.145 | 0.144 | 0.142 | 0.141 | 0.138 | 0.137 |
|  | **Ser 288** | **m+0** | 0.515 | 0.515 | 0.532 | 0.534 | 0.554 | 0.554 |
|  |  | **m+1** | 0.373 | 0.374 | 0.359 | 0.358 | 0.341 | 0.341 |
|  |  | **m+2** | 0.111 | 0.111 | 0.108 | 0.108 | 0.104 | 0.105 |
|  | **Phe 234** | **m+0** | 0.325 | 0.334 | 0.354 | 0.360 | 0.390 | 0.391 |
|  |  | **m+1** | 0.413 | 0.411 | 0.410 | 0.405 | 0.400 | 0.396 |
|  |  | **m+2** | 0.199 | 0.192 | 0.181 | 0.178 | 0.163 | 0.163 |
|  |  | **m+3** | 0.052 | 0.048 | 0.045 | 0.044 | 0.039 | 0.040 |
|  |  | **m+4** | 0.010 | 0.010 | 0.008 | 0.009 | 0.007 | 0.008 |
|  |  | **m+5** | 0.001 | 0.002 | 0.001 | 0.002 | 0.001 | 0.002 |
|  |  | **m+6** | 0.000 | 0.001 | 0.000 | 0.000 | 0.000 | 0.000 |
|  |  | **m+7** | 0.000 | 0.000 | 0.000 | 0.000 | 0.000 | 0.000 |
|  |  | **m+8** | 0.000 | 0.000 | 0.000 | 0.000 | 0.000 | 0.001 |
|  | **Phe 302** | **m+0** | 0.724 | 0.732 | 0.730 | 0.731 | 0.722 | 0.729 |
|  |  | **m+1** | 0.200 | 0.194 | 0.195 | 0.194 | 0.201 | 0.196 |
|  |  | **m+2** | 0.076 | 0.074 | 0.075 | 0.074 | 0.077 | 0.075 |
|  | **Gly 246** | **m+0** | 0.754 | 0.759 | 0.760 | 0.759 | 0.762 | 0.758 |
|  |  | **m+1** | 0.175 | 0.171 | 0.169 | 0.171 | 0.168 | 0.171 |
|  |  | **m+2** | 0.071 | 0.070 | 0.070 | 0.070 | 0.070 | 0.070 |
|  | **Gly 218** | **m+0** | 0.829 | 0.828 | 0.829 | 0.828 | 0.829 | 0.827 |
|  |  | **m+1** | 0.171 | 0.172 | 0.171 | 0.172 | 0.171 | 0.173 |
|  |  |  |  |  |  |  |  |  |
|  |  |  |  |  |  |  |  |  |
|  | **Tyr 466** | **m+0** | 0.244 | 0.257 | 0.269 | 0.278 | 0.295 | 0.298 |
|  |  | **m+1** | 0.365 | 0.368 | 0.369 | 0.367 | 0.366 | 0.364 |
|  |  | **m+2** | 0.240 | 0.231 | 0.228 | 0.221 | 0.215 | 0.211 |
|  |  | **m+3** | 0.104 | 0.094 | 0.095 | 0.089 | 0.088 | 0.084 |
|  |  | **m+4** | 0.035 | 0.033 | 0.030 | 0.031 | 0.028 | 0.029 |
|  |  | **m+5** | 0.009 | 0.009 | 0.008 | 0.008 | 0.007 | 0.008 |
|  |  | **m+6** | 0.002 | 0.003 | 0.002 | 0.003 | 0.001 | 0.002 |
|  |  | **m+7** | 0.000 | 0.001 | 0.000 | 0.001 | 0.000 | 0.001 |
|  |  | **m+8** | 0.000 | 0.001 | 0.000 | 0.001 | 0.000 | 0.001 |
|  |  | **m+9** | 0.000 | 0.001 | 0.000 | 0.001 | 0.000 | 0.001 |
|  | **Tyr 302** | **m+0** | 0.724 | 0.732 | 0.730 | 0.732 | 0.722 | 0.731 |
|  |  | **m+1** | 0.200 | 0.193 | 0.195 | 0.194 | 0.201 | 0.195 |
|  |  | **m+2** | 0.076 | 0.074 | 0.075 | 0.074 | 0.077 | 0.075 |
|  | **Lys 431** | **m+0** | 0.230 | 0.240 | 0.244 | 0.254 | 0.265 | 0.274 |
|  |  | **m+1** | 0.364 | 0.363 | 0.364 | 0.362 | 0.364 | 0.362 |
|  |  | **m+2** | 0.250 | 0.245 | 0.242 | 0.238 | 0.232 | 0.227 |
|  |  | **m+3** | 0.109 | 0.105 | 0.105 | 0.102 | 0.098 | 0.095 |
|  |  | **m+4** | 0.036 | 0.035 | 0.035 | 0.033 | 0.032 | 0.031 |
|  |  | **m+5** | 0.009 | 0.009 | 0.009 | 0.009 | 0.008 | 0.008 |
|  |  | **m+6** | 0.002 | 0.003 | 0.002 | 0.002 | 0.002 | 0.002 |
|  | **Lys 329** | **m+0** | 0.269 | 0.272 | 0.287 | 0.288 | 0.309 | 0.310 |
|  |  | **m+1** | 0.390 | 0.383 | 0.388 | 0.382 | 0.386 | 0.377 |
|  |  | **m+2** | 0.232 | 0.234 | 0.222 | 0.224 | 0.210 | 0.213 |
|  |  | **m+3** | 0.083 | 0.083 | 0.078 | 0.079 | 0.073 | 0.074 |
|  |  | **m+4** | 0.022 | 0.023 | 0.021 | 0.022 | 0.019 | 0.020 |
|  |  | **m+5** | 0.004 | 0.005 | 0.004 | 0.005 | 0.004 | 0.005 |
|  | **HB_255** | **m+0** | - | - | 0.370 | 0.364 | 0.402 | 0.399 |
|  |  | **m+1** | - | - | 0.397 | 0.399 | 0.384 | 0.386 |
|  |  | **m+2** | - | - | 0.172 | 0.175 | 0.158 | 0.159 |
|  |  | **m+3** | - | - | 0.050 | 0.050 | 0.046 | 0.046 |
|  |  | **m+4** | - | - | 0.012 | 0.012 | 0.010 | 0.010 |
|  | **HB_233** | **m+0** | - | - | 0.553 | 0.549 | 0.575 | 0.573 |
|  |  | **m+1** | - | - | 0.349 | 0.354 | 0.329 | 0.333 |
|  |  | **m+2** | - | - | 0.098 | 0.097 | 0.095 | 0.094 |
| **50 % U-^12^C / 50% U-^13^C labelling** | **Ala 260** | **m+0** | 0.370 | 0.380 | 0.366 | 0.372 | 0.360 | 0.371 |
|  |  | **m+1** | 0.133 | 0.128 | 0.136 | 0.134 | 0.141 | 0.134 |
|  |  | **m+2** | 0.105 | 0.113 | 0.108 | 0.121 | 0.115 | 0.119 |
|  |  | **m+3** | 0.392 | 0.379 | 0.389 | 0.372 | 0.384 | 0.376 |
|  | **Ala 232** | **m+0** | 0.415 | 0.418 | 0.410 | 0.414 | 0.409 | 0.413 |
|  |  | **m+1** | 0.126 | 0.140 | 0.134 | 0.148 | 0.136 | 0.145 |
|  |  | **m+2** | 0.459 | 0.443 | 0.456 | 0.439 | 0.455 | 0.442 |
|  | **Val 288** | **m+0** | 0.166 | 0.180 | 0.162 | 0.175 | 0.159 | 0.173 |
|  |  | **m+1** | 0.078 | 0.081 | 0.082 | 0.086 | 0.084 | 0.086 |
|  |  | **m+2** | 0.219 | 0.221 | 0.219 | 0.221 | 0.219 | 0.220 |
|  |  | **m+3** | 0.241 | 0.237 | 0.239 | 0.236 | 0.239 | 0.237 |
|  |  | **m+4** | 0.102 | 0.096 | 0.106 | 0.101 | 0.109 | 0.102 |
|  |  | **m+5** | 0.195 | 0.185 | 0.192 | 0.181 | 0.189 | 0.182 |
|  | **Val 260** | **m+0** | 0.184 | 0.196 | 0.180 | 0.192 | 0.179 | 0.190 |
|  |  | **m+1** | 0.076 | 0.085 | 0.083 | 0.092 | 0.085 | 0.091 |
|  |  | **m+2** | 0.395 | 0.392 | 0.389 | 0.386 | 0.388 | 0.387 |
|  |  | **m+3** | 0.119 | 0.115 | 0.125 | 0.121 | 0.127 | 0.122 |
|  |  | **m+4** | 0.225 | 0.212 | 0.222 | 0.209 | 0.221 | 0.210 |
|  | **Thr 404** | **m+0** | 0.184 | 0.193 | 0.168 | 0.180 | 0.156 | 0.167 |
|  |  | **m+1** | 0.195 | 0.199 | 0.185 | 0.191 | 0.190 | 0.190 |
|  |  | **m+2** | 0.172 | 0.172 | 0.205 | 0.204 | 0.212 | 0.219 |
|  |  | **m+3** | 0.237 | 0.232 | 0.231 | 0.227 | 0.228 | 0.227 |
|  |  | **m+4** | 0.213 | 0.204 | 0.211 | 0.198 | 0.214 | 0.197 |
|  | **Thr 376** | **m+0** | 0.207 | 0.223 | 0.193 | 0.210 | 0.182 | 0.199 |
|  |  | **m+1** | 0.235 | 0.238 | 0.244 | 0.248 | 0.252 | 0.254 |
|  |  | **m+2** | 0.305 | 0.289 | 0.306 | 0.296 | 0.303 | 0.298 |
|  |  | **m+3** | 0.254 | 0.251 | 0.257 | 0.246 | 0.263 | 0.248 |
|  |  |  |  |  |  |  |  |  |
|  | **Asp 418** | **m+0** | 0.183 | 0.193 | 0.168 | 0.181 | 0.155 | 0.167 |
|  |  | **m+1** | 0.194 | 0.200 | 0.184 | 0.193 | 0.190 | 0.192 |
|  |  | **m+2** | 0.172 | 0.173 | 0.206 | 0.204 | 0.212 | 0.219 |
|  |  | **m+3** | 0.237 | 0.231 | 0.231 | 0.226 | 0.228 | 0.226 |
|  |  | **m+4** | 0.214 | 0.203 | 0.211 | 0.196 | 0.214 | 0.195 |
|  | **Asp 390** | **m+0** | 0.206 | 0.224 | 0.193 | 0.211 | 0.181 | 0.200 |
|  |  | **m+1** | 0.234 | 0.237 | 0.244 | 0.247 | 0.252 | 0.253 |
|  |  | **m+2** | 0.305 | 0.286 | 0.306 | 0.294 | 0.303 | 0.297 |
|  |  | **m+3** | 0.254 | 0.253 | 0.257 | 0.248 | 0.263 | 0.249 |
| **50 % U-^12^C / 50% U-^13^C labelling** | **Asp 316** | **m+0** | 0.221 | 0.239 | 0.207 | 0.227 | 0.195 | 0.215 |
|  |  | **m+1** | 0.235 | 0.238 | 0.246 | 0.249 | 0.256 | 0.256 |
|  |  | **m+2** | 0.302 | 0.282 | 0.303 | 0.290 | 0.299 | 0.292 |
|  |  | **m+3** | 0.242 | 0.241 | 0.244 | 0.234 | 0.251 | 0.236 |
|  | **Glu 432** | **m+0** | 0.092 | 0.105 | 0.085 | 0.096 | 0.080 | 0.091 |
|  |  | **m+1** | 0.115 | 0.123 | 0.119 | 0.129 | 0.122 | 0.130 |
|  |  | **m+2** | 0.240 | 0.239 | 0.234 | 0.236 | 0.228 | 0.233 |
|  |  | **m+3** | 0.233 | 0.231 | 0.239 | 0.236 | 0.245 | 0.239 |
|  |  | **m+4** | 0.188 | 0.174 | 0.190 | 0.178 | 0.189 | 0.179 |
|  |  | **m+5** | 0.133 | 0.127 | 0.133 | 0.126 | 0.136 | 0.127 |
|  | **Glu 330** | **m+0** | 0.163 | 0.176 | 0.144 | 0.158 | 0.141 | 0.149 |
|  |  | **m+1** | 0.105 | 0.119 | 0.135 | 0.146 | 0.139 | 0.158 |
|  |  | **m+2** | 0.364 | 0.358 | 0.337 | 0.334 | 0.334 | 0.324 |
|  |  | **m+3** | 0.155 | 0.152 | 0.184 | 0.177 | 0.188 | 0.189 |
|  |  | **m+4** | 0.213 | 0.196 | 0.199 | 0.185 | 0.198 | 0.180 |
|  | **Ser 390** | **m+0** | 0.303 | 0.317 | 0.280 | 0.290 | 0.286 | 0.297 |
|  |  | **m+1** | 0.182 | 0.176 | 0.196 | 0.194 | 0.192 | 0.191 |
|  |  | **m+2** | 0.160 | 0.155 | 0.184 | 0.184 | 0.178 | 0.178 |
|  |  | **m+3** | 0.354 | 0.351 | 0.340 | 0.331 | 0.344 | 0.335 |
|  | **Ser 362** | **m+0** | 0.345 | 0.354 | 0.319 | 0.329 | 0.330 | 0.336 |
|  |  | **m+1** | 0.222 | 0.220 | 0.263 | 0.263 | 0.245 | 0.252 |
|  |  | **m+2** | 0.433 | 0.426 | 0.418 | 0.408 | 0.424 | 0.412 |
|  | **Ser 288** | **m+0** | 0.364 | 0.375 | 0.336 | 0.348 | 0.349 | 0.356 |
|  |  | **m+1** | 0.207 | 0.206 | 0.253 | 0.253 | 0.233 | 0.240 |
|  |  | **m+2** | 0.428 | 0.419 | 0.410 | 0.398 | 0.418 | 0.403 |
|  | **Phe 234** | **m+0** | 0.080 | 0.090 | 0.079 | 0.089 | 0.076 | 0.088 |
|  |  | **m+1** | 0.040 | 0.056 | 0.040 | 0.058 | 0.045 | 0.058 |
|  |  | **m+2** | 0.174 | 0.178 | 0.173 | 0.176 | 0.170 | 0.178 |
|  |  | **m+3** | 0.094 | 0.095 | 0.095 | 0.097 | 0.098 | 0.099 |
|  |  | **m+4** | 0.189 | 0.181 | 0.189 | 0.180 | 0.186 | 0.182 |
|  |  | **m+5** | 0.097 | 0.092 | 0.097 | 0.094 | 0.100 | 0.096 |
|  |  | **m+6** | 0.183 | 0.167 | 0.183 | 0.165 | 0.179 | 0.168 |
|  |  | **m+7** | 0.053 | 0.053 | 0.054 | 0.055 | 0.059 | 0.055 |
|  |  | **m+8** | 0.091 | 0.077 | 0.090 | 0.076 | 0.088 | 0.077 |
|  | **Phe 302** | **m+0** | 0.381 | 0.399 | 0.382 | 0.394 | 0.376 | 0.392 |
|  |  | **m+1** | 0.180 | 0.180 | 0.178 | 0.189 | 0.188 | 0.188 |
|  |  | **m+2** | 0.439 | 0.422 | 0.440 | 0.417 | 0.436 | 0.419 |
|  | **Gly 246** | **m+0** | 0.390 | 0.399 | 0.391 | 0.395 | 0.386 | 0.393 |
|  |  | **m+1** | 0.168 | 0.176 | 0.166 | 0.185 | 0.174 | 0.184 |
|  |  | **m+2** | 0.442 | 0.425 | 0.443 | 0.421 | 0.440 | 0.423 |
|  | **Gly 218** | **m+0** | 0.455 | 0.478 | 0.455 | 0.480 | 0.455 | 0.477 |
|  |  | **m+1** | 0.545 | 0.522 | 0.545 | 0.520 | 0.545 | 0.523 |
|  | **Tyr 466** | **m+0** | 0.057 | 0.067 | 0.057 | 0.065 | 0.054 | 0.065 |
|  |  | **m+1** | 0.044 | 0.050 | 0.044 | 0.051 | 0.046 | 0.051 |
|  |  | **m+2** | 0.088 | 0.096 | 0.088 | 0.096 | 0.089 | 0.096 |
|  |  | **m+3** | 0.126 | 0.126 | 0.126 | 0.126 | 0.125 | 0.127 |
|  |  | **m+4** | 0.135 | 0.136 | 0.135 | 0.137 | 0.136 | 0.137 |
|  |  | **m+5** | 0.138 | 0.137 | 0.138 | 0.138 | 0.140 | 0.138 |
|  |  | **m+6** | 0.145 | 0.136 | 0.145 | 0.136 | 0.143 | 0.137 |
|  |  | **m+7** | 0.120 | 0.113 | 0.120 | 0.113 | 0.120 | 0.113 |
|  |  | **m+8** | 0.067 | 0.063 | 0.067 | 0.064 | 0.070 | 0.064 |
|  |  | **m+9** | 0.080 | 0.074 | 0.080 | 0.073 | 0.078 | 0.073 |
|  |  |  |  |  |  |  |  |  |
| **50 % U-^12^C / 50% U-^13^C labelling** | **Tyr 302** | **m+0** | 0.381 | 0.395 | 0.382 | 0.390 | 0.376 | 0.389 |
|  |  | **m+1** | 0.180 | 0.180 | 0.178 | 0.189 | 0.188 | 0.188 |
|  |  | **m+2** | 0.439 | 0.425 | 0.440 | 0.421 | 0.436 | 0.423 |
|  | **Lys 431** | **m+0** | 0.083 | 0.095 | 0.076 | 0.089 | 0.070 | 0.082 |
|  |  | **m+1** | 0.102 | 0.112 | 0.101 | 0.112 | 0.103 | 0.111 |
|  |  | **m+2** | 0.160 | 0.163 | 0.165 | 0.169 | 0.163 | 0.171 |
|  |  | **m+3** | 0.212 | 0.213 | 0.207 | 0.209 | 0.208 | 0.208 |
|  |  | **m+4** | 0.176 | 0.170 | 0.186 | 0.178 | 0.190 | 0.183 |
|  |  | **m+5** | 0.154 | 0.143 | 0.153 | 0.143 | 0.153 | 0.143 |
|  |  | **m+6** | 0.113 | 0.104 | 0.112 | 0.101 | 0.113 | 0.101 |
|  | **Lys 329** | **m+0** | 0.100 | 0.117 | 0.093 | 0.109 | 0.087 | 0.104 |
|  |  | **m+1** | 0.118 | 0.130 | 0.123 | 0.135 | 0.127 | 0.137 |
|  |  | **m+2** | 0.249 | 0.249 | 0.243 | 0.247 | 0.236 | 0.244 |
|  |  | **m+3** | 0.232 | 0.225 | 0.239 | 0.229 | 0.245 | 0.234 |
|  |  | **m+4** | 0.179 | 0.163 | 0.180 | 0.166 | 0.179 | 0.167 |
|  |  | **m+5** | 0.122 | 0.116 | 0.122 | 0.113 | 0.125 | 0.113 |
|  | **HB_255** | **m+0** | - | - | 0.180 | 0.194 | 0.179 | 0.190 |
|  |  | **m+1** | - | - | 0.082 | 0.095 | 0.084 | 0.094 |
|  |  | **m+2** | - | - | 0.390 | 0.385 | 0.388 | 0.385 |
|  |  | **m+3** | - | - | 0.124 | 0.118 | 0.126 | 0.120 |
|  |  | **m+4** | - | - | 0.223 | 0.208 | 0.223 | 0.211 |
|  | **HB_233** | **m+0** | - | - | 0.410 | 0.421 | 0.409 | 0.417 |
|  |  | **m+1** | - | - | 0.133 | 0.141 | 0.135 | 0.141 |
|  |  | **m+2** | - | - | 0.457 | 0.438 | 0.456 | 0.442 |

**Supplementary Table 5: Comparison between measured and simulated labelling patterns of proteinogenic amino acids and intracellular polyhydroxybutyric acid when only labelling data from tracer experiments with 1-^13^C glucose as substrate are used for simulation of metabolic fluxes in cells growing in presence of 0, 0.6 and 1.2 M NaCl.** Simulation with OpenFlux was performed using a modified version of model from Supplemetary Table 3 where pyruvate and phosphoenolpyruvate as well as malate and oxaloacetate were considered as global pools.

|  |  | **0 M NaCl** | | **0.6 M NaCl** | | **1.2 M NaCl** | |
| --- | --- | --- | --- | --- | --- | --- | --- |
|  |  | **Sim** | **Exp** | **Sim** | **Exp** | **Sim** | **Exp** |
| **Ala 260** | **m+0** | 0.508 | 0.507 | 0.526 | 0.526 | 0.548 | 0.548 |
|  | **m+1** | 0.361 | 0.362 | 0.347 | 0.347 | 0.329 | 0.329 |
|  | **m+2** | 0.101 | 0.102 | 0.099 | 0.099 | 0.096 | 0.096 |
| **Ala 232** | **m+0** | 0.531 | 0.529 | 0.549 | 0.548 | 0.573 | 0.571 |
|  | **m+1** | 0.369 | 0.371 | 0.353 | 0.355 | 0.332 | 0.335 |
| **Val 288** | **m+0** | 0.336 | 0.333 | 0.360 | 0.357 | 0.393 | 0.390 |
|  | **m+1** | 0.406 | 0.406 | 0.399 | 0.398 | 0.387 | 0.386 |
|  | **m+2** | 0.188 | 0.191 | 0.176 | 0.179 | 0.161 | 0.164 |
| **Val 260** | **m+0** | 0.342 | 0.338 | 0.366 | 0.363 | 0.401 | 0.396 |
|  | **m+1** | 0.409 | 0.408 | 0.401 | 0.400 | 0.387 | 0.387 |
|  | **m+2** | 0.185 | 0.188 | 0.173 | 0.176 | 0.157 | 0.161 |
| **Thr 404** | **m+0** | 0.346 | 0.345 | 0.352 | 0.351 | 0.360 | 0.364 |
|  | **m+1** | 0.377 | 0.378 | 0.373 | 0.374 | 0.368 | 0.368 |
|  | **m+2** | 0.188 | 0.189 | 0.187 | 0.187 | 0.185 | 0.183 |
| **Thr 376** | **m+0** | 0.374 | 0.374 | 0.384 | 0.383 | 0.398 | 0.398 |
|  | **m+1** | 0.382 | 0.383 | 0.376 | 0.377 | 0.367 | 0.369 |
|  | **m+2** | 0.180 | 0.179 | 0.177 | 0.177 | 0.173 | 0.173 |
| **Asp 418** | **m+0** | 0.345 | 0.346 | 0.351 | 0.351 | 0.359 | 0.364 |
|  | **m+1** | 0.377 | 0.376 | 0.373 | 0.373 | 0.367 | 0.367 |
|  | **m+2** | 0.189 | 0.188 | 0.187 | 0.187 | 0.185 | 0.183 |
| **Asp 390** | **m+0** | 0.374 | 0.374 | 0.383 | 0.383 | 0.397 | 0.397 |
|  | **m+1** | 0.381 | 0.382 | 0.375 | 0.376 | 0.367 | 0.368 |
|  | **m+2** | 0.180 | 0.180 | 0.178 | 0.178 | 0.174 | 0.173 |
| **Asp 316** | **m+0** | 0.411 | 0.408 | 0.421 | 0.417 | 0.437 | 0.433 |
|  | **m+1** | 0.389 | 0.386 | 0.382 | 0.380 | 0.371 | 0.370 |
|  | **m+2** | 0.154 | 0.154 | 0.152 | 0.153 | 0.148 | 0.148 |
| **Glu 432** | **m+0** | 0.243 | 0.247 | 0.258 | 0.260 | 0.280 | 0.281 |
|  | **m+1** | 0.369 | 0.368 | 0.368 | 0.367 | 0.366 | 0.365 |
|  | **m+2** | 0.243 | 0.241 | 0.235 | 0.234 | 0.224 | 0.224 |
| **Glu 330** | **m+0** | 0.322 | 0.323 | 0.342 | 0.343 | 0.365 | 0.371 |
|  | **m+1** | 0.404 | 0.402 | 0.397 | 0.396 | 0.387 | 0.386 |
|  | **m+2** | 0.198 | 0.199 | 0.188 | 0.189 | 0.179 | 0.176 |
| **Ser 390** | **m+0** | 0.442 | 0.448 | 0.458 | 0.465 | 0.477 | 0.482 |
|  | **m+1** | 0.365 | 0.363 | 0.354 | 0.351 | 0.341 | 0.338 |
|  | **m+2** | 0.143 | 0.141 | 0.140 | 0.138 | 0.136 | 0.135 |
|  |  |  |  |  |  |  |  |
|  |  |  |  |  |  |  |  |
|  |  |  |  |  |  |  |  |
|  |  | **0 M NaCl** | | **0.6 M NaCl** | | **1.2 M NaCl** | |
|  |  | **Sim** | **Exp** | **Sim** | **Exp** | **Sim** | **Exp** |
| **Ser 362** | **m+0** | 0.471 | 0.476 | 0.487 | 0.493 | 0.508 | 0.512 |
|  | **m+1** | 0.383 | 0.380 | 0.370 | 0.366 | 0.354 | 0.352 |
| **Ser 288** | **m+0** | 0.511 | 0.515 | 0.528 | 0.534 | 0.552 | 0.554 |
|  | **m+1** | 0.377 | 0.374 | 0.363 | 0.358 | 0.344 | 0.341 |
| **Phe 234** | **m+0** | 0.334 | 0.334 | 0.359 | 0.360 | 0.394 | 0.391 |
|  | **m+1** | 0.419 | 0.411 | 0.412 | 0.405 | 0.401 | 0.396 |
|  | **m+2** | 0.192 | 0.192 | 0.178 | 0.178 | 0.160 | 0.163 |
| **Phe 302** | **m+0** | 0.731 | 0.732 | 0.731 | 0.731 | 0.729 | 0.729 |
|  | **m+1** | 0.194 | 0.194 | 0.194 | 0.194 | 0.196 | 0.196 |
| **Gly 246** | **m+0** | 0.764 | 0.759 | 0.763 | 0.759 | 0.761 | 0.758 |
|  | **m+1** | 0.166 | 0.171 | 0.167 | 0.171 | 0.169 | 0.171 |
| **Gly 218** | **m+0** | 0.829 | 0.828 | 0.829 | 0.828 | 0.829 | 0.827 |
|  | **m+1** | 0.171 | 0.172 | 0.171 | 0.172 | 0.171 | 0.173 |
| **Tyr 466** | **m+0** | 0.255 | 0.257 | 0.274 | 0.278 | 0.299 | 0.298 |
|  | **m+1** | 0.372 | 0.368 | 0.371 | 0.367 | 0.368 | 0.364 |
|  | **m+2** | 0.235 | 0.231 | 0.225 | 0.221 | 0.213 | 0.211 |
| **Tyr 302** | **m+0** | 0.731 | 0.732 | 0.731 | 0.732 | 0.729 | 0.731 |
|  | **m+1** | 0.194 | 0.193 | 0.194 | 0.194 | 0.196 | 0.195 |
| **Lys 431** | **m+0** | 0.234 | 0.240 | 0.247 | 0.254 | 0.266 | 0.274 |
|  | **m+1** | 0.366 | 0.363 | 0.366 | 0.362 | 0.364 | 0.362 |
|  | **m+2** | 0.248 | 0.245 | 0.241 | 0.238 | 0.231 | 0.227 |
| **Lys 329** | **m+0** | 0.270 | 0.272 | 0.287 | 0.288 | 0.312 | 0.310 |
|  | **m+1** | 0.392 | 0.383 | 0.390 | 0.382 | 0.386 | 0.377 |
|  | **m+2** | 0.231 | 0.234 | 0.222 | 0.224 | 0.209 | 0.213 |
| **HB 275** | **m+0** | - | - | 0.366 | 0.364 | 0.401 | 0.399 |
|  | **m+1** | - | - | 0.399 | 0.399 | 0.386 | 0.386 |
|  | **m+2** | - | - | 0.173 | 0.175 | 0.158 | 0.159 |
| **HB 233** | **m+0** | - | - | 0.550 | 0.549 | 0.575 | 0.573 |
|  | **m+1** | - | - | 0.352 | 0.354 | 0.331 | 0.333 |

**Supplementary Table 6: Precursor demand [μmol g_CDW_^-1^] for the wild-type *B. megaterium* growing in M9 minimal medium in presence of up to 1.8 M NaCl.** Pecursor demand for cells growing in M9 minimal medium supplemented with 0.3, 0.9 and 1.8 M NaCl were extrapolled from data at 0, 0.6 and 1.2 M NaCl.


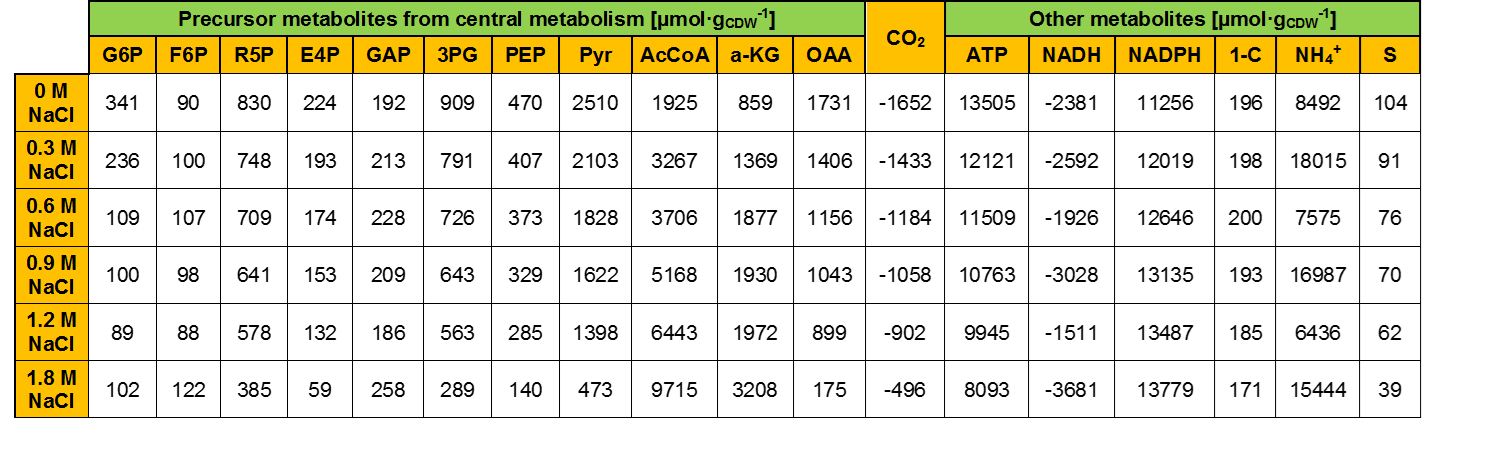


**References**

AOCS (1999). "Official method 991.39," in *Official methods and recommended practices of the AOCS,* ed. D. Firestone. 5th ed (AOCS, Champaign, IL).

Benthin, S., Nielsen, J., and Villadsen, J. (1991). A simple and reliable method for the determination of cellular RNA content. *Biotechnol Tech* 5(1), 39-42. doi: 10.1007/BF00152753.

Folch, J., Lees, M., and Sloane Stanley, G.H. (1957). A simple method for the isolation and purification of total lipids from animal tissues. *J Biol Chem* 226(1), 497-509.

Fountoulakis, M., and Lahm, H.W. (1998). Hydrolysis and amino acid composition of proteins. *J Chromatogr A* 826(2), 109-134. doi: 10.1016/S0021-9673(98)00721-3.

Goldfarb, A.R., Saidel, L.J., and Mosovich, E. (1951). The ultraviolet absorption spectra of proteins. *J Biol Chem* 193(1), 397-404.

Kohlstedt, M., Sappa, P.K., Meyer, H., Maass, S., Zaprasis, A., Hoffmann, T., et al. (2014). Adaptation of *Bacillus subtilis* carbon core metabolism to simultaneous nutrient limitation and osmotic challenge: a multi‐omics perspective. *Environ Microbiol* 16(6), 1898-1917. doi: 10.1111/1462-2920.12438.

Tombs, M.P., Souter, F., and Maclagan, N.F. (1959). The spectrophotometric determination of protein at 210 millimicrons. *Biochem J* 73, 167-171.

Van Heijenoort, J., Elbaz, L., Dezelee, P., Petit, J.F., Bricas, E., and Ghuysen, J.M. (1969). Structure of the meso-diaminopimelic acid containing peptidoglycans in *Escherichia coli* B and *Bacillus megaterium* KM. *Biochemistry* 8(1), 207-213. doi: 10.1021/bi00829a030.

Vollmer, W., Blanot, D., and de Pedro, M.A. (2008). Peptidoglycan structure and architecture. *FEMS Microbiol Rev* 32(2), 149-167. doi: 10.1111/j.1574-6976.2007.00094.x.

Wolf, P. (1983). A critical reappraisal of Waddell's technique for ultraviolet spectrophotometric protein estimation. *Anal Biochem* 129(1), 145-155. doi: 10.1016/0003-2697(83)90062-3.
